# Dissecting causal relationships between primary biliary cholangitis and extrahepatic autoimmune diseases based on Mendelian randomization

**DOI:** 10.1038/s41598-024-62509-x

**Published:** 2024-05-21

**Authors:** Gang Ma, Jiaqi Yang, Xingguo Wang, Erzhuo Xia, Jiahao Yu, Miao Zhang, Yinan Hu, Shuoyi Ma, Xia Zhou, Qingling Fan, Ying Han, Jingbo Wang

**Affiliations:** 1 Xijing Hospital of Digestive Disease, Xijing Hospital of Air Force Military Medical University, Xi’an, China; 2 Xijing Hospital of Obstetrics and Gynecology, Xijing Hospital of Air Force Military Medical University, Xi’an, China

**Keywords:** Primary biliary cholangitis, Extrahepatic autoimmune diseases, Mendelian randomization, Genetic epidemiology, Causal effect, Quality of life, Diseases, Risk factors, Signs and symptoms

## Abstract

As an autoimmune disease, up to 73% of patients with primary biliary cholangitis (PBC) have a combination of extrahepatic autoimmune diseases (EHAIDs); however, the causal relationship between PBC and EHAIDs is unclear. The genome-wide association analyses provided 14 GWAS data for PBC and EHAIDs, and bidirectional, two-sample MR analyses were performed to examine the relationship between PBC and EHAIDs. The analysis using MR provides a strong and meaningful estimation of the bidirectional correlation between PBC and 7 EHAIDs: rheumatoid arthritis, systemic lupus erythematosus, Sjögren's syndrome, systemic sclerosis, autoimmune hypothyroidism, inflammatory bowel disease and ulcerative colitis of its types. In addition, PBC increases the risk of autoimmune thyroid diseases such as autoimmune hyperthyroidism and Graves' disease, as well as multiple sclerosis and psoriasis. Additionally, PBC is identified as a risk factor for Crohn's disease and Celiac disease. Based on genetic evidence, there may be connections between PBC and specific EHAIDs: not all coexisting EHAIDs induce PBC, and vice versa. This underscores the significance of prioritizing PBC in clinical practice. Additionally, if any liver function abnormalities are observed during treatment or with EHAIDs, it is crucial to consider the possibility of comorbid PBC.

## Introduction

Epidemiological studies may be biased due to factors that cannot be measured, such as unmeasurable confounding and reverse causality. These factors make it challenging to make specific causal inferences. Although epidemiological studies indicate a link between PBC and EHAIDs, it is still uncertain whether this association is causal. Mendelian randomization (MR) is a statistical analysis that helps infer the cause of diseases based on genetic variants. MR analysis uses single nucleotide polymorphisms (SNPs) as instrumental variables (IV) to explore potential causal relationships between exposure (phenotype) and outcome (disease). These genetic variants are randomly assigned at conception, which minimizes potential bias due to environmental confounders and reverse causality. Therefore, MR analysis is a reliable method to investigate the causal relationship between exposure and outcome^6,7^.

A recent study by Fan et al.^8^ indicated that PBC may have a causal role in the pathogenesis of RA, but not vice versa. However, the study did not account for the possible influence of MHC regions on the outcome, which could have led to a bias. In the MR study of IBD on PBC^9^, the authors did not utilize the latest GWAS data for PBC, and the impact of UC on PBC was inconsistent. As a result, the findings may not be entirely accurate and require validation with a larger sample size. In addition, there has been little research on the relationship between PBC and other EHAIDs. To address this gap, we conducted a two-sample bidirectional MR study on major EHAIDs with PBC to better understand the causal effects.

## Methods

### Study cohorts and GWAS

We searched PubMed to identify EHAIDs with a relatively high incidence of comorbidity with PBC: rheumatoid arthritis (RA), systemic lupus erythematosus (SLE), Sjögren's syndrome (SS), systemic sclerosis (SSc), autoimmune thyroid disease (AITD), autoimmune hyperthyroidism (AHYPER), Graves' disease (GD), autoimmune hypothyroidism (AHYPO), inflammatory bowel disease (IBD), Crohn's disease (CD), ulcerative colitis (UC), celiac disease (CeD), multiple sclerosis (MS), and psoriasis (PoS). We obtained GWAS data for PBC and EHAIDs from PubMed and online platforms such as GWAS catalog, IEU Open GWAS project, UKBB, and Finngen R9 (Table 1). When there were multiple GWAS options for a disease, we chose the best one based on these criteria: all cases and controls were of European origin, no significant overlap between GWAS populations, complete GWAS pooled data, high number of cases, and relatively high number of SNPs detected.

**Table 1:** Summary of PBC and EHAIDs.

Diseases	GWAS PMID^a^	Case number	Control number	Web Source^b^	MR study	
					PBC on EHAID	EHAID on PBC
Primary biliary cholangitis	34033851	8021	16,489	GWAS-catalog: GCST90061440		
Rheumatoid arthritis	24390342	14,361	42,923	GWAS-catalog: GCST002318	Positive	Positive
Systemic lupus erythematosus	26502338	5201	9066	GWAS-catalog: GCST003156	Positive	Positive
Sjögren's syndrome	28076899	585	1546	GWAS-catalog: GCST012796	Positive	Positive
Systemic sclerosis	31672989	9095	17,584	GWAS-catalog: GCST009131	Positive	Positive
Autoimmune thyroid disease	32581359	30,234	725,172	deCODE	Positive	Null
Autoimmune hyperthyroidism	FinnGen	1828	279,855	FinnGen-R9: AUTOIMMUNE_HYPERTHYROIDISM	Positive	Null
Graves' disease	FinnGen	2836	374,441	FinnGen-R9: E4_GRAVES_STRICT	Positive	Null
Autoimmune hypothyroidism	FinnGen	40,926	274,069	FinnGen-R9: E4_HYTHY_AI_STRICT	Positive	Positive
Inflammatory bowel disease	26192919	31,665	33,977	MR-base: ieu-a-294	Positive	Positive
Crohn's disease	26192919	17,897	33,977	MR-base: ieu-a-12	Null	Positive
Ulcerative colitis	26192919	13,768	33,977	MR-base: ieu-a-970	Positive	Positive
Celiac disease	22057235	11,812	11,837	GWAS-catalog: GCST005523	Null	Positive
Multiple sclerosis	31604244	47,429	68,374	MR-base: ieu-b-18	Positive	Null
Psoriasis	34927100	15,967	28,194	GWAS-catalog: GCST90019016	Positive	Null

^a^PubMed ID for GWAS of outcomes. FinnGen indicates that the GWAS was derived from the FinnGen research project.

^b^MR-base: https://gwas.mrcieu.ac.uk/; GWAS catalog: https://www.ebi.ac.uk/gwas/;

FinnGen-R9: https://r9.finngen.fi/; deCODE: https://www.decode.com/summarydata/.

### Mendelian randomization

#### IV selection

When conducting an MR study, it is crucial to use validated genetic variants (SNPs) that meet specific criteria. First, they should be strongly associated with the exposure being studied. Second, there should be no common cause for the outcome. Last, the SNPs should only affect the outcome through the exposure pathway and not through any confounding factors^7^. In summary, the SNPs must be related to the exposure, unrelated to any confounders, and not affect the outcome or confounding factors.

To choose genetic instruments from each of the 14 exposed GWASs, we utilized the default settings of TwoSampleMR from the R package^10,11^. Specifically, our approach involved extracting SNPs that are genome-wide significant (P value < 5.0E−08) while excluding those containing MHC regions^12^. We used standard aggregation parameters that excluded variants with a physical distance of less than 10,000 kb and r^2^ < 0.001. When IVs were present in the exposure but not in the outcome, we relied on the LDproxyR tool to replace them with proxy SNPs in high linkage disequilibrium (LD r^2^ > 0.8). To estimate the intensity of IV, we calculated the proportion of variance in the exposure explained by the SNP (r^2^) and the F-statistic^13^. An F-statistic > 10 indicated the appropriate instrumental variable to satisfy the first MR hypothesis. We excluded ambiguous SNPs with inconsistent alleles, echo SNPs with ambiguous chains and SNPs with minor allele frequencies less than 1%. Finally, we utilized IV clumping and then applied Steiger filtering to eliminate SNPs that accounted for more variability in the outcome than in the exposure^14^.

**Figure 1:**
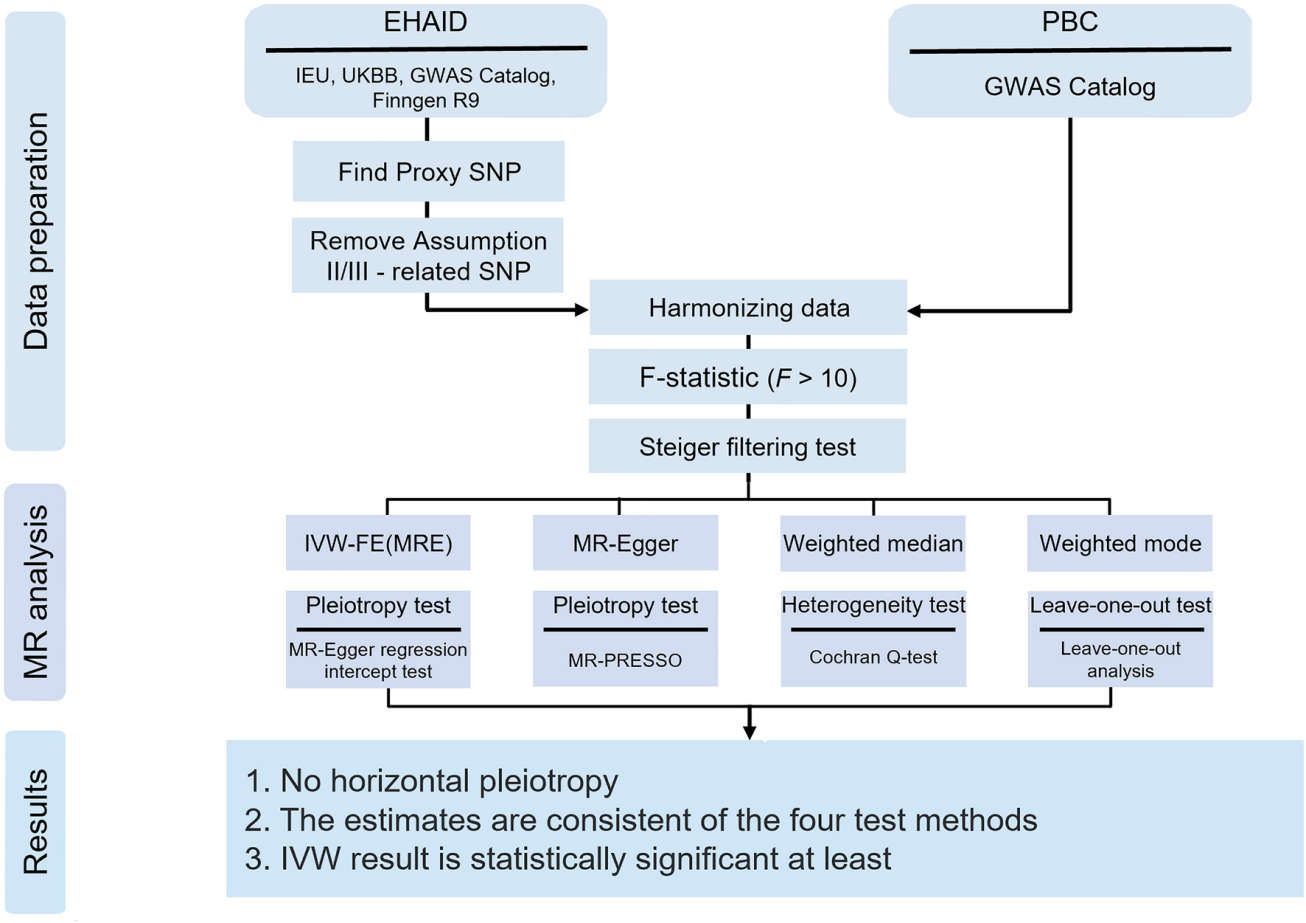
Flowchart for Mendelian randomization analysis.

This approach was also applied to the reverse MR process, resulting in a total of 28 two-way MR studies that evaluated EHAIDs and PBC as both exposures and outcomes (Fig. 1).

#### Mendelian randomization analyses

We conducted a thorough analysis of MR using four different methods, each with varying statistical assumptions: inverse variance weighted method with fixed effects and multiplicative random effects (IVW-FE and IVW-MRE), MR‒Egger regression, weighted median method (WME), and weighted mode method (WM). Our aim was to obtain conclusive β estimates that could be converted to ORs^15–17^. To test heterogeneity, we utilized the Cochran Q test in the IVW and MR‒Egger methods^18^. If the IVW method indicated heterogeneity, we used IVW-MRE. We also employed the MR‒Egger regression intercept method and the Global Test of MR-PRESSO to evaluate the horizontal multiplicity of IVs. To ensure reliable results and eliminate outliers, we conducted MR-PRESSO^19,20^ and MR analyses multiple times. We removed the outlier corresponding to the smallest P value of MR-PRESSO each time unless all P values were 1. The final MR results were generated when the P values of the pleiotropy test (MR-Egger intercept and MR-PRESSO global test) and the heterogeneity test (IVW Cochran Q test) were greater than 0.05. This approach helped us minimize outliers and retain valid instrumental variables for reliable results. To visually present the results of various MR methods, we created scatter plots using the TwoSampleMR package. Additionally, we conducted a leave-one-out analysis (LOO) to detect the potential impact of any single SNP on the outcome of each MR study. In the absence of horizontal pleiotropy in instrumental variables, we relied on the IVW method as our primary detection tool. To minimize type I errors, we applied FDR correction to the p-values of MR. If the adjusted P-value was less than 0.05, we considered it as a significant genetic causal relationship between the exposure and outcome. If the P-value was less than 0.05 corrected but adjusted P-value not, we considered it as a potential genetic causal relationship between exposure and outcome (Fig. 1).

All statistical analyses were carried out using the R program (version 4.3.1) and the TwoSampleMR^11^, MRPRESSO^19^ packages.

## Results

### Results of the MR analyses of PBC on EHAIDs

**Figure 2:**
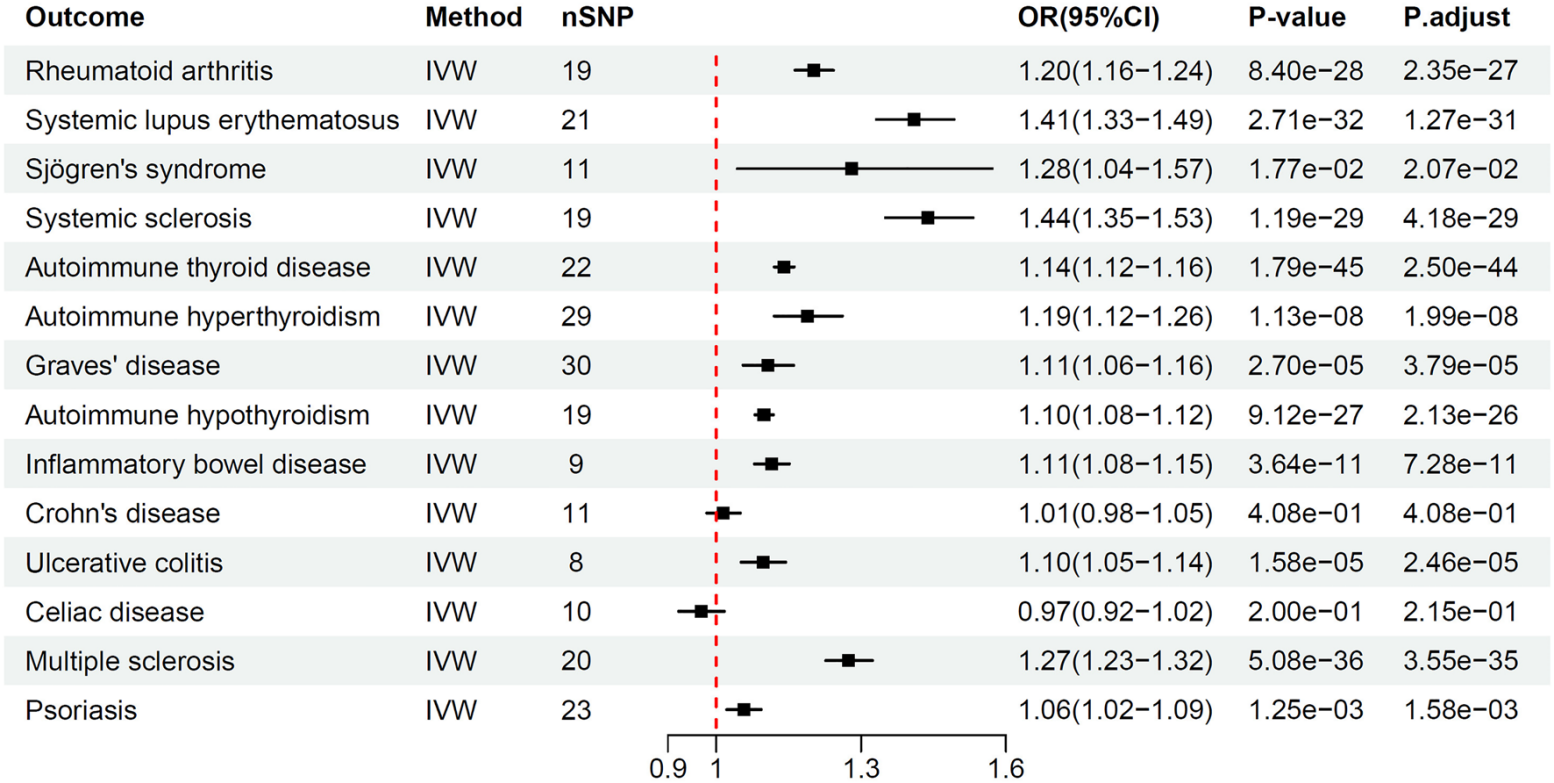
IVW results of Mendelian randomization analysis for PBC-on-EHAIDs.

The main results of MR analysis investigating the relationship between PBC and EHAIDs are shown in Table 2. PBC was considered significant with a value of P < 5.0E−08 as an exposure factor, and 8–30 eligible SNPs were obtained (Supplementary 2, S2T1). These SNPs accounted for 3.05% to 11.38% of the total variance in PBC. The F values for individual SNPs and mean-F statistics were > 10, indicating that the instrument strength was adequate. Our IVW results showed that PBC has a positive genetic contribution to EHAIDs, including RA, SLE, SS, SSc, AITD, AHYPO, AHYPER, GD, IBD, UC, MS and PoS, which may increase the risk of developing these diseases, with ORs ranging from 1.06 to 1.44 (Fig. 2). Furthermore, the Steiger test confirmed the directionality of causality, as shown in the Supplementary 1, S1T3.

**Table 2:** Main Results of the MR analyses PBC on EHAIDs.

Outcome	nSNPs	r2	F	Inverse variance weighted		MR Egger		Weighted median		Weighted mode		Steiger directionality	
				OR (95% CI)	P-value	OR (95% CI)	P-value	OR (95% CI)	P-value	OR (95% CI)	P-value	Correct causal direction	P-value
RA	19	6.69%	92.38	1.20 (1.16–1.24)	8.40E−28	1.16(1.04–1.29)	1.88E−02	1.19(1.13–1.25)	1.44E−11	1.19 1.12–1.27)	4.29E−05	TRUE	1.65E−172
SLE	21	8.34%	106.15	1.41 (1.33–1.49)	2.71E−32	1.37 (1.13–1.66)	5.05E−03	1.42 (1.31–1.54)	8.04E−17	1.43 (1.28–1.60)	4.33E−06	TRUE	1.50E−71
SS	11	3.38%	77.91	1.28 (1.04–1.57)	1.77E−02	1.10 (0.40–3.02)	8.58E−01	1.27 (0.96–1.69)	9.65E−02	1.30 (0.86–1.96)	2.47E−01	TRUE	2.67E−06
SSc	19	7.65%	106.82	1.44 (1.35–1.53)	1.19E−29	1.54 (1.22–1.93)	1.78E−03	1.49 (1.35–1.64)	3.68E−15	1.52 (1.34–1.72)	3.49E−06	TRUE	1.47E−121
AITD	22	8.17%	99.07	1.14 (1.12–1.16)	1.79E−45	1.11 (1.05–1.18)	1.55E−03	1.13 (1.10–1.16)	3.44E−17	1.12 (1.08–1.16)	1.79E−06	TRUE	0.00E+00
AHYPER	29	11.20%	106.42	1.19 (1.12–1.26)	1.13E−08	1.13 (0.93–1.38)	2.26E−01	1.16 (1.07–1.27)	5.47E−04	1.16 (1.02–1.32)	3.25E−02	TRUE	0.00E+00
GD	30	11.38%	104.81	1.11 (1.06–1.16)	2.70E−05	1.04 (0.88–1.23)	6.27E−01	1.09 (1.02–1.17)	7.84E−03	1.07 (0.97–1.19)	1.85E−01	TRUE	0.00E+00
AHYPO	19	7.42%	103.36	1.10 (1.08–1.12)	9.12E−27	1.10 (1.04–1.17)	7.02E−03	1.09 (1.06–1.12)	3.46E−10	1.09 (1.05–1.13)	2.83E−04	TRUE	0.00E+00
IBD	9	3.25%	91.46	1.11 (1.08–1.15)	3.64E−11	1.12 (0.96–1.31)	1.88E−01	1.13 (1.08–1.18)	1.82E−07	1.14 (1.06–1.21)	5.45E−03	TRUE	3.33E−92
CD	11	4.33%	100.69	1.01 (0.98–1.05)	4.08E−01	1.05 (0.92–1.20)	5.07E−01	1.03 (0.98–1.08)	2.59E−01	1.02 (0.96–1.08)	6.31E−01	TRUE	2.97E−138
UC	8	3.05%	96.35	1.10 (1.05–1.14)	1.58E−05	1.19 (1.01–1.41)	8.32E−02	1.08 (1.02–1.15)	5.90E−03	1.11 (1.02–1.20)	5.09E−02	TRUE	7.59E−83
CeD	10	4.53%	116.26	0.97 (0.92–1.02)	2.00E−01	0.94 (0.75–1.17)	5.78E−01	0.97 (0.91–1.04)	3.59E−01	0.97 (0.90–1.05)	4.49E−01	TRUE	6.29E−59
MS	20	6.59%	86.39	1.27 (1.23–1.32)	5.08E−36	1.31 (1.14–1.50)	1.10E−03	1.22 (1.15–1.30)	1.07E−10	1.21 (1.09–1.33)	1.32E−03	TRUE	7.21E−220
PoS	23	7.85%	90.64	1.06 (1.02–1.09)	1.25E−03	1.15 (1.04–1.28)	1.63E−02	1.08 (1.03–1.14)	2.66E−03	1.08 (1.01–1.16)	3.07E−02	TRUE	2.03E−228

### Results of the MR analyses of EHAIDs on PBC

**Figure 3:**
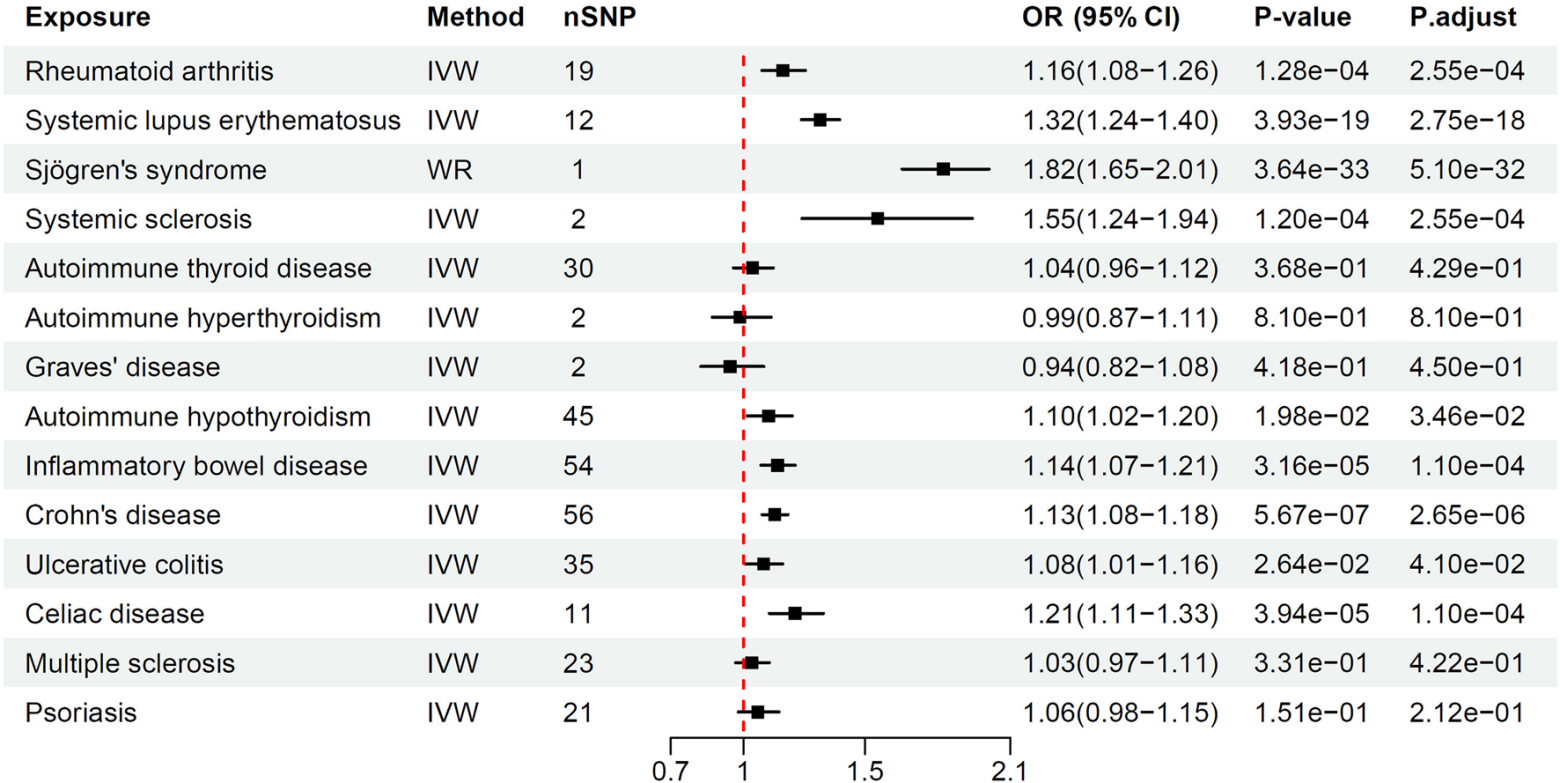
IVW results of Mendelian randomization analysis for EHAIDs on PBC.

Table 3 shows the results of EHAIDs on PBC. The eligible SNPs for EHAIDs ranged from 1 to 56, explaining 0.01% to 9.77% of the total variance in the above EHAIDs, with F values for individual SNPs and all mean-F statistics being > 10 (Supplementary 2, S2T2). Our research results showed that RA, SLE, SS, SSc, ATHYO, IBD, CD, UC and CeD contributed positively to PBC pathogenesis. The estimated values (OR) ranged from 1.08 to 1.82. AITD, AHYPER, GD, MS, and PoS did not have a genetically positive contribution to PBC development (Fig. 3). The Steiger test confirmed the directionality of causality (Supplementary 1, S1T3).

**Table 3:** Main results of the MR analyses EHAIDs on PBC.

Exposure	nSNPs	r^2^	F	Inverse variance weighted		MR Egger		Weighted median		Weighted mode		Steiger directionality	
				OR (95% CI)	P-value	OR (95% CI)	P-value	OR (95% CI)	P-value	OR (95% CI)	P-value	Correct causal direction	P-value
RA	19	2.98%	92.45	1.16 (1.08–1.26)	1.28E−04	1.23 (1.03–1.46)	3.47E−02	1.18 (1.06–1.32)	2.14E−03	1.19 (1.07–1.33)	4.86E−03	TRUE	1.38E−67
SLE	12	4.24%	52.59	1.32 (1.24–1.40)	3.93E−19	1.23 (0.99–1.52)	9.38E−02	1.37 (1.26–1.48)	2.52E−13	1.39 (1.24–1.56)	1.47E−04	TRUE	7.05E−44
SS^#^	1	1.96%	42.62	1.82 (1.65–2.01)	3.64E−33							TRUE	4.35E−03
SSc	2	0.27%	36.26	1.55 (1.24–1.94)	1.20E−04							TRUE	1.86E−03
AITD	30	0.31%	78.22	1.04 (0.96–1.12)	3.68E−01	1.19 (0.96–1.49)	1.26E−01	1.02 (0.90–1.14)	7.87E-01	1.01 (0.90–1.14)	8.51E−01	TRUE	1.57E−04
AHYPER	2	0.04%	56.35	0.99 (0.87–1.11)	8.10E−01							TRUE	8.30E−03
GD	2	0.05%	87.77	0.94 (0.82–1.08)	4.18E−01							TRUE	1.35E−02
AHYPO	45	1.48%	105.37	1.10 (1.02–1.20)	1.98E−02	1.31 (1.07–1.60)	1.21E−02	1.09 (0.94–1.27)	2.37E−01	1.11 (0.94–1.32)	2.22E−01	TRUE	3.46E−27
IBD	54	7.31%	95.78	1.14 (1.07–1.21)	3.16E−05	1.11 (0.91–1.35)	3.23E−01	1.09 (0.99–1.20)	8.79E−02	1.07 (0.94–1.22)	2.95E−01	TRUE	4.68E−185
CD	56	10.73%	111.27	1.13 (1.08–1.18)	5.67E−07	1.11 (0.97–1.27)	1.22E−01	1.10 (1.02–1.19)	1.06E−02	1.11 (1.02–1.20)	2.00E−02	TRUE	1.25E−279
UC	35	5.01%	71.84	1.08 (1.01–1.16)	2.64E−02	1.00 (0.80–1.25)	9.78E−01	1.08 (0.97–1.20)	1.89E−01	1.08 (0.93–1.25)	3.22E−01	TRUE	5.15E−120
CeD	11	5.08%	58.58	1.21 (1.11–1.33)	3.94E−05	1.74 (0.98–3.10)	9.28E−02	1.25 (1.10–1.43)	8.95E−04	1.32 (1.07–1.63)	2.61E−02	TRUE	8.10E−67
MS	23	0.94%	47.85	1.03 (0.97–1.11)	3.31E−01	1.02 (0.75–1.38)	9.24E−01	1.05 (0.94–1.17)	4.01E−01	1.02 (0.91–1.16)	7.16E−01	TRUE	7.12E−19
PoS	21	2.56%	55.18	1.06 (0.98–1.15)	1.51E−01	1.19 (0.86–1.65)	3.18E−01	1.09 (0.97–1.22)	1.55E−01	1.13 (0.94–1.35)	1.97E−01	TRUE	2.59E−55

### Sensitivity analyses

Estimates for the other three MR methods were consistent with IVW except for SSc as exposure and shown in the supplementary table except that four exposures were left with less than three SNP (SS, SSc, AHYPER, GD) after conducting MR-PRESSO inspection (Tables 2 and 3, Supplementary 3). MR Egger's intercept method and MR-PRESSO's Global Test were conducted to detect the presence of horizontal pleiotropy. Our findings indicated that the P values were greater than 0.05 for all tests of horizontal pleiotropy with EHAIDs as exposure or outcome for both MR‒Egger intercept and Global Test of MR-PESSO (Supplementary 1, S1T1). And all P values of heterogeneity were greater than 0.05, which increased the reliability of the results (Supplementary 1, S1T2). We also included LOO plots, forest plots, and funnel plots for IVs to visually demonstrate the outlier distribution and contribution effect of each (Supplementary 4–6).

## Discussion

Mendelian randomization analysis, with its unique advantages and insights into etiology, has been applied to the study of factors influencing autoimmune diseases^21^. In this study, we performed a comprehensive two-way two-sample MR study to reveal the causal relationship between the risk of several EHAIDs and PBC. Using this approach, we found a genetic causal relationship between RA, SLE, SS, SSc, AHYPO, IBD, CD, UC and CeD, which are responsible for PBC. Moreover, we found that PBC had a causal association with EHAIDs, including RA, SLE, SS, SSc, AITD, AHYPO, AHYPER, GD, IBD, UC, MS, and PoS. These findings align with the results reported by the epidemiological study, further substantiating the potential explanation for the cooccurrence of PBC and EHAIDs. To ensure the robustness and validity of our MR analyses, we conducted multiple sensitivity analyses. These analyses confirmed that our MR hypotheses were satisfied and allowed us to rule out any moderate to severe causal effect of exposure on the outcome. Given the consistency of MR results across these different methods, we have confidence in the reliability of our findings in elucidating the intricate relationship between PBC and EHAIDs.

The development of rheumatic diseases is often accompanied by abnormal liver function, which may be associated with the presence of coexisting autoimmune liver disease, direct involvement of liver parenchyma or drug therapy^22,23^. As many as 50% of patients with RA and 60% of patients with SLE may have elevated liver enzymes, 49% of patients with SS have abnormal liver serologic parameters, only 1.1% of patients with SSc have liver involvement, and PBC accounts for approximately 22% of patients with liver involvement^23^.

Fan et al.^8^ conducted a study exploring the causal relationship between RA and autoimmune liver disease. Utilizing self-reported GWAS data with a sample size of 5201 RA cases and 457,732 controls, they identified a unidirectional reverse causality between RA and PBC. Similarly, Liu et al.^24^ reported identical findings using aggregated data from populations in the UK and Finland. However, their research did not account for the potential interference of MHC region SNPs on the outcomes. Moreover, Liu et al.^24^ utilized the IVW random effects model, disregarding the potential impact of heterogeneity on the results. In contrast, our MR analysis used a larger sample size of the RA GWAS dataset and found a bidirectional causal relationship between PBC and RA. Our findings suggest that RA increases the prevalence of PBC by 16%, and PBC, in turn, increases the likelihood of RA by 20%. These results indicate a reciprocal causal relationship between PBC and RA. In clinical settings, PBC is the most common EHAID coexisting with RA, with a prevalence of PBC in RA ranging from 3.8 to 6.3% and RA in PBC ranging from 1.8 to 13%^8^. Our two-sample MR analysis suggests that this correlation may not be coincidental.

SLE is a complex autoimmune disease that affects multiple organs, including the liver. According to a study conducted by Gershwin et al.^25^, out of 1032 patients with PBC, 27 (2.61%) also suffered from SLE. The prevalence of SLE was significantly higher in patients with PBC (27/1032, 2.61%) than in the control group (5/1041, 0.48%). Although most articles on SLE and PBC comorbidity are mainly case reports, Huang et al.^26^ summarized 34 cases of SLE combined with PBC. They found that PBC was more common in middle-aged women, while SLE usually affected women of childbearing age. The interval from PBC diagnosis to SLE diagnosis ranged from 7 months to 10 years, with PBC diagnosed 1–19 years after SLE diagnosis. In previously reported MR studies, Liu et al.^24^ reported a unidirectional promoting effect of PBC on SLE without considering heterogeneity. However, Huang et al.^27^ found that after excluding outliers, their MR results indicated that SLE significantly increased the risk of PBC (OR 1.31, P < 0.01). Similarly, by excluding confounding factors and reverse causality, our results show that SLE and PBC are risk factors for each other. Consistent with our findings, Wu et al.^28^ conducted a bidirectional MR analysis using different GWAS data for PBC and found a causal relationship between SLE and PBC, suggesting they mutually promote disease occurrence. Their use of transcriptome overlap analysis further demonstrated the close relationship between PBC and SLE. Therefore, patients with SLE or PBC should be regularly monitored for the possibility of SLE-PBC overlap, even if the onset may occur several years later.

Primary SS (pSS) and secondary SS (sSS) are two subtypes of SS. The clinical and immunological features of PBC and SS are similar, and it has been suggested that PBC is the biliary phenotype of SS, while SS is the exocrine phenotype of PBC^29^. Studies have revealed that SS is the most common autoimmune disease associated with PBC, with a prevalence of 35% according to a meta-analysis^30^. Our MR analysis indicates that PBC genetically promotes SS, and vice versa, even though only one valid SNP was identified. However, in the MR study reported by Liu et al.^24^, the authors did not find a positive causal relationship between SS and PBC. The relatively small number of cases and a large number of controls could have made it difficult to detect disease-related genetic variations, potentially introducing bias into the study's results and preventing an accurate assessment of the relationship between genetic variations and the disease. Further studies on the causal relationship between SS and PBC are needed, especially with regard to subgroup data on pSS and sSS. Since PBC patients are more likely to develop Sicca symptoms, they should be screened early for symptomatic treatment*5*. Those with SS presenting with cholestasis should be promptly screened for PBC comorbidity and UDCA to improve cholestasis, with special attention to AMA-negative PBC patients^31^.

Studies estimate that approximately 2–18% of patients with PBC also have SSc, while approximately 2–10% of SSc patients have PBC^32^. Hepatobiliary issues were found in 7.5% of patients in a large Spanish registry, with PBC being the main cause (4.3%). In PBC patients, characteristic SSc anti-mitochondrial antibodies (ACAs) were present in 9–30%, widespread PBC antibodies were also detectable in SSc patients, and PBC disease has the potential to occur ten years after onset^33^. Our findings suggest that PBC and SSc may increase the risk of each other, which aligns with clinical observations. However, in the MR study reported by Liu et al.^24^, the authors did not find a positive causal relationship between SSc and PBC, which could be related to the relatively small number of cases and the large number of controls. Screening for PBC overlap is recommended when encountering SSc patients in the clinic, as the rate of ACA positivity in SSc-PBC patients is higher than that in PBC cases alone, and early control of gastroesophageal varices may improve prognosis in these patients^32^. Further confirmation through observation of a larger clinical cohort and GWAS dataset is necessary.

Patients diagnosed with PBC often experience abnormal thyroid function, with a significantly higher incidence (ranging from 5.6 to 23.6%) than non-PBC patients^1^. There is also a higher risk of developing AITDs for those with PBC^34^. Our study, which utilized a GWAS dataset with a larger sample size of AITD and PBC, showed that PBC greatly contributed to the development of AITD, including its subgroups of AHYPER and AHYPO, while AITD was not identified as a risk factor for the development of PBC, which is consistent with previous research conducted by HUANG^35^. GD and HT are the most prominent disease components of autoimmune hyperthyroidism and autoimmune hypothyroidism, respectively, with Hashimoto thyroiditis being the most common type of PBC. Floreani et al.^34^ reported a prevalence rate as high as 20.4% in PBC patients and 3.2% suffering from Graves' disease. Another study^36^ conducted by a European team found that 16.3% of 921 PBC patients had concomitant thyroid disease, with 10.2% having HT, 1.6% having GD, and 41 having other types of thyroid disease. Of these, 77.4% had thyroid disease concurrent with or immediately following PBC. Our study confirmed it may be related to the promotion of PBC by AHYPO rather than AHYPER, and our use of GWAS data for GD and validation yielded the same results as for AHYPER. Unfortunately, we failed to perform approximate equivalence validation for AHYPO due to the lack of a GWAS dataset for HT in the European population.

IBD is a systemic condition that can have extraintestinal manifestations in 5% to 50% of patients, but comorbid PBC is less commonly reported. It is believed that IBD may be most closely associated with primary sclerosing cholangitis (PSC), which has a prevalence of 0.12% to 10.97% in patients with UC^37^. Compared to PSC, comorbidity between PBC and IBD is relatively rare^38^. Our findings suggest that IBD, together with its subtype CD and UC, contributes to the development of PBC. Meanwhile, PBC contributes genetically to IBD and UC. These results were similar to the previous MR results^9^ as well as conventional observations^38–40^. Compared to Zhang^9^, we conducted a study with larger sample sizes and achieved inconsistent estimates across the four statistical methods used. In contrast to the findings of Zhao et al.^41^, we emphasized the positive promoting effect of PBC on UC onset. This discrepancy might be attributed to the use of larger sample size GWAS data for PBC and the exclusion of loci within the MHC region that could interfere with causal inference.

Observational studies have described a higher prevalence of PBC in UC patients than in non-UC patients and that UC diagnosis often precedes PBC diagnosis. Our results show that the PBC contributes to the UC, rather than a CD, which seems to explain the results of observational studies. Studies have shown that CD and UC have their own specific immune cell populations^42^, and there may be common pathogenic pathways in the development of PBC and CD^36^. Transcriptomic analysis has shown that CD is more closely associated with viral infection and autoimmunity, whereas microbiome-associated immunity may play a more important role in UC^43^. It may indicate that gut microbiota plays a more prominent role in the pathogenesis of PBC and UC, which needs to be verified by more research areas. If impaired liver function in PBC patients can lead to impaired bile secretion and reduced hepatic detoxification capacity, which in turn affects the intestinal flora, dendritic cells may be more abundant in UC than in CD by sensing the intestinal microbiota and initiating an innate immune response that may be more likely to induce UC disease^43,44^, which may be one of the reasons for the greater comorbidity of UC in PBC patients. While subtypes of the same broad disease group may share some similarities in genetic background, significant differences in individual genetics, genetics and immunity remain between CD and UC. Further studies are needed to elucidate the differences between the two subtypes.

Research has shown that up to 7% of patients with PBC also have CeD, and liver abnormalities are often found in conjunction with CeD^45^. In a group of 440 PBC cases, CeD was the most prevalent autoimmune disease affecting the gastrointestinal tract (1.7%)^3^. Another study demonstrated that CeD is more common in PBC patients than in those with other liver diseases^46^. In fact, data collected from a UK registry revealed that 6% of CeD patients also had PBC^47^. However, we analyzed a GWAS dataset with a larger sample size of PBC and discovered a unidirectional positive causal relationship between CeD and PBC, which inconsistent with the research conducted by Li et al.^48^.

There is belief that the connection between PBC and MS is linked to environmental factors affecting those who are genetically vulnerable, and it is uncommon for MS and PBC to co-occur^49^. According to meta-analyses, there may be a significant genetic correlation between MS and PBC^50^. Our research indicates that patients with PBC have a higher chance of developing MS, while patients with MS do not have a higher risk of developing PBC. A study conducted across multiple centers and based on population did not reveal an increased risk of autoimmune disease in MS patients^51^, which aligns with the outcomes of our MR study.

Research has shown that psoriasis occurs more frequently in patients with PBC, with a prevalence of up to 6%, compared to the general population, but there is a lack of studies on larger PBC groups^52,53^. Our study indicates that PBC has a positive impact on psoriasis in one direction. This is consistent with the findings of Zhao et al.^54^, despite using different datasets for PBC and PoS, which enhances the reliability of our results. As the onset of PBC is gradual and can be masked during psoriasis treatment, it is crucial for psoriasis patients to distinguish between drug-induced liver enzyme abnormalities and overlapping PBC disease^55^.

The connection between PBC and EHAID comorbidity has been a topic of discussion, as their pathogenesis is believed to be related to a common genetic and autoimmune basis. However, there are differences in their pathogenetic features, clinical manifestations and disease interactions that are not fully understood. To address this issue, we conducted an MR study based on the principle of random assignment of alleles using GWAS data from a large number of PBC and EHAID cases by eliminating MHC loci confounding variables, which is a limitation of observational studies. We used multiple MR statistical methods to improve the reliability of our findings, providing new theoretical support for the study of PBC and EHAIDs. However, our study has some limitations, including the low number of GWAS cases for certain EHAIDs (e.g., SSc, AHYPER), which may affect the causal relationship with PBC. In addition, all confounding factors associated with outcomes could not be completely excluded, and we could only exclude them at the level of MR Egger's intercept test and MR PRESSO's Global Test using statistical pleiotropy methods to ensure the robustness of the results. Moreover, due to limitations in the available data, the study was confined to European populations, and more data is needed to validate its applicability to other ethnic groups. Lastly, the summary data we used lacked detailed demographic information, which limited our ability to conduct stratified analyses and understand disease dynamics in different populations.

In summary, the current MR analysis provides genetic evidence for a causal relationship between PBC and EHAIDs, i.e., PBC is an active contributor to the above intriguing EHAIDs except for CD and CeD, and in turn, RA, SLE, SS, SSc, IBD, CD, UC, AHYPO and CeD become drivers of PBC pathogenesis. This underscores the significance of prioritizing PBC in clinical practice. Additionally, if any liver function abnormalities are observed during treatment or with EHAIDs, it is crucial to consider the possibility of comorbid PBC.

### Supplementary Information

Supplementary Legends.

Supplementary Information 1.

Supplementary Information 2.

Supplementary Information 3.

Supplementary Information 4.

Supplementary Information 5.

Supplementary Information 6.

## Data Availability

All GWAS data analysed during this study are from IEU open gwas project (https://gwas.mrcieu.ac.uk/), GWAS Catalog (https://www.ebi.ac.uk/gwas/), and FinnGen consortium (https://www.finngen.fi/fi) and shown in Table 1.
