# Supplementary material for: Dissecting causal relationships between primary biliary cholangitis and extrahepatic autoimmune diseases based on Mendelian randomization
Source: Sci Rep. 2024 May 21;14:11528. doi: 10.1038/s41598-024-62509-x (PMC11109240; doi:10.1038/s41598-024-62509-x)
Supplement: Supplementary file 1 — Supplementary Legends. [file 41598_2024_62509_MOESM1_ESM.docx]

**Supplementary legends**

**Supplementary 1.** Sensitivity Analysis for Mendelian Randomization.

**Supplementary 2.** Instruments Variables for Mendelian Randomization.

**Supplementary 3.** Scatter Plots for Mendelian randomization.

**Supplementary 4.** Forest Plots for Mendelian randomization.

**Supplementary 5.** Funnel Plots for Mendelian randomization.

**Supplementary 6.** Leave-one-out Plots for Mendelian randomization
