# Supplementary material for: Dissecting causal relationships between primary biliary cholangitis and extrahepatic autoimmune diseases based on Mendelian randomization
Source: Sci Rep. 2024 May 21;14:11528. doi: 10.1038/s41598-024-62509-x (PMC11109240; doi:10.1038/s41598-024-62509-x)
Supplement: Supplementary file 4 — Supplementary Information 3. [file 41598_2024_62509_MOESM4_ESM.pdf]

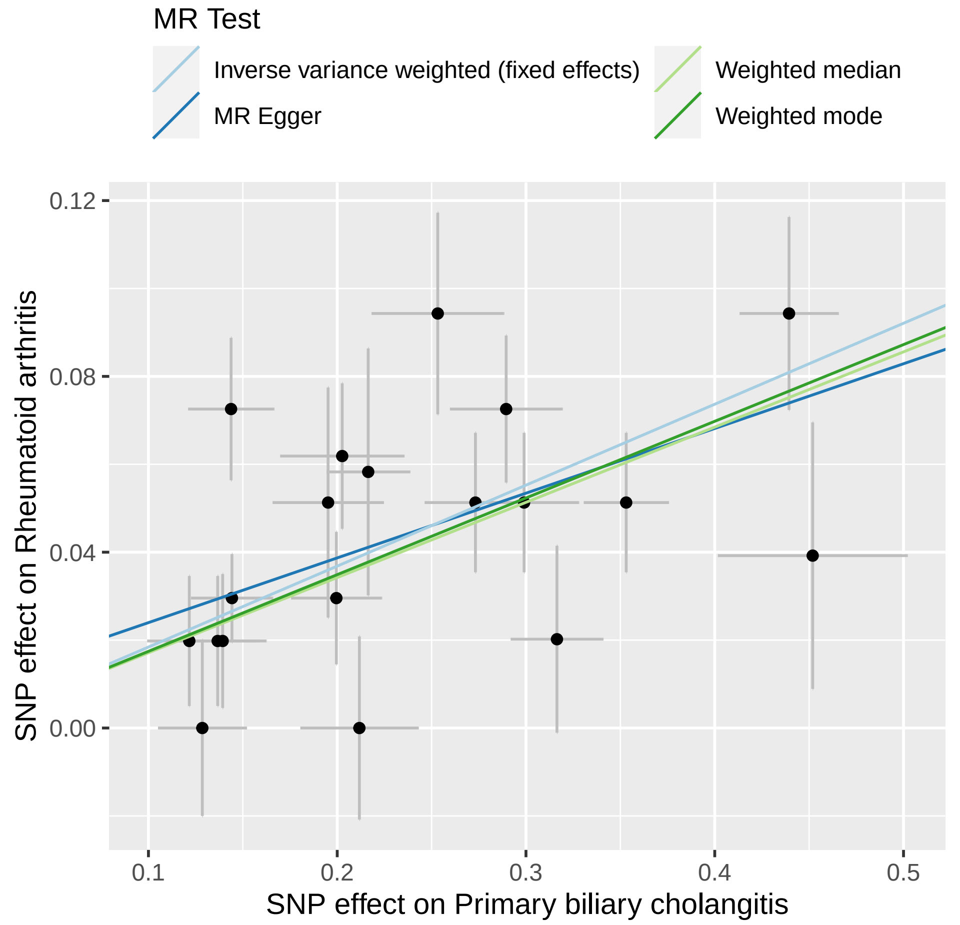
MR Test
Inverse variance weighted (fixed effects)
MR Egger
Weighted median
Weighted mode
0.12
0.08
0.04
0.00
0.1
0.2
0.3
0.4
0.5
SNP effect on Rheumatoid arthritis
SNP effect on Primary biliary cholangitis

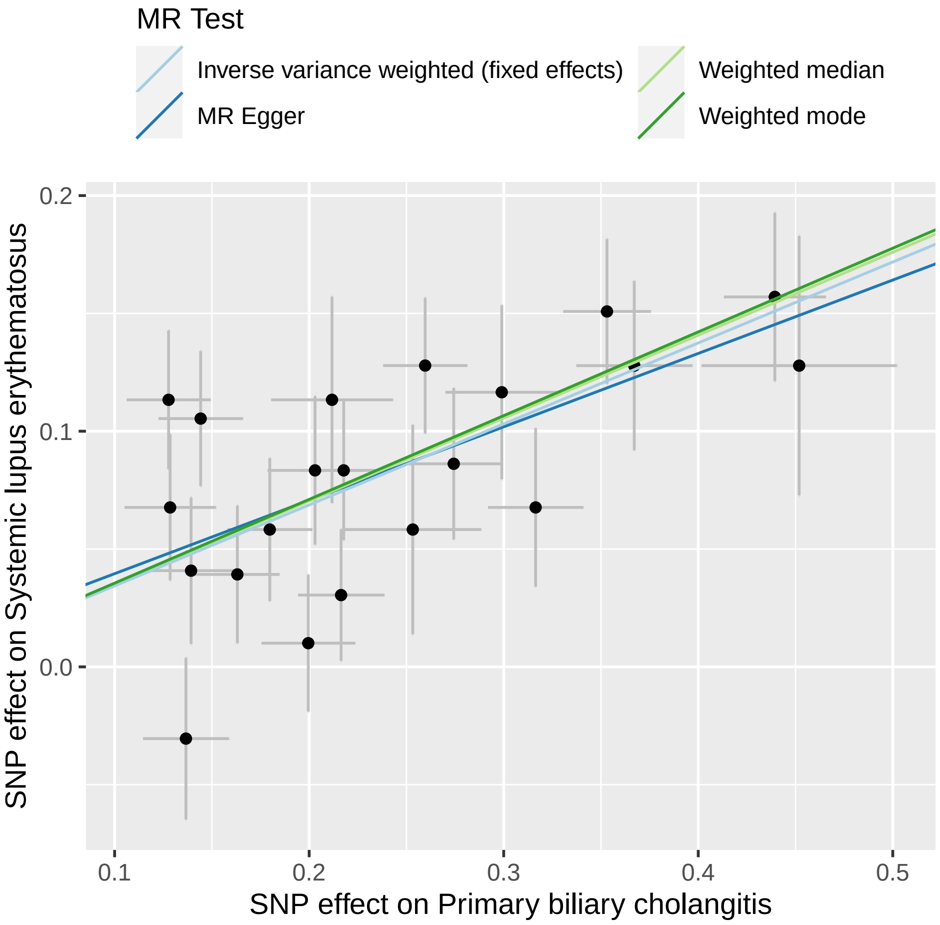
MR Test
Inverse variance weighted (fixed effects)
MR Egger
Weighted median
Weighted mode
0.2
0.1
0.0
0.1
0.2
0.3
0.4
0.5
SNP effect on Systemic lupus erythematosus
SNP effect on Primary biliary cholangitis

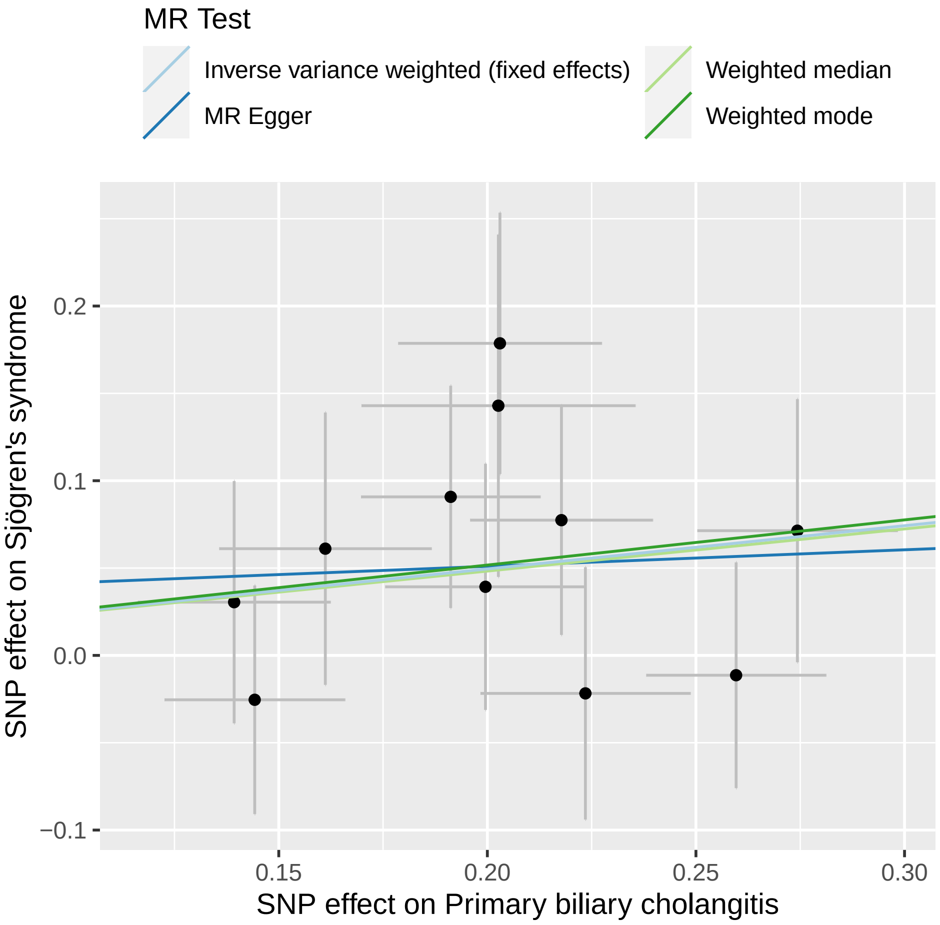
MR Test
Inverse variance weighted (fixed effects)
Weighted median
MR Egger
Weighted mode
0.2
0.1
0.0
−0.1
0.15
0.20
0.25
0.30
SNP effect on Sjögren's syndrome
SNP effect on Primary biliary cholangitis

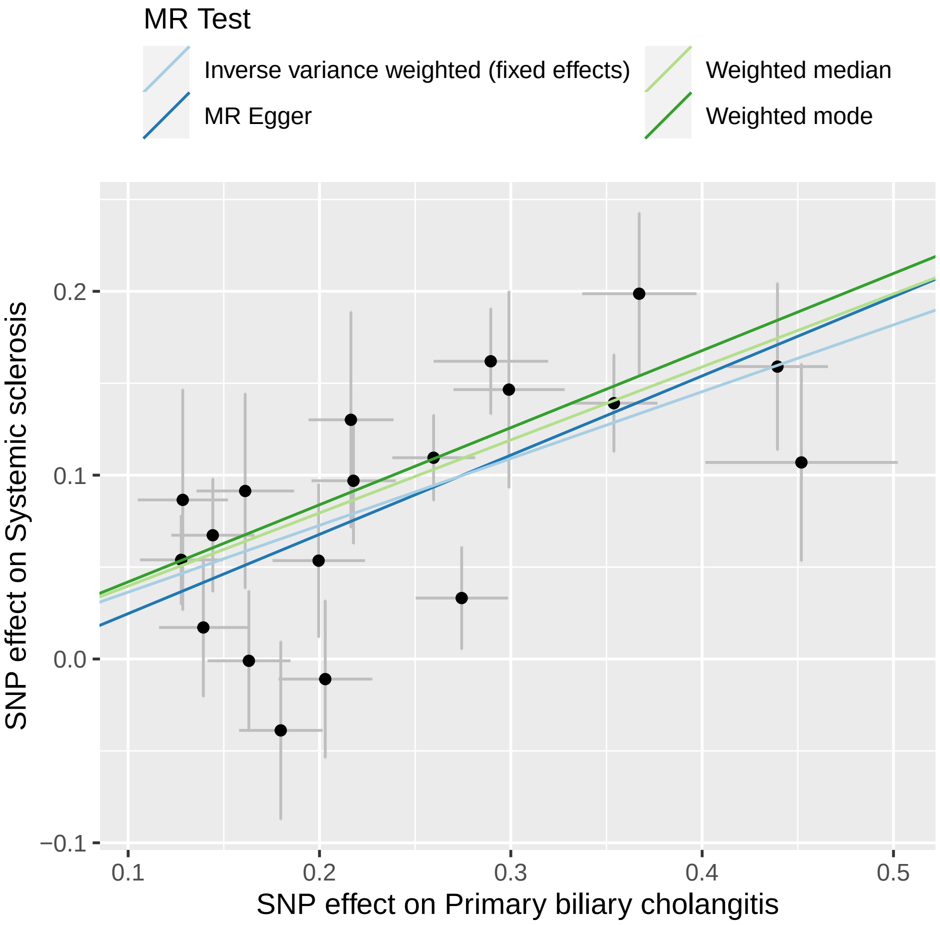

MR Test
Inverse variance weighted (fixed effects)
Weighted median
MR Egger
Weighted mode
0.2
0.1
0.0
−0.1
SNP effect on Systemic sclerosis
0.1
0.2
0.3
0.4
0.5
SNP effect on Primary biliary cholangitis

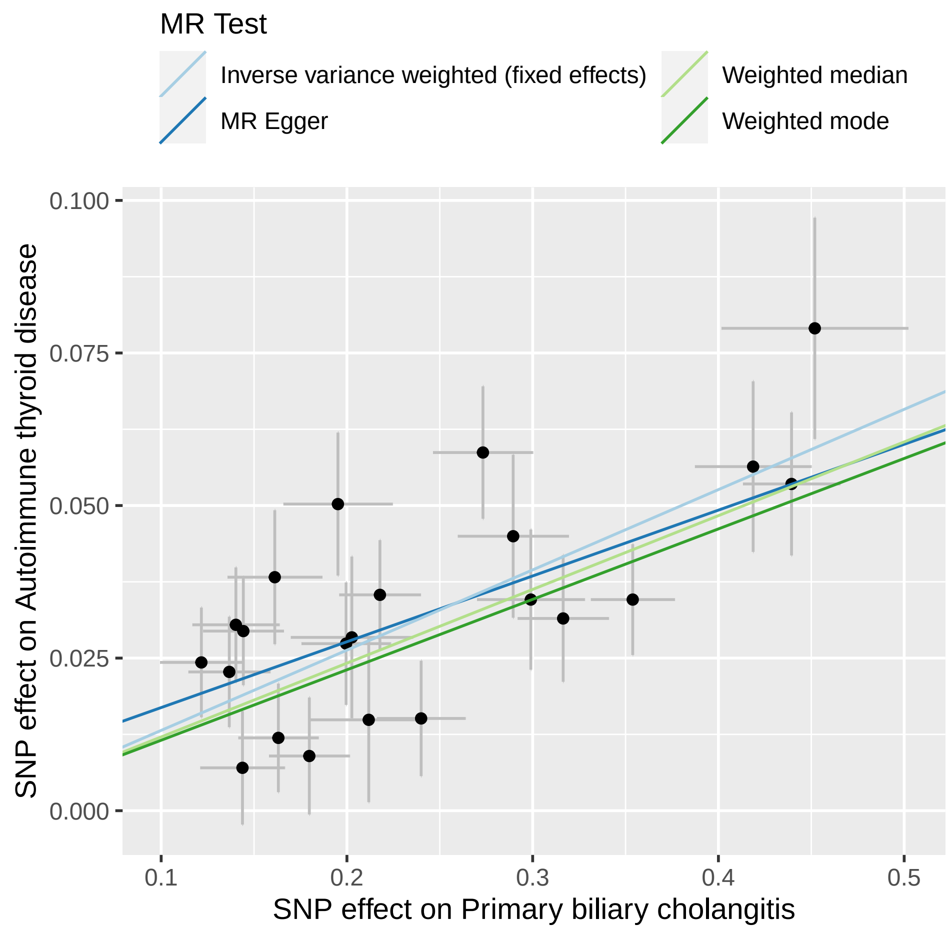

MR Test
Inverse variance weighted (fixed effects)
MR Egger
Weighted median
Weighted mode
0.100
0.075
0.050
0.025
0.000
0.1
0.2
0.3
0.4
0.5
SNP effect on Autoimmune thyroid disease
SNP effect on Primary biliary cholangitis

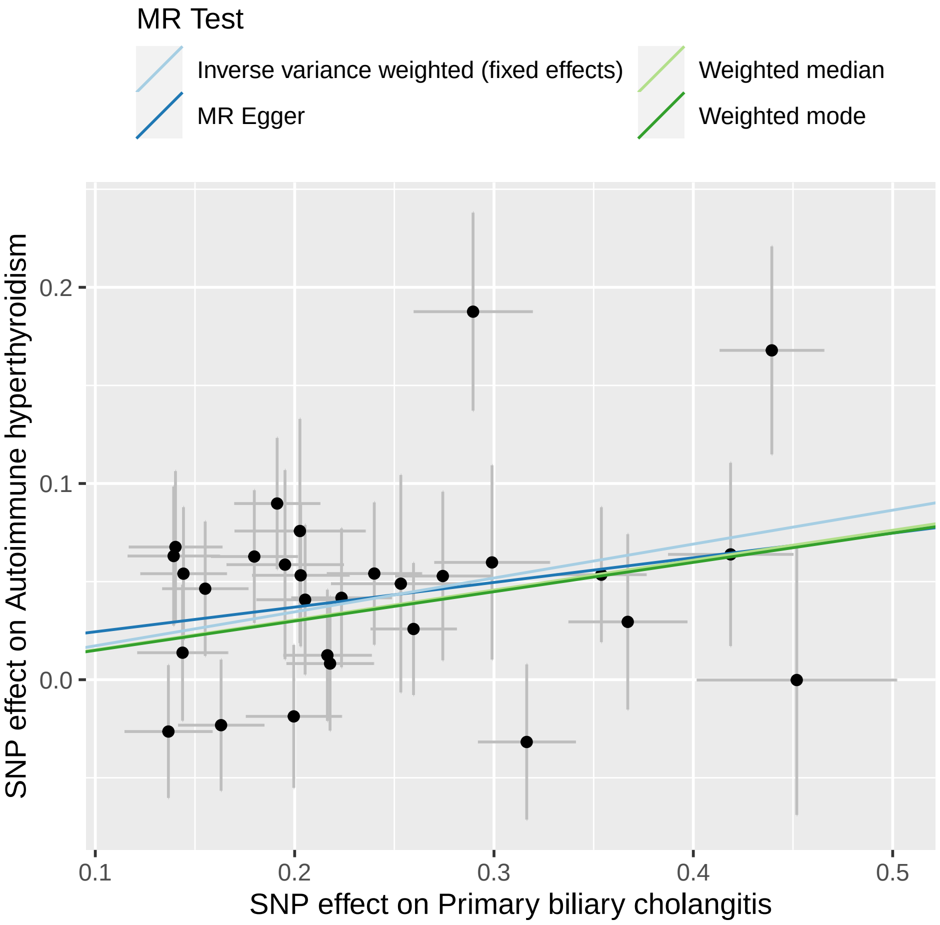

MR Test
Inverse variance weighted (fixed effects)
MR Egger
Weighted median
Weighted mode
0.2
0.1
0.0
SNP effect on Autoimmune hyperthyroidism
0.1
0.2
0.3
0.4
0.5
SNP effect on Primary biliary cholangitis

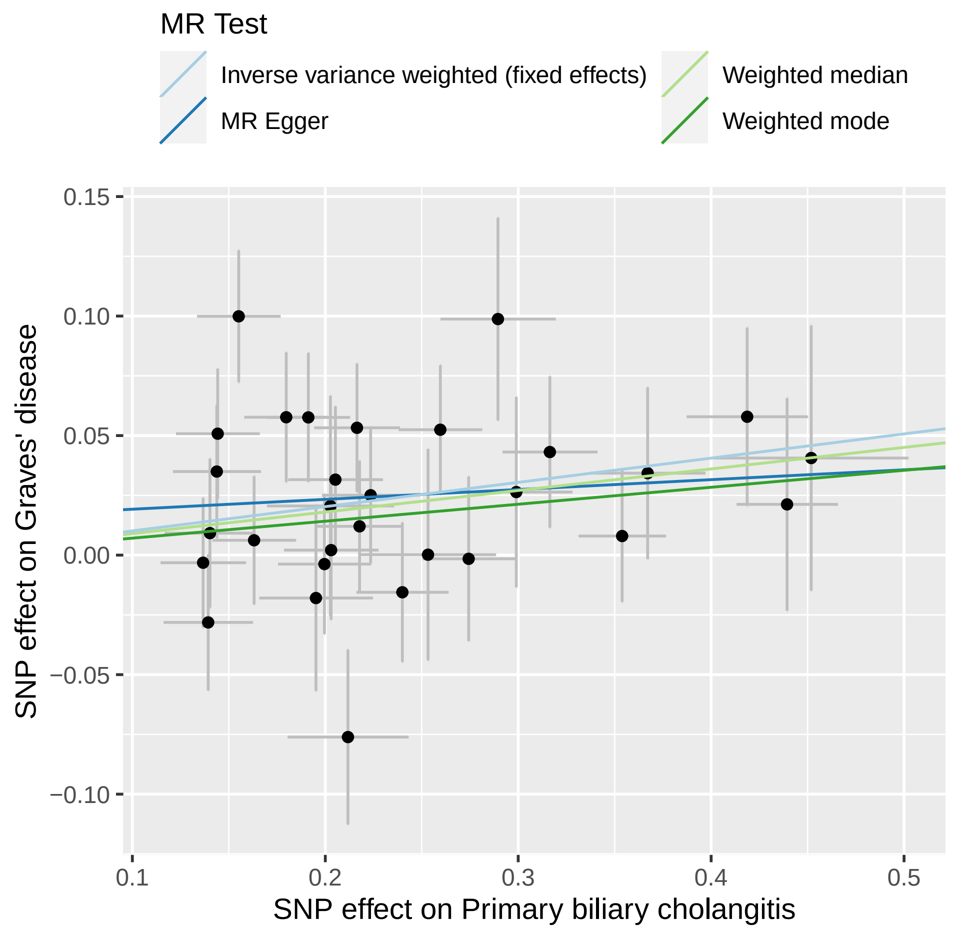
MR Test
Inverse variance weighted (fixed effects)
MR Egger
Weighted median
Weighted mode
0.15
0.10
0.05
0.00
−0.05
−0.10
SNP effect on Graves' disease
0.1
0.2
0.3
0.4
0.5
SNP effect on Primary biliary cholangitis

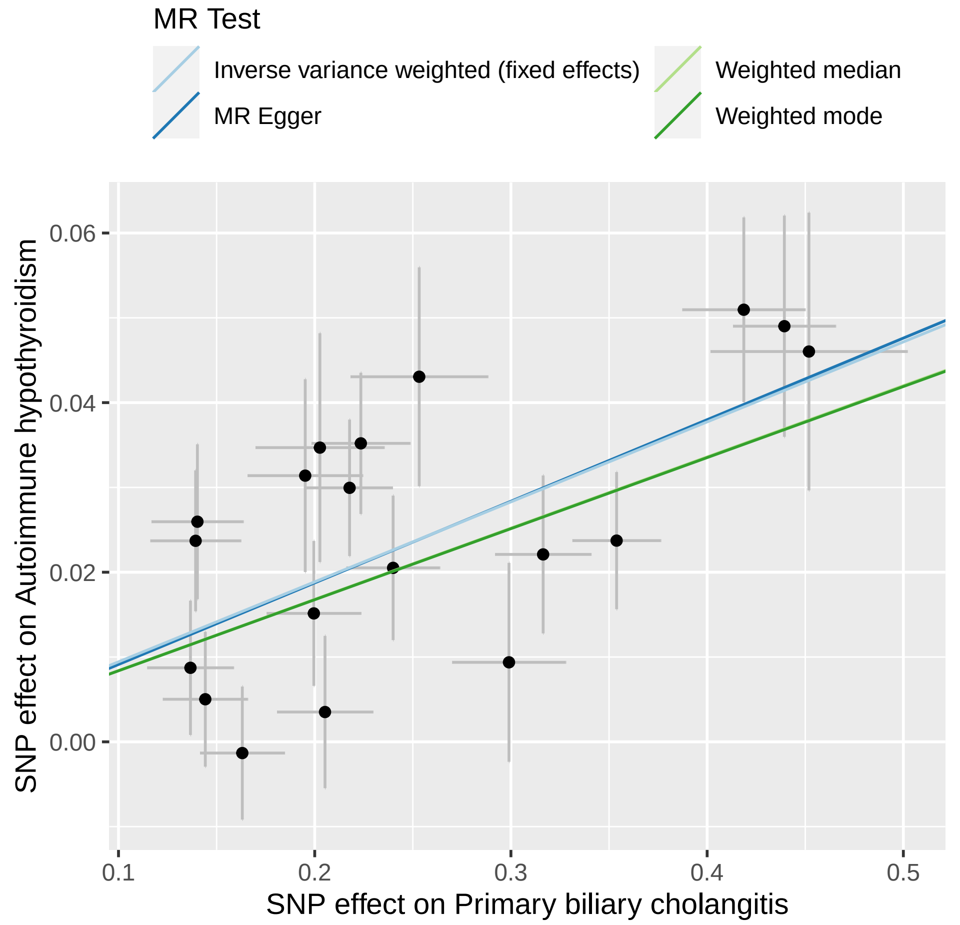
MR Test
Inverse variance weighted (fixed effects)
MR Egger
Weighted median
Weighted mode
0.06
0.04
0.02
0.00
0.1
0.2
0.3
0.4
0.5
SNP effect on Autoimmune hypothyroidism
SNP effect on Primary biliary cholangitis

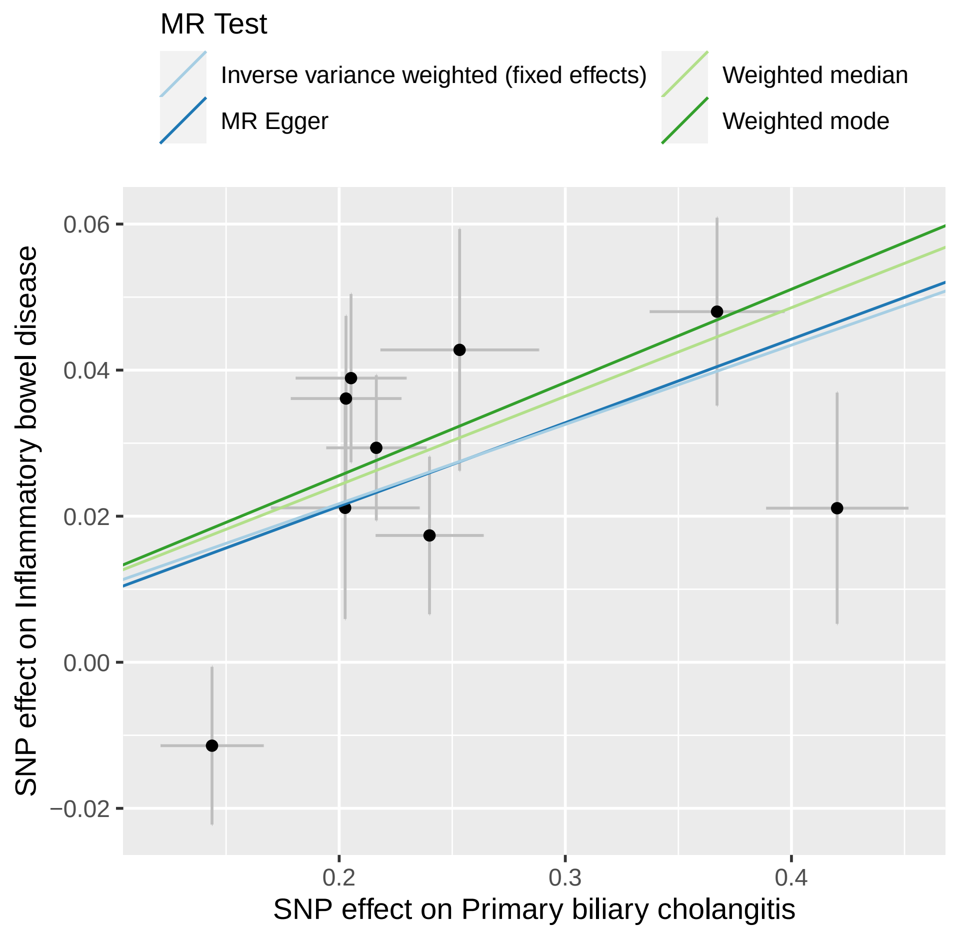
MR Test
Inverse variance weighted (fixed effects)
MR Egger
Weighted median
Weighted mode
SNP effect on Inflammatory bowel disease
0.06
0.04
0.02
0.00
−0.02
0.2
0.3
0.4
SNP effect on Primary biliary cholangitis

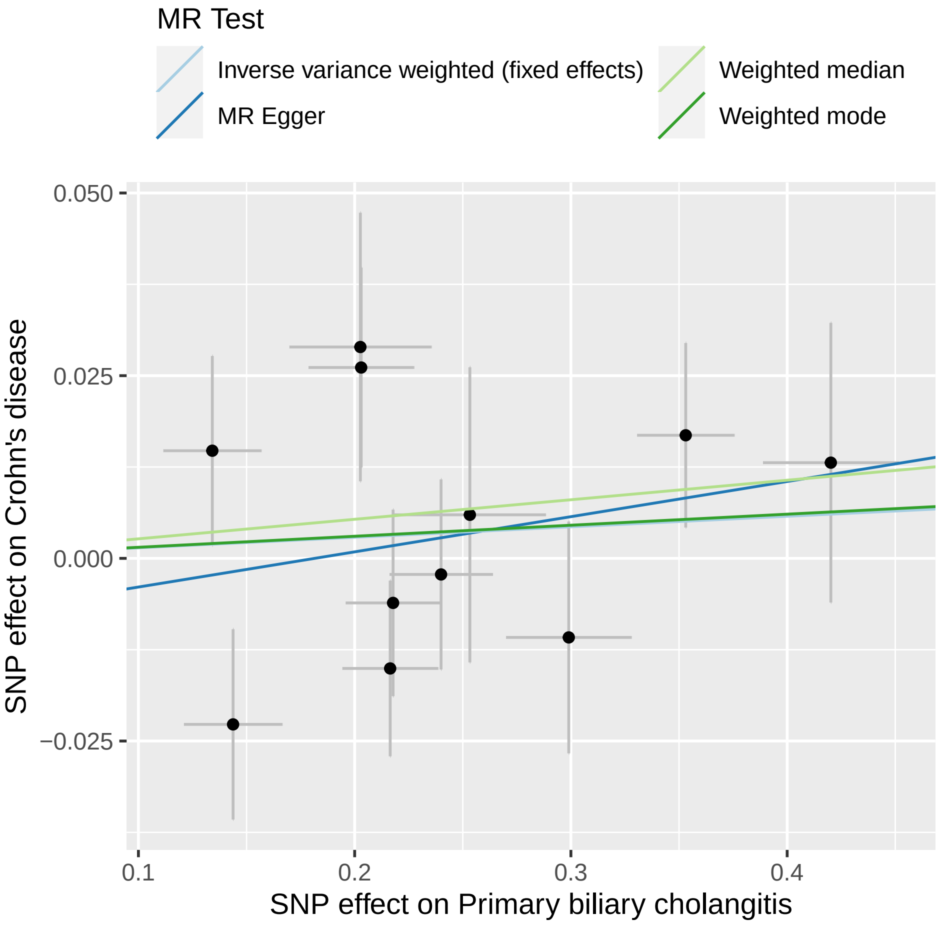

MR Test
Inverse variance weighted (fixed effects)
MR Egger
Weighted median
Weighted mode
0.050
0.025
0.000
−0.025
0.1
0.2
0.3
0.4
SNP effect on Crohn's disease
SNP effect on Primary biliary cholangitis

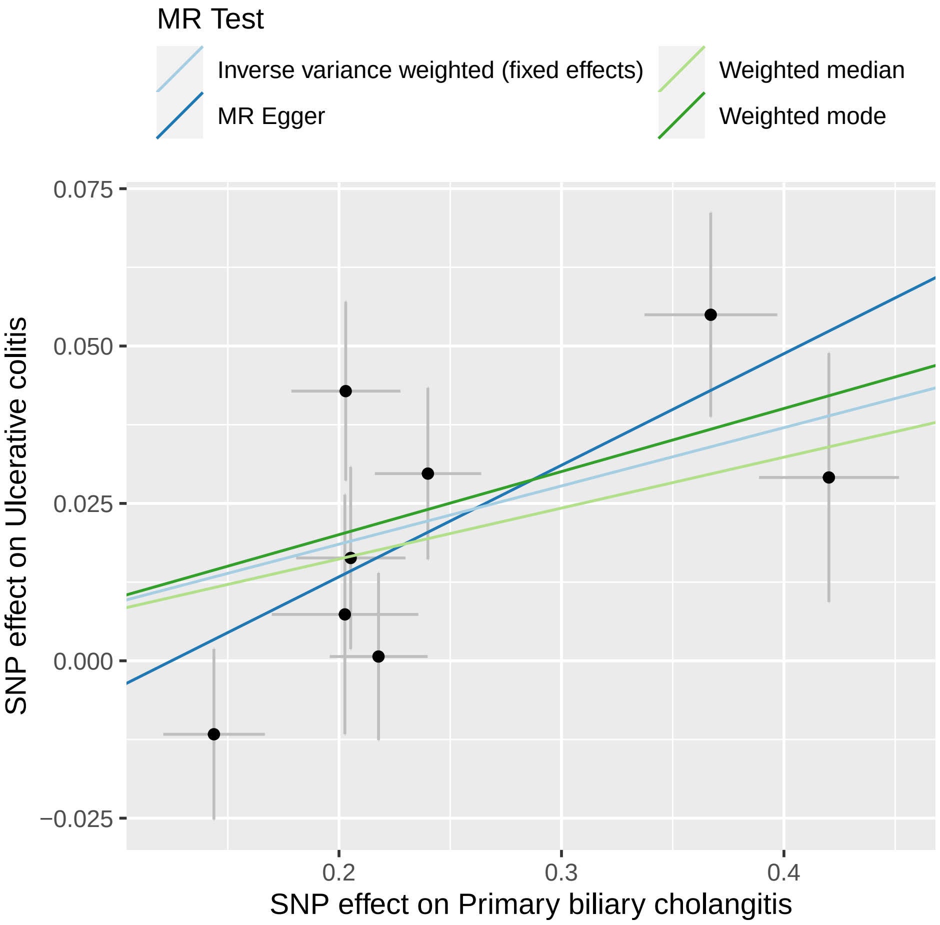
MR Test
Inverse variance weighted (fixed effects)
MR Egger
Weighted median
Weighted mode
0.075
0.050
0.025
0.000
−0.025
0.2
0.3
0.4
SNP effect on Ulcerative colitis
SNP effect on Primary biliary cholangitis

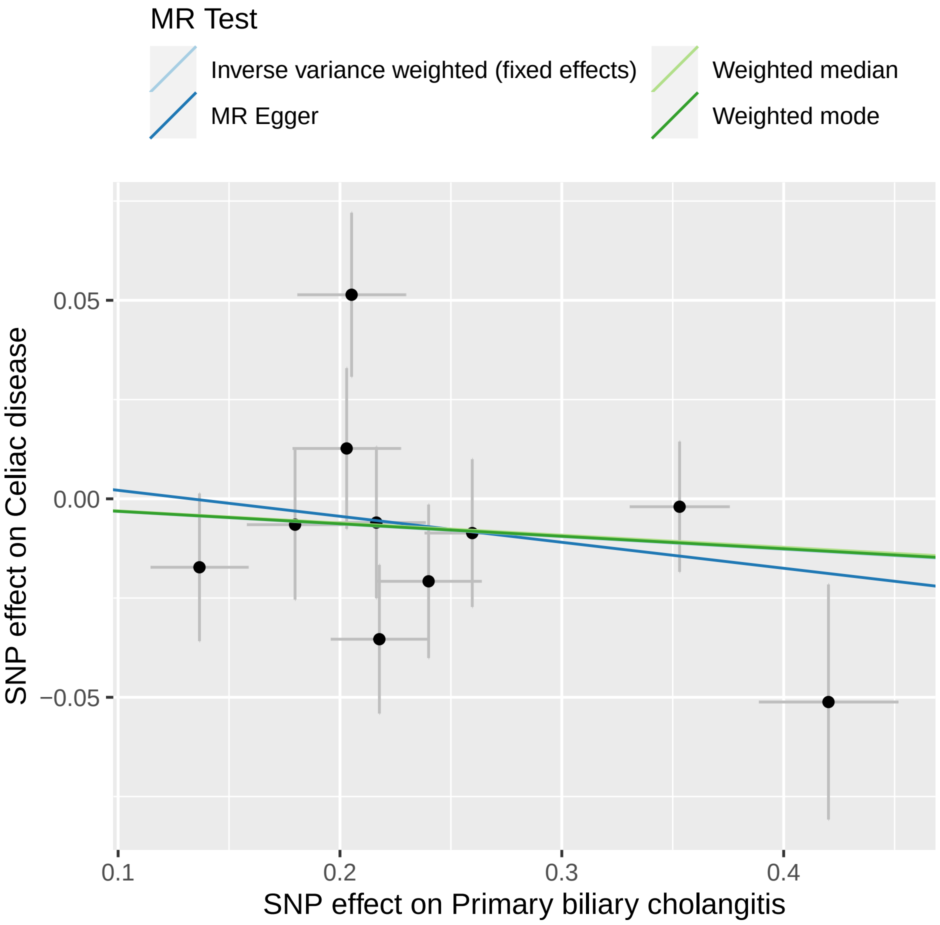
MR Test
Inverse variance weighted (fixed effects)
MR Egger
Weighted median
Weighted mode
0.05
0.00
−0.05
0.1
0.2
0.3
0.4
SNP effect on Celiac disease
SNP effect on Primary biliary cholangitis

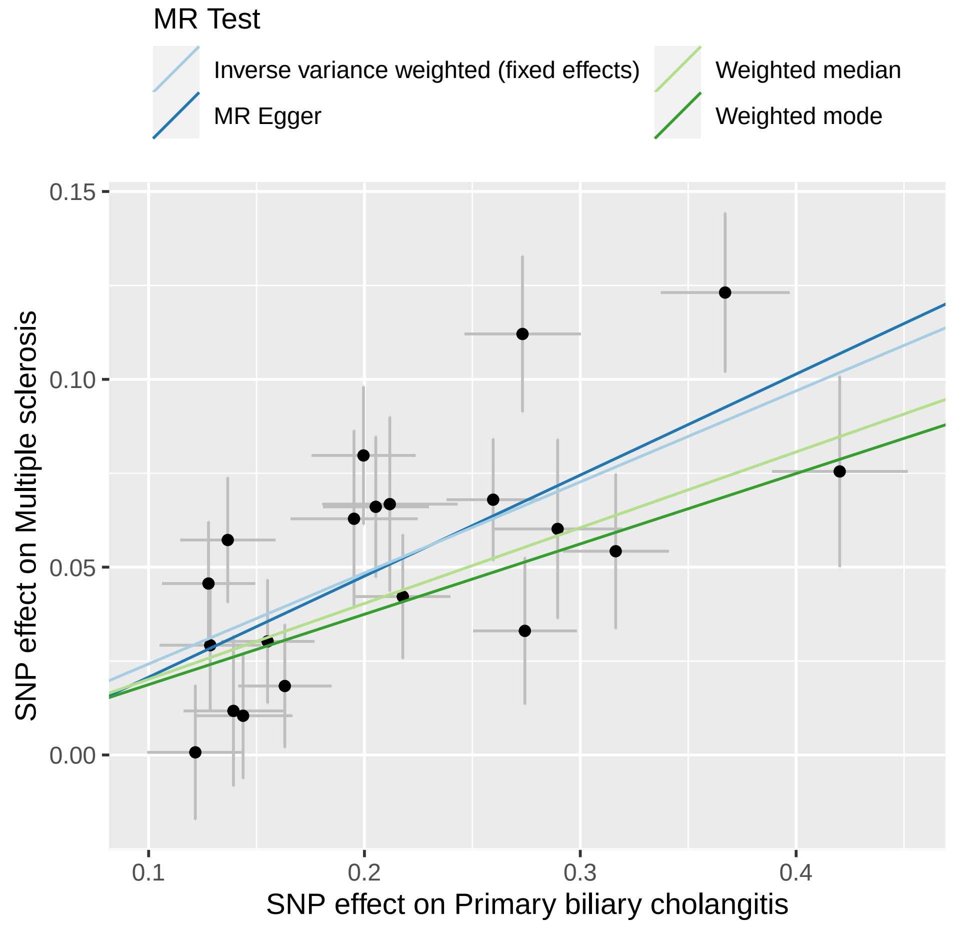
MR Test
Inverse variance weighted (fixed effects)
MR Egger
Weighted median
Weighted mode
0.15
0.10
0.05
0.00
0.1
0.2
0.3
0.4
SNP effect on Multiple sclerosis
SNP effect on Primary biliary cholangitis

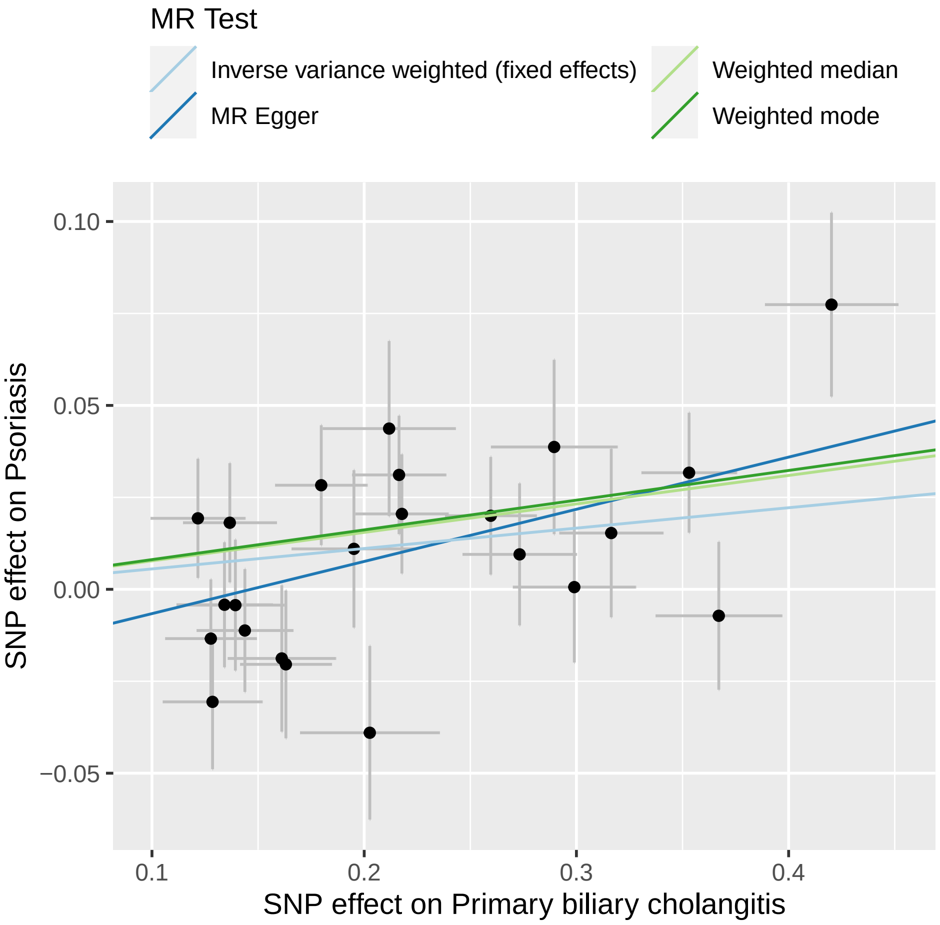
MR Test
Inverse variance weighted (fixed effects)
MR Egger
Weighted median
Weighted mode
0.10
0.05
0.00
−0.05
0.1
0.2
0.3
0.4
SNP effect on Psoriasis
SNP effect on Primary biliary cholangitis

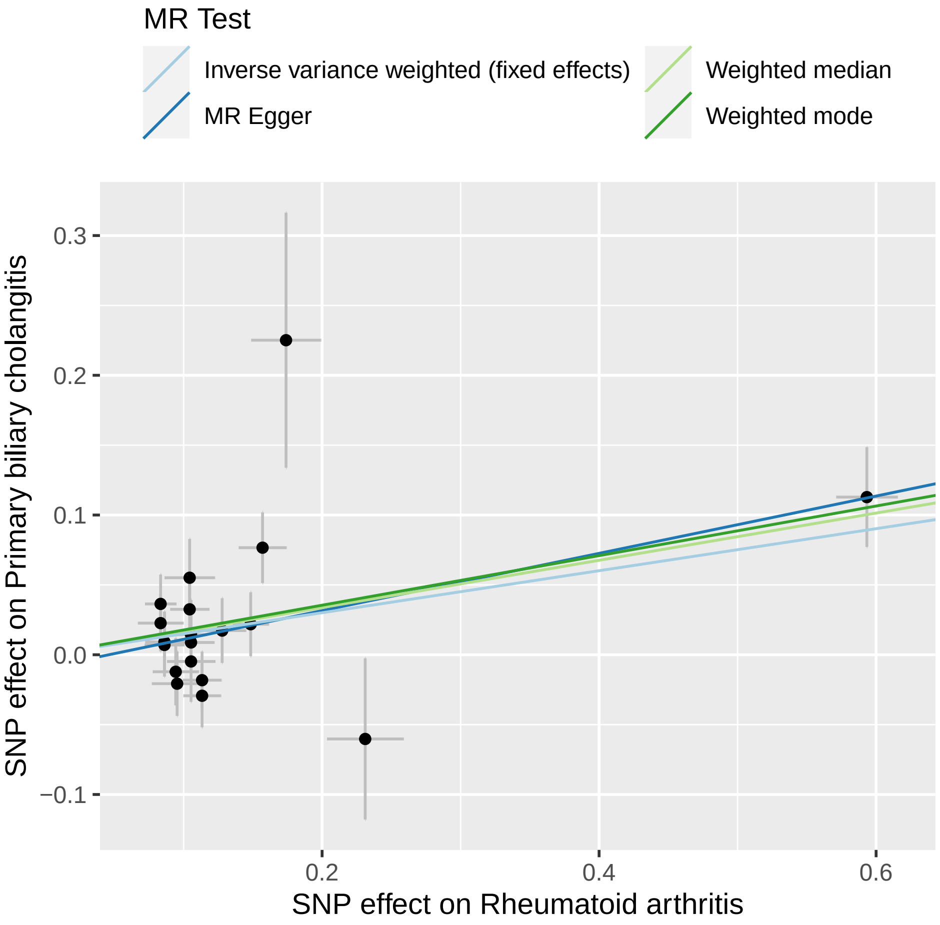

MR Test
Inverse variance weighted (fixed effects)
MR Egger
Weighted median
Weighted mode
0.3
0.2
0.1
0.0
−0.1
0.2
0.4
0.6
SNP effect on Primary biliary cholangitis
SNP effect on Rheumatoid arthritis

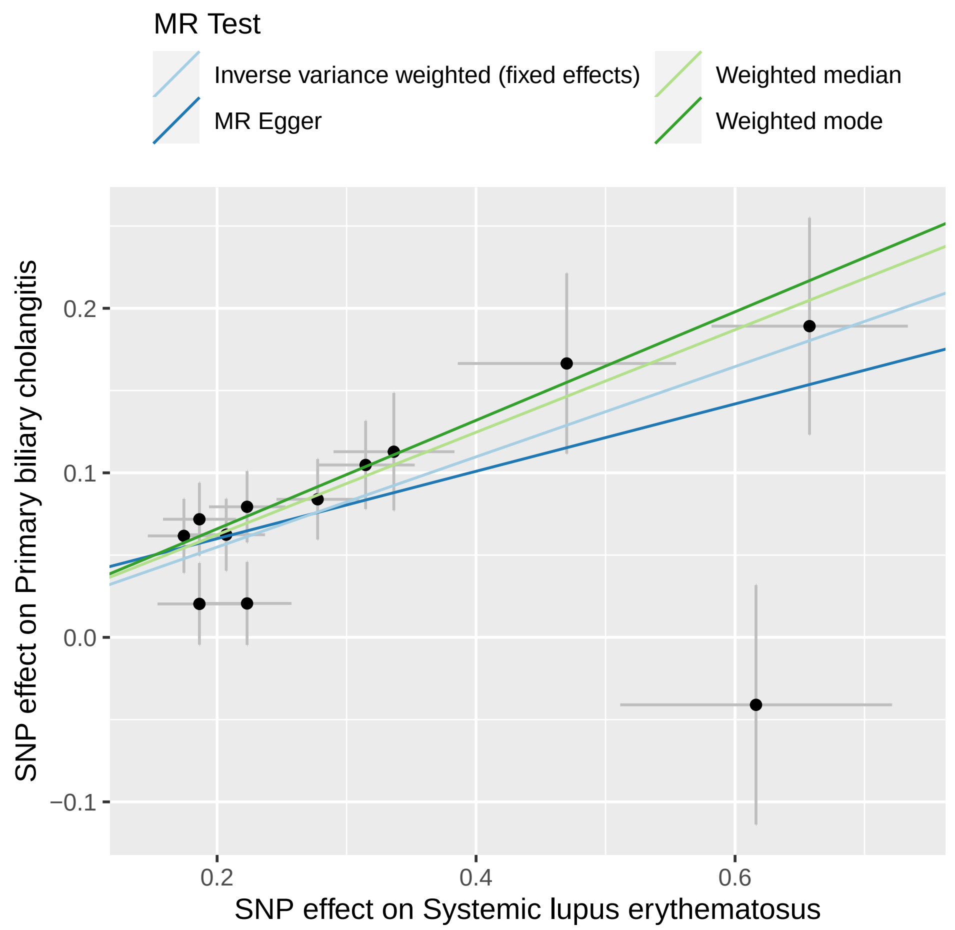

MR Test
Inverse variance weighted (fixed effects)
MR Egger
Weighted median
Weighted mode
0.2
0.1
0.0
−0.1
SNP effect on Primary biliary cholangitis
0.2
0.4
0.6
SNP effect on Systemic lupus erythematosus

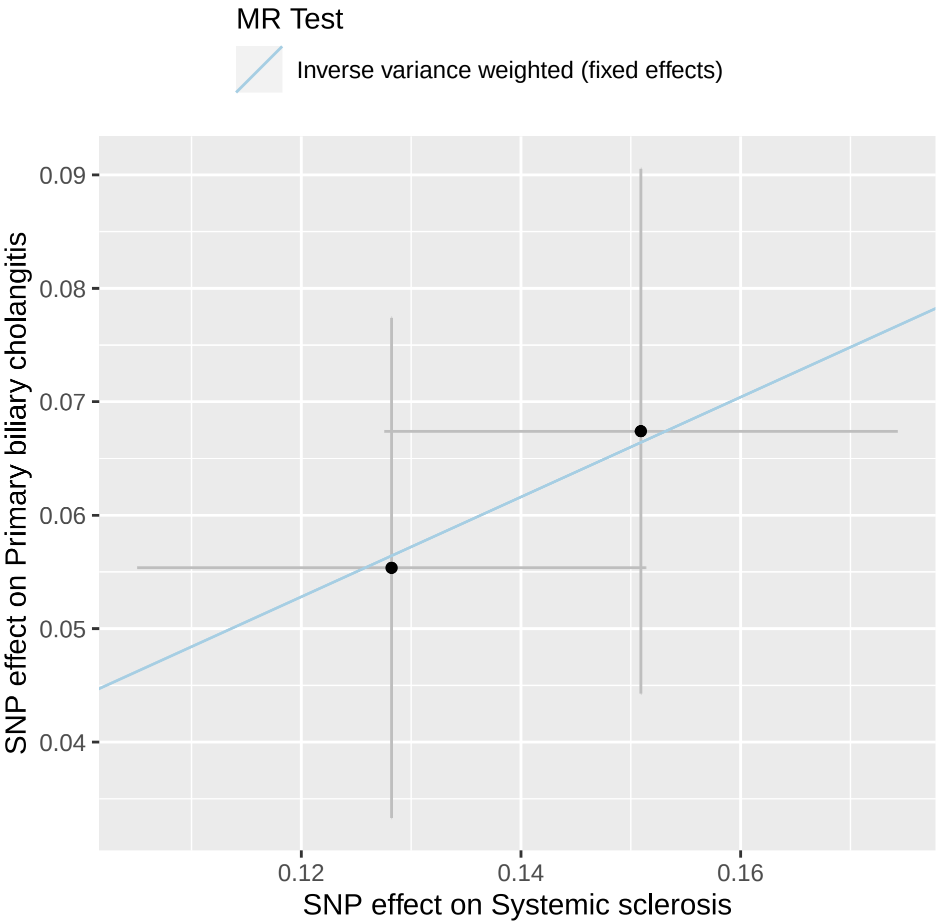
MR Test
Inverse variance weighted (fixed effects)
0.09
0.08
0.07
0.06
0.05
0.04
0.12
0.14
0.16
SNP effect on Primary biliary cholangitis
SNP effect on Systemic sclerosis

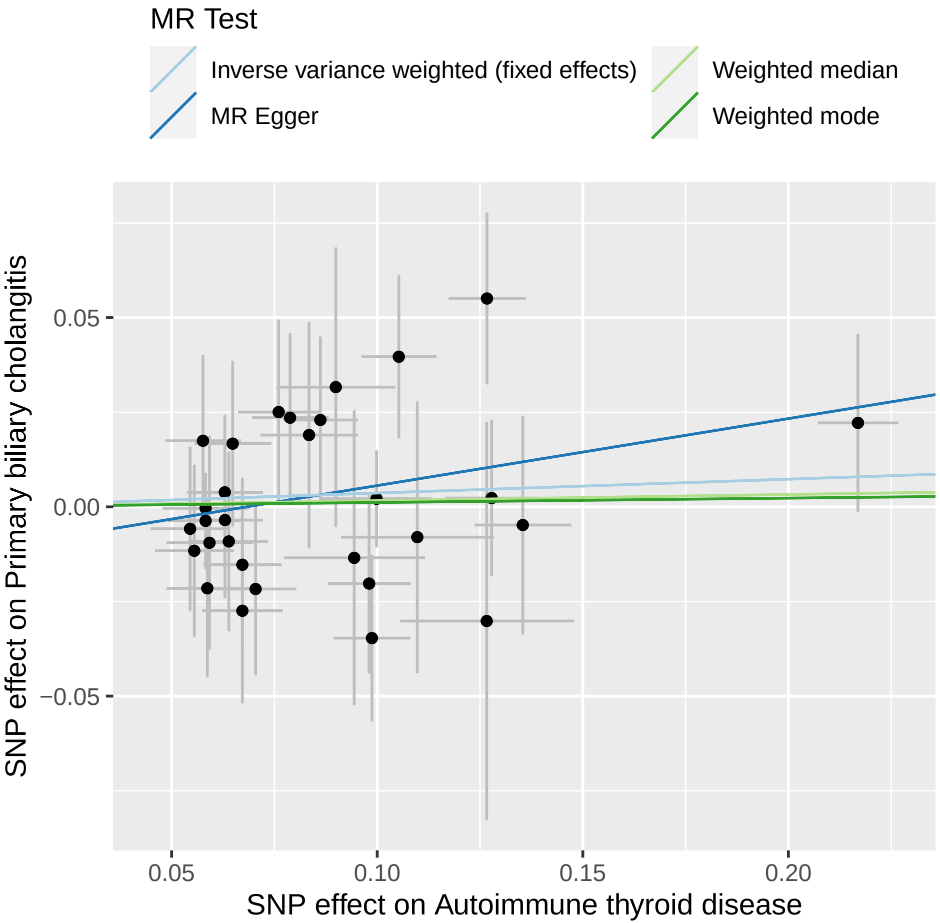
MR Test
Inverse variance weighted (fixed effects)
MR Egger
Weighted median
Weighted mode
0.05
0.00
−0.05
0.05
0.10
0.15
0.20
SNP effect on Primary biliary cholangitis
SNP effect on Autoimmune thyroid disease

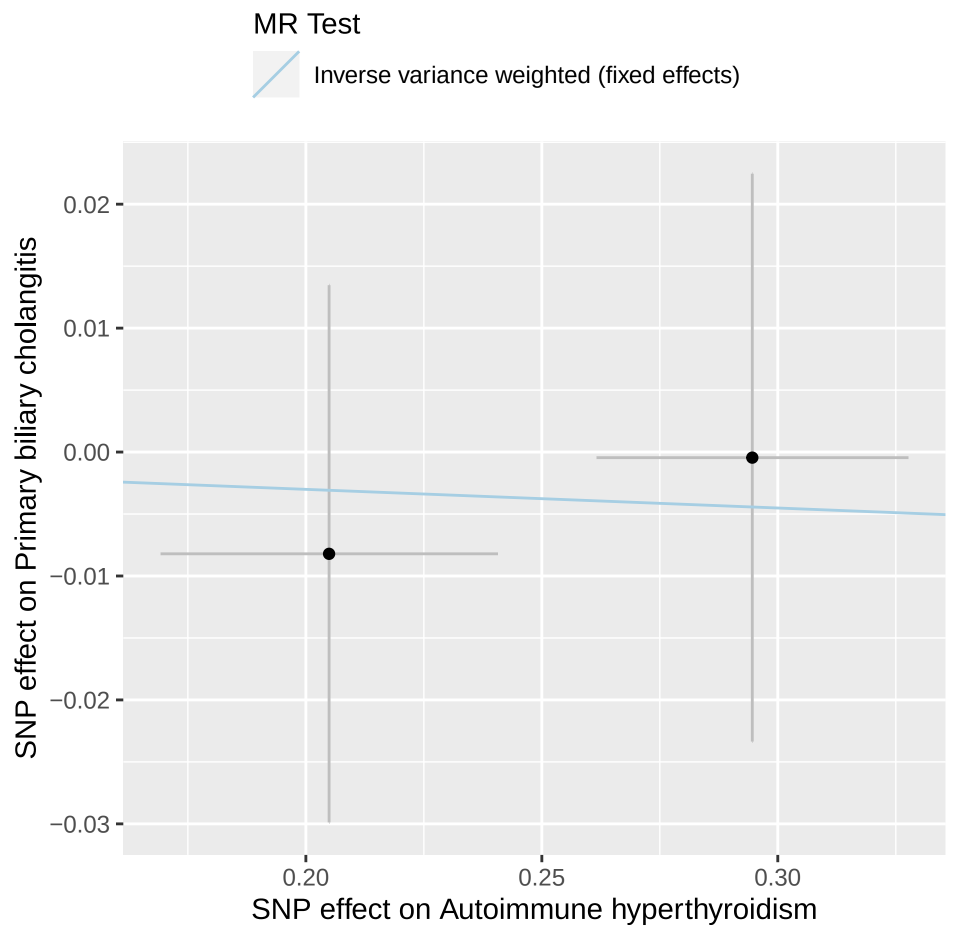
MR Test
Inverse variance weighted (fixed effects)
0.02
0.01
0.00
−0.01
−0.02
−0.03
0.20
0.25
0.30
SNP effect on Primary biliary cholangitis
SNP effect on Autoimmune hyperthyroidism

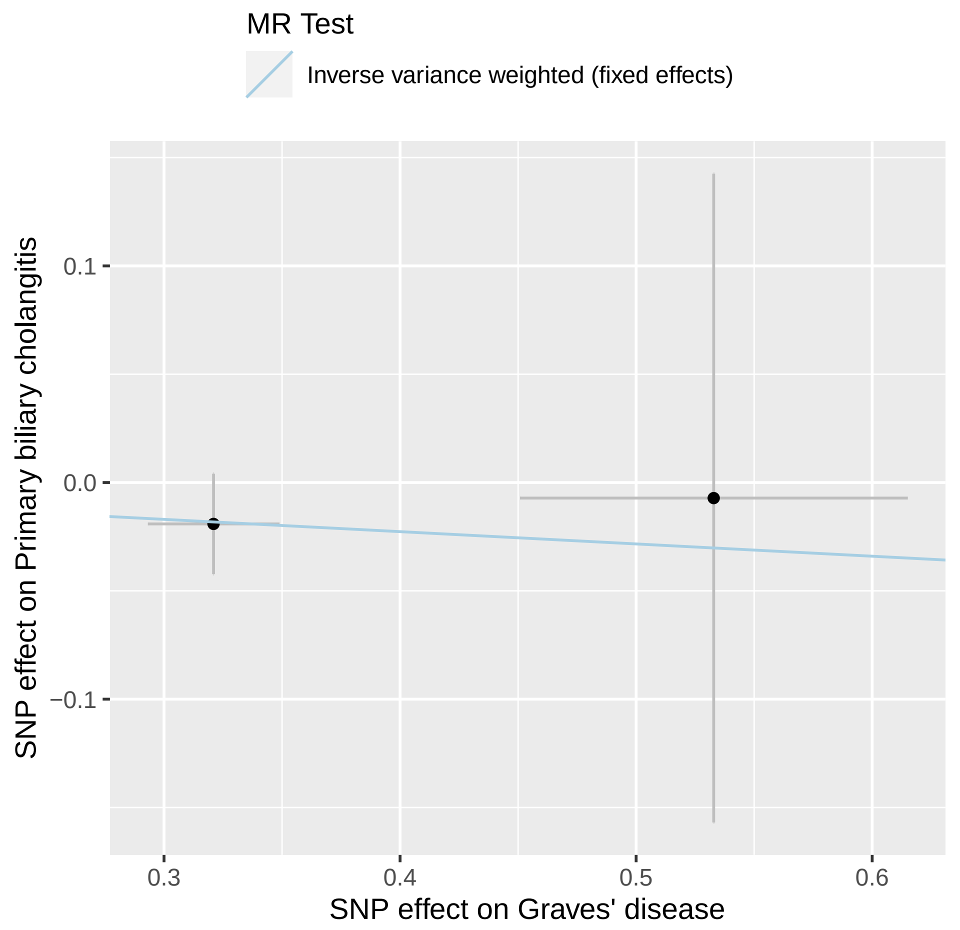
MR Test
Inverse variance weighted (fixed effects)
SNP effect on Primary biliary cholangitis
0.1
0.0
−0.1
0.3
0.4
0.5
0.6
SNP effect on Graves' disease

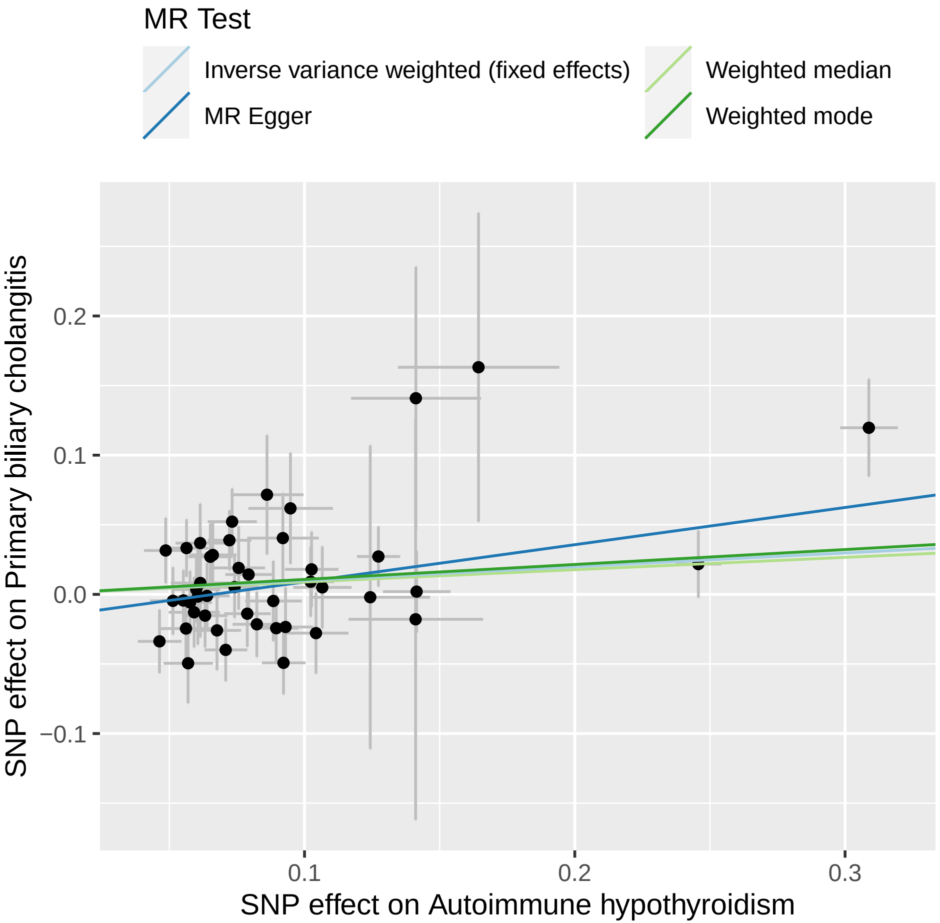
MR Test
Inverse variance weighted (fixed effects)
MR Egger
Weighted median
Weighted mode
SNP effect on Primary biliary cholangitis
0.2
0.1
0.0
−0.1
0.1
0.2
0.3
SNP effect on Autoimmune hypothyroidism

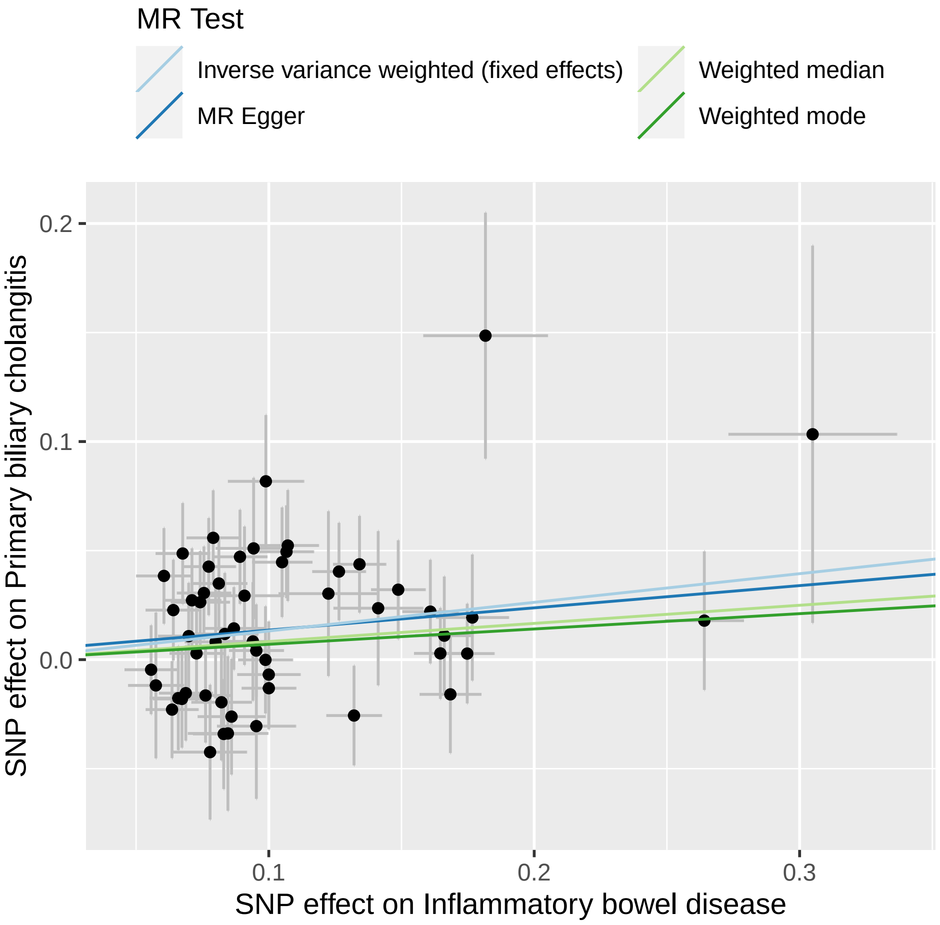
MR Test
Inverse variance weighted (fixed effects)
MR Egger
Weighted median
Weighted mode
0.2
0.1
0.0
0.1
0.2
0.3
SNP effect on Primary biliary cholangitis
SNP effect on Inflammatory bowel disease

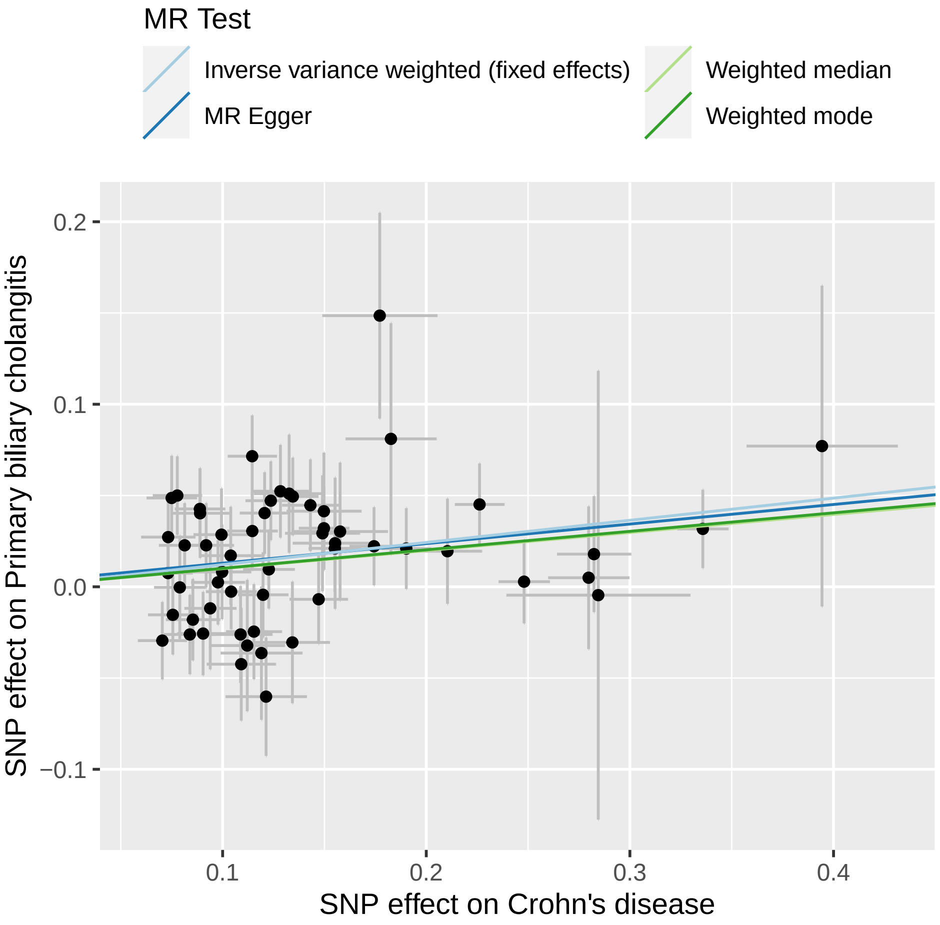
MR Test
Inverse variance weighted (fixed effects)
MR Egger
Weighted median
Weighted mode
SNP effect on Primary biliary cholangitis
0.2
0.1
0.0
−0.1
0.1
0.2
0.3
0.4
SNP effect on Crohn's disease

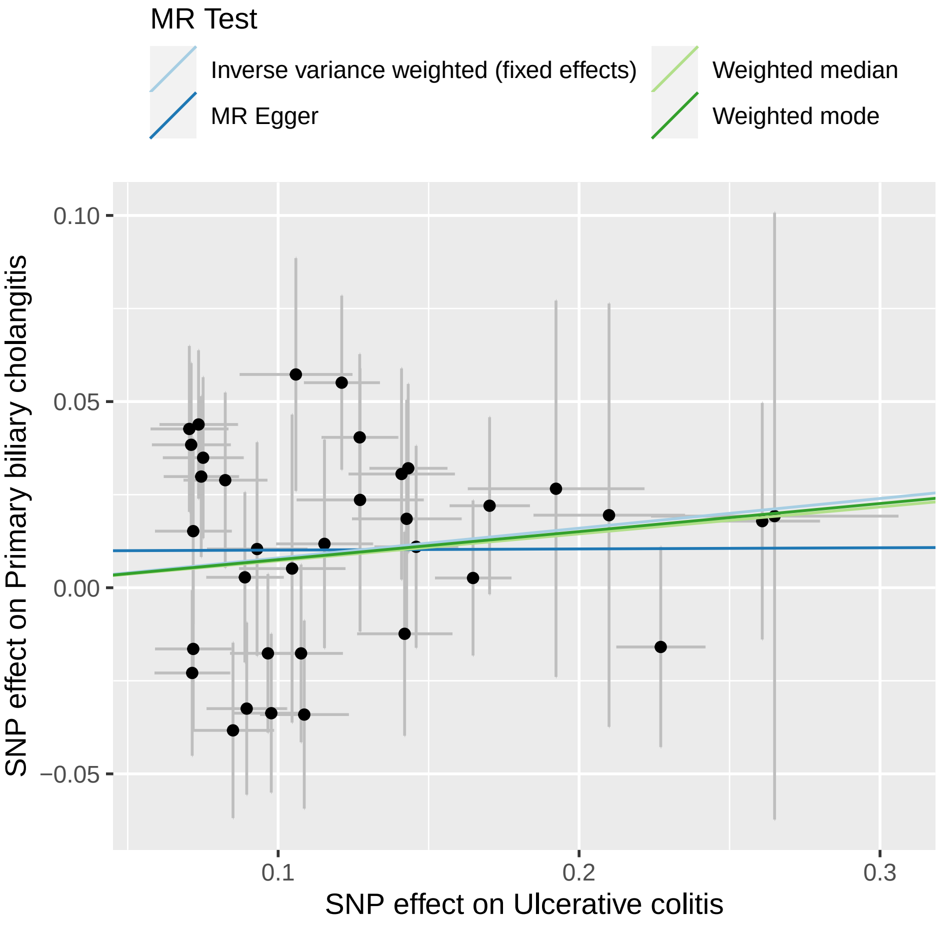
MR Test
Inverse variance weighted (fixed effects)
MR Egger
Weighted median
Weighted mode
0.10
0.05
0.00
−0.05
0.1
0.2
0.3
SNP effect on Primary biliary cholangitis
SNP effect on Ulcerative colitis

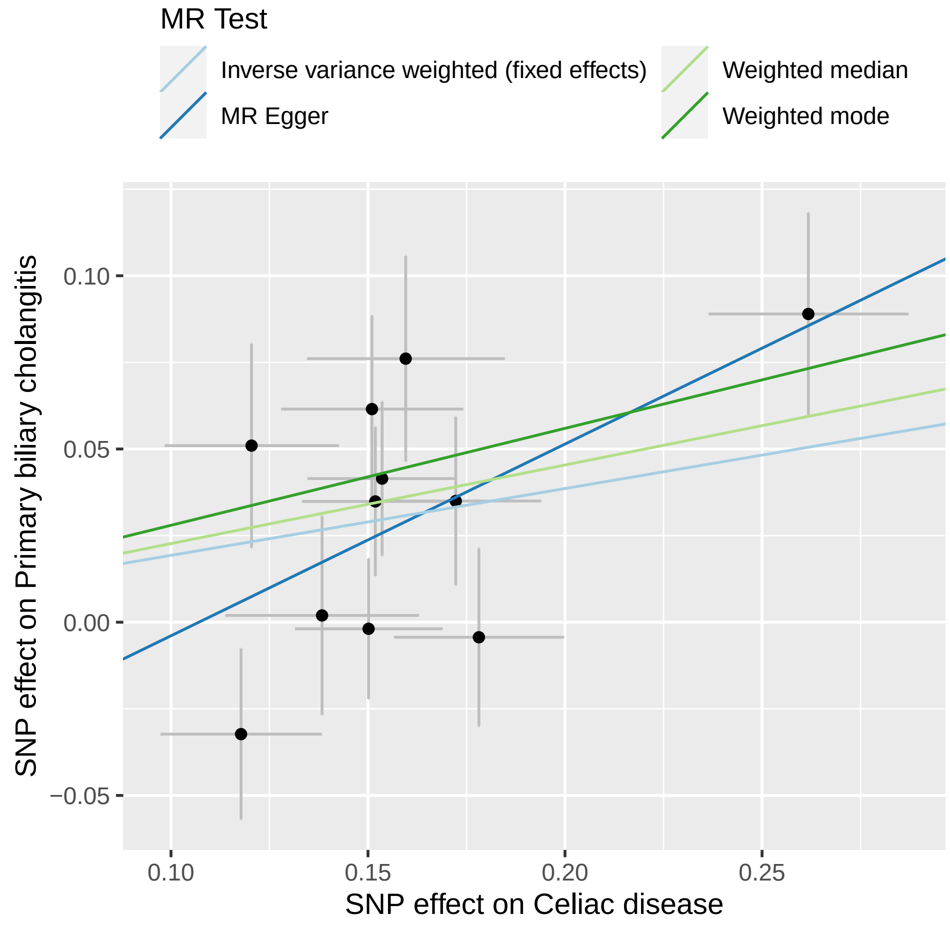
MR Test
Inverse variance weighted (fixed effects)
MR Egger
Weighted median
Weighted mode
SNP effect on Primary biliary cholangitis
0.10
0.05
0.00
−0.05
0.10
0.15
0.20
0.25
SNP effect on Celiac disease

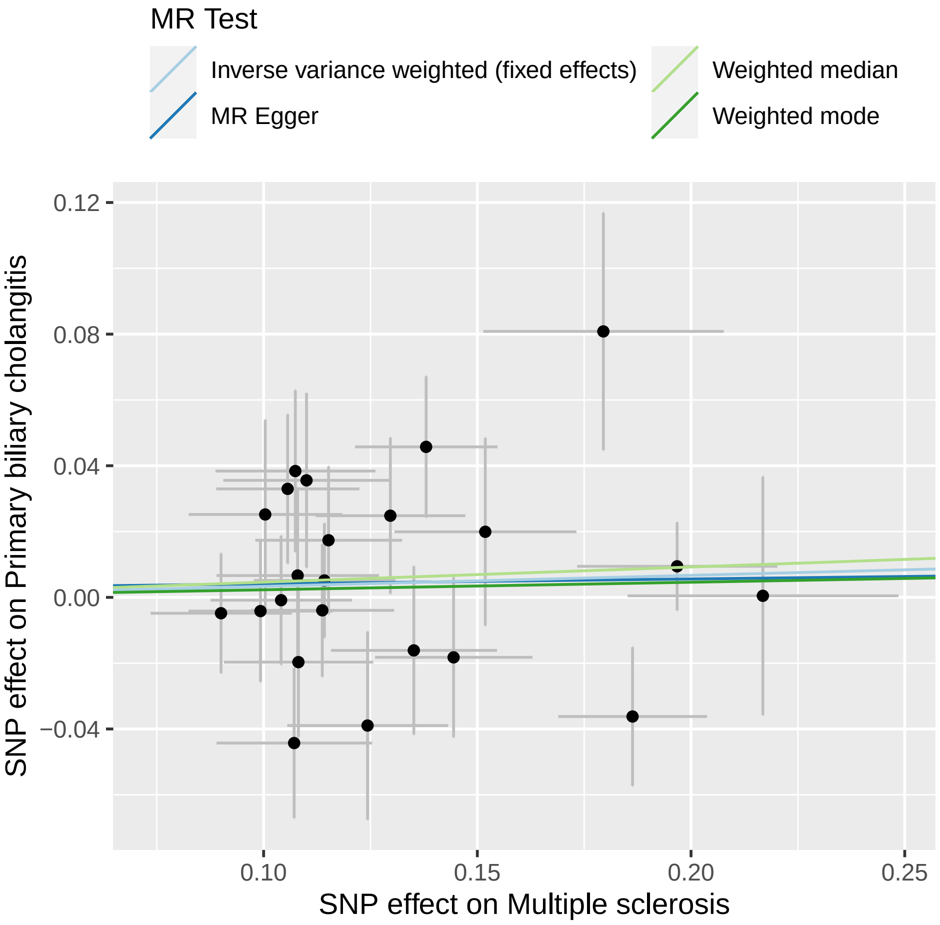
MR Test
Inverse variance weighted (fixed effects)
MR Egger
Weighted median
Weighted mode
0.12
0.08
0.04
0.00
−0.04
0.10
0.15
0.20
0.25
SNP effect on Primary biliary cholangitis
SNP effect on Multiple sclerosis

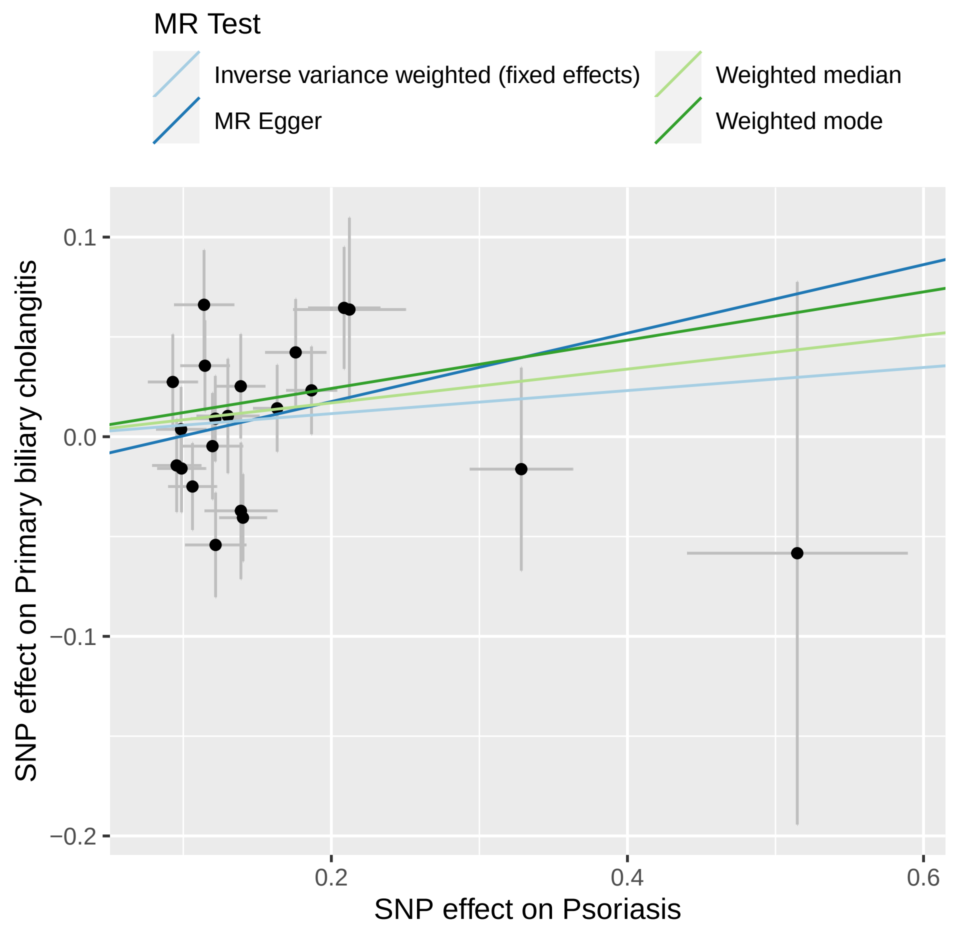
MR Test
Inverse variance weighted (fixed effects)
MR Egger
Weighted median
Weighted mode
0.1
0.0
−0.1
−0.2
SNP effect on Primary biliary cholangitis
0.2
0.4
0.6
SNP effect on Psoriasis
