# Supplementary figures and images for: Dissecting causal relationships between primary biliary cholangitis and extrahepatic autoimmune diseases based on Mendelian randomization
Source: Sci Rep. 2024 May 21;14:11528. doi: 10.1038/s41598-024-62509-x (PMC11109240; doi:10.1038/s41598-024-62509-x)

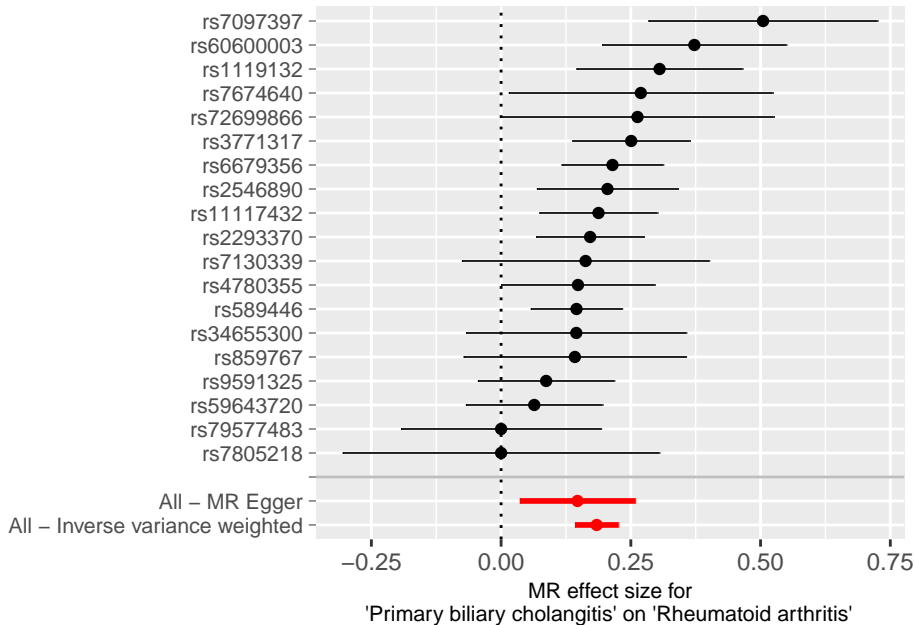

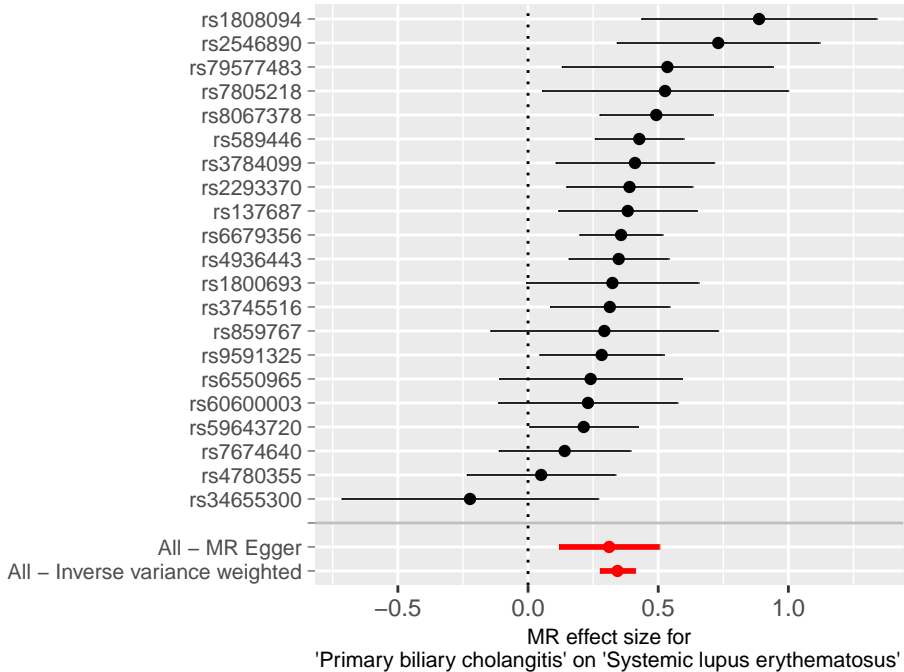

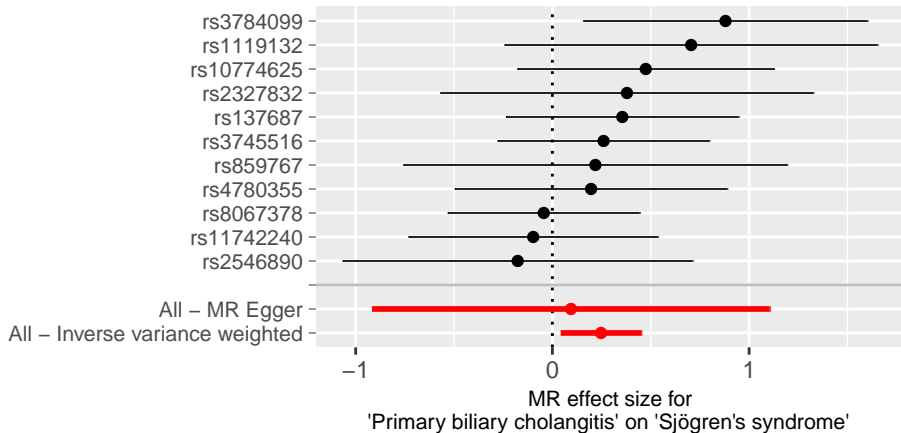

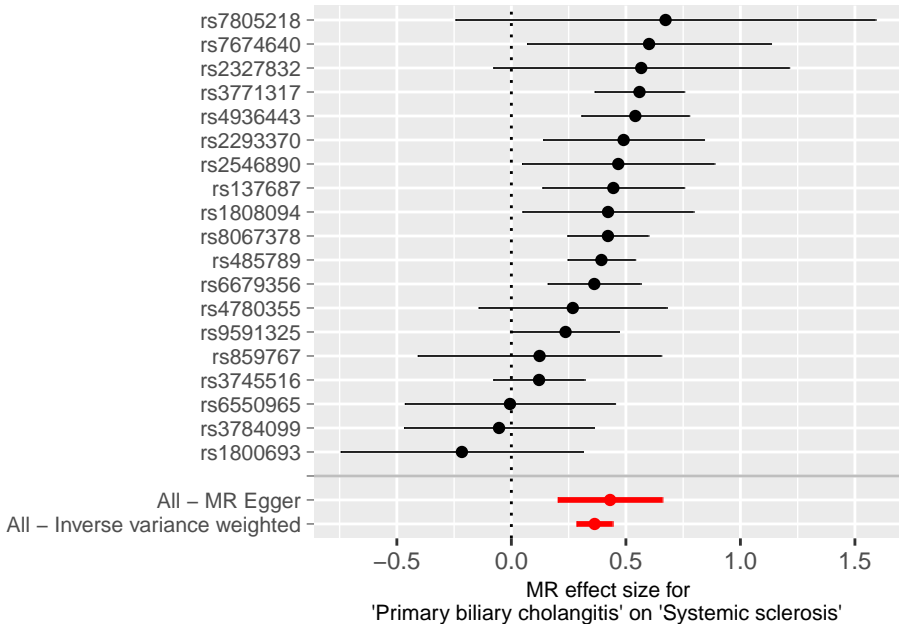

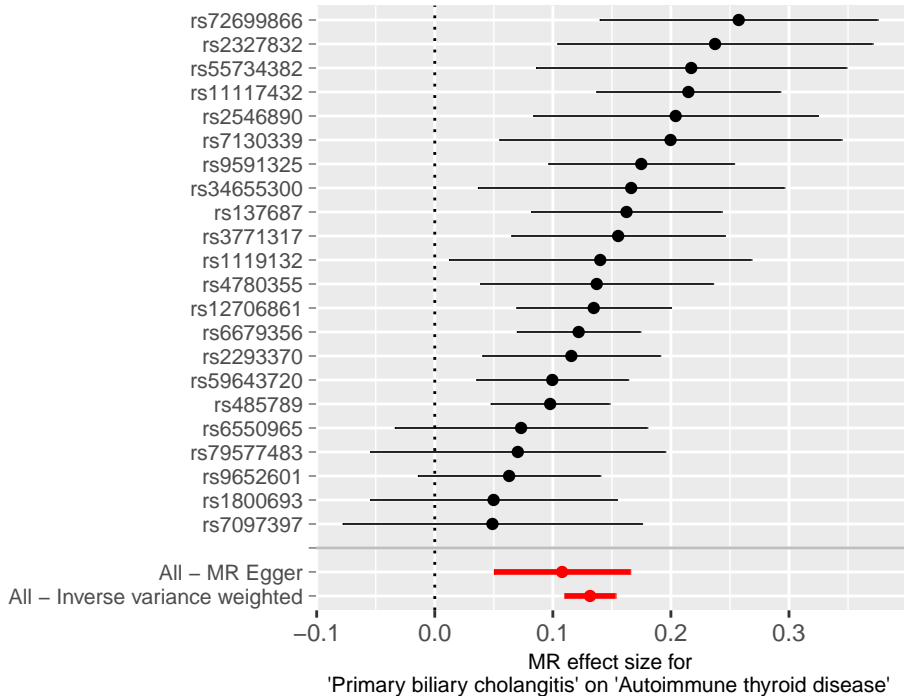

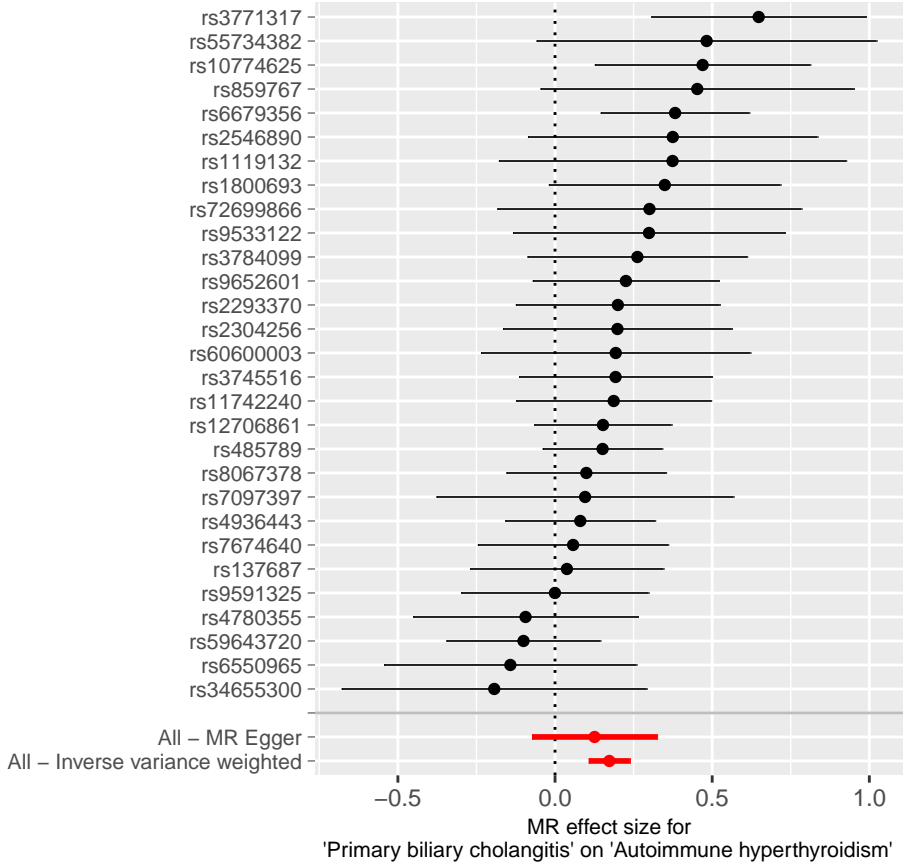

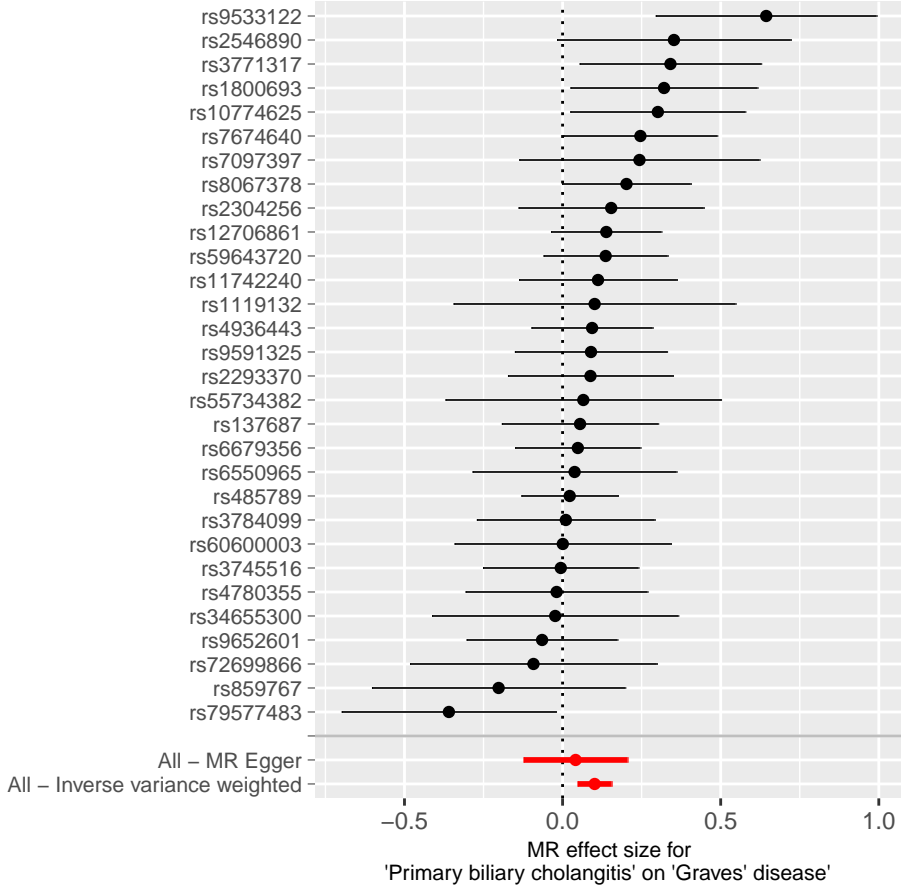

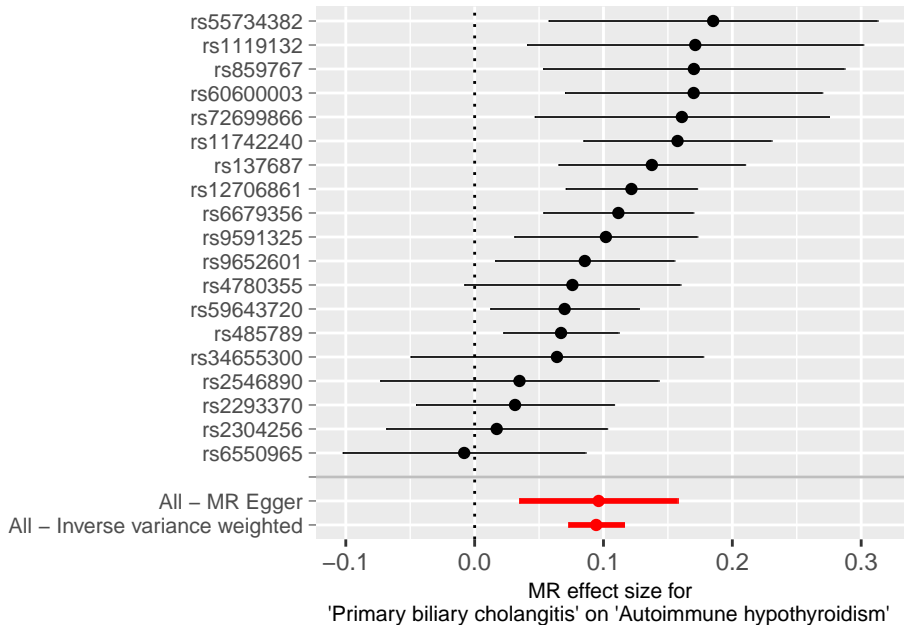

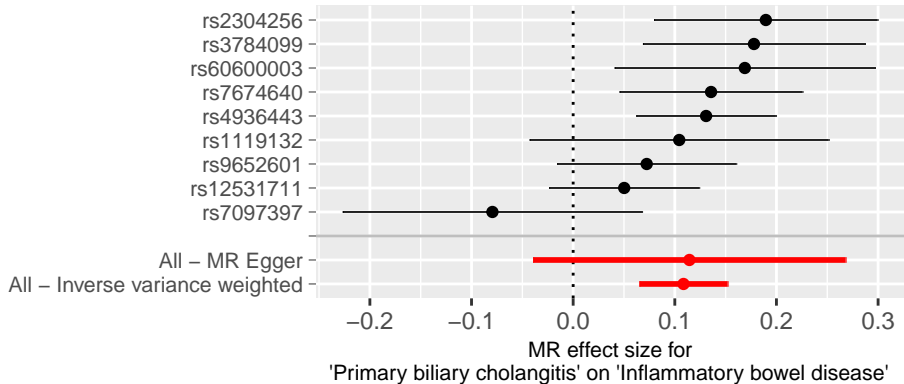

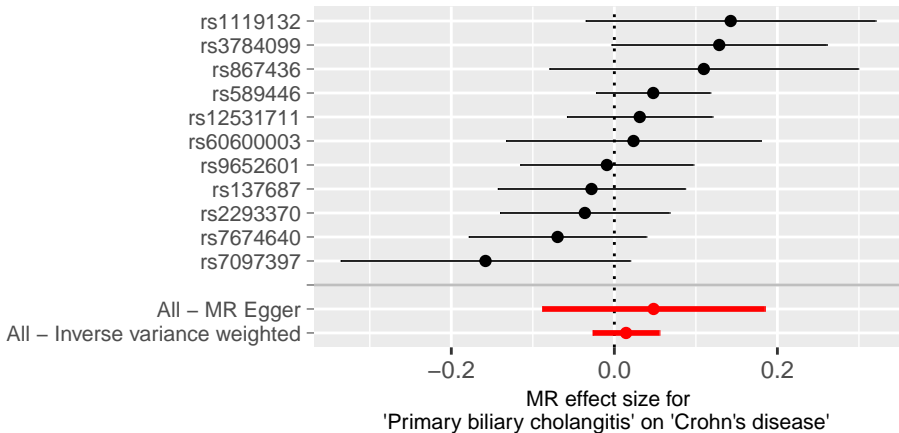

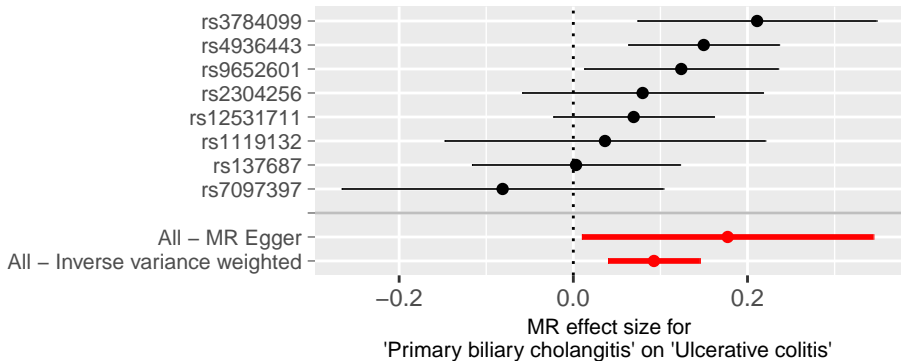

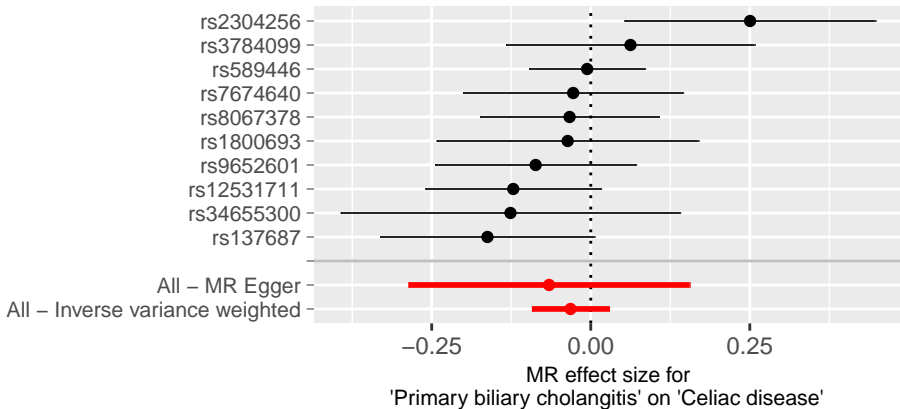

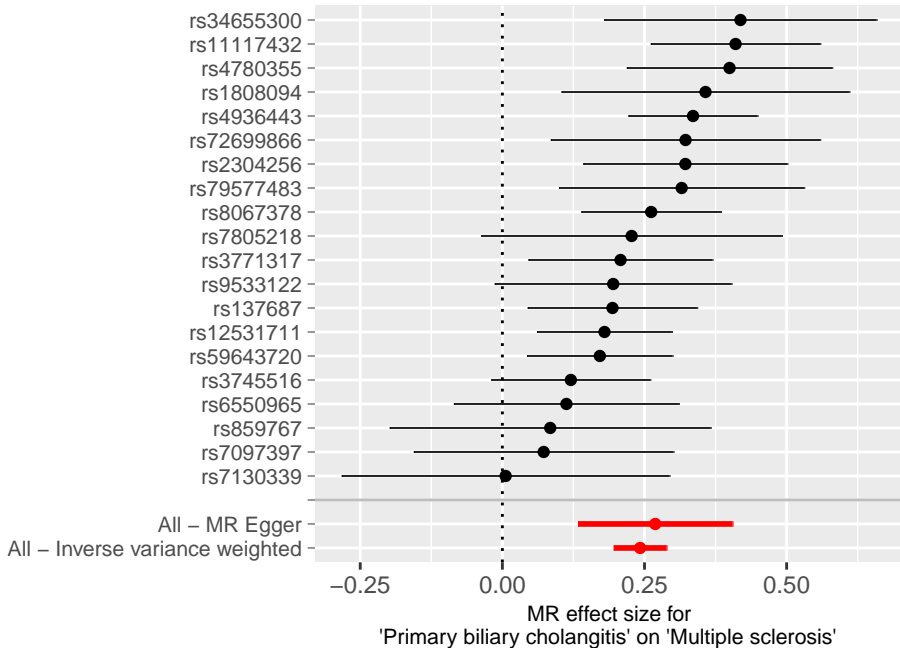

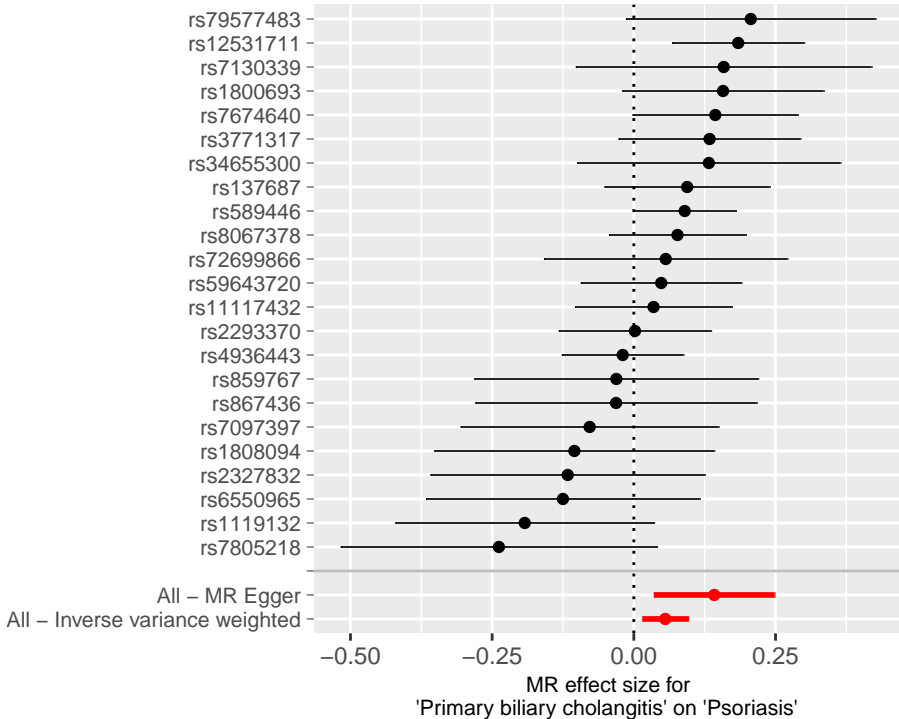

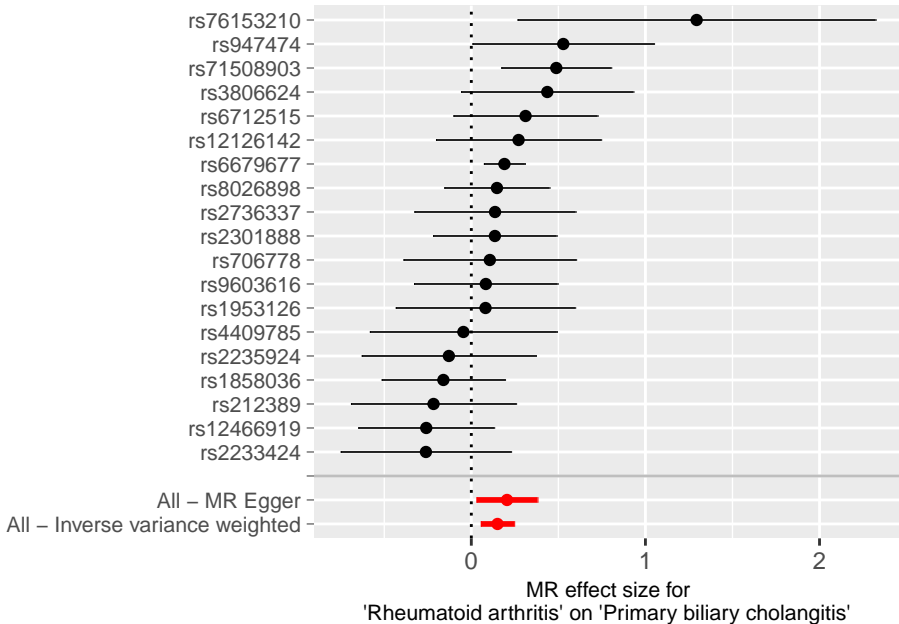

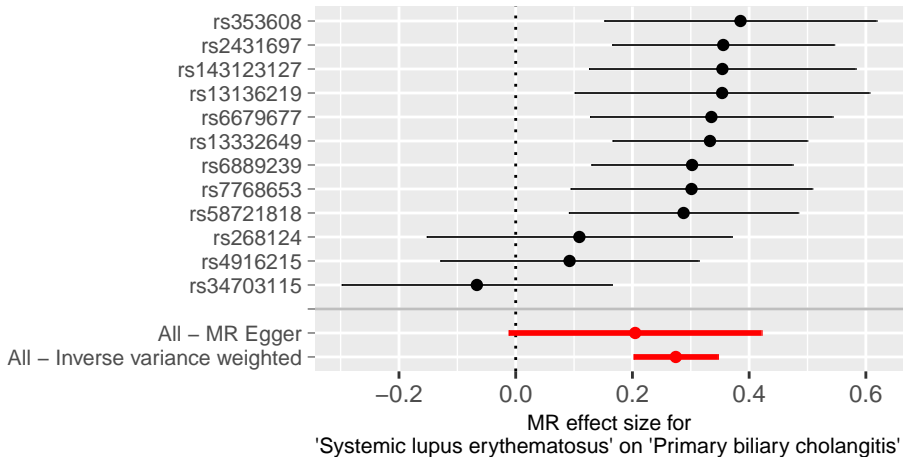

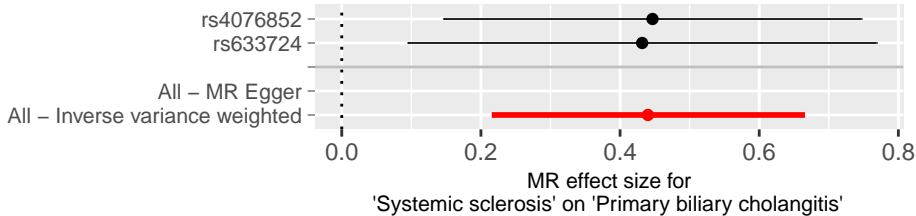

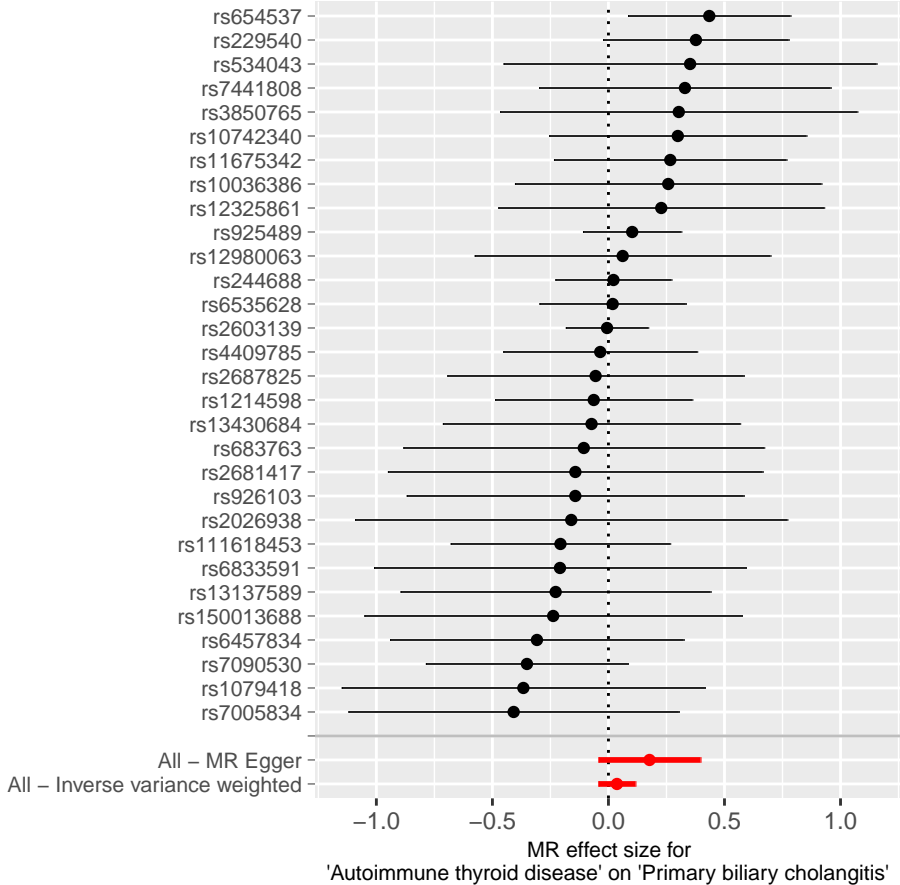

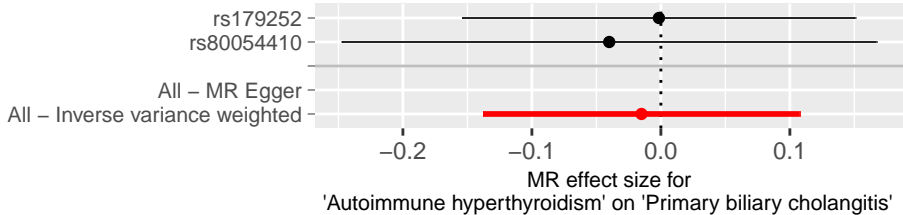

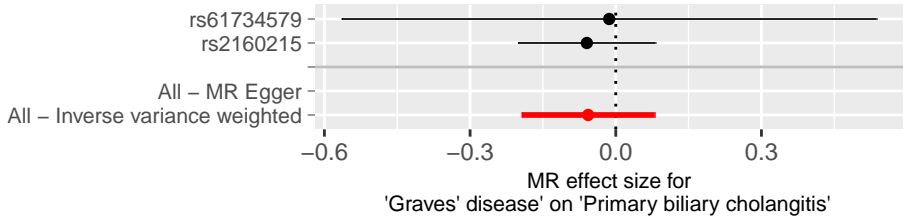

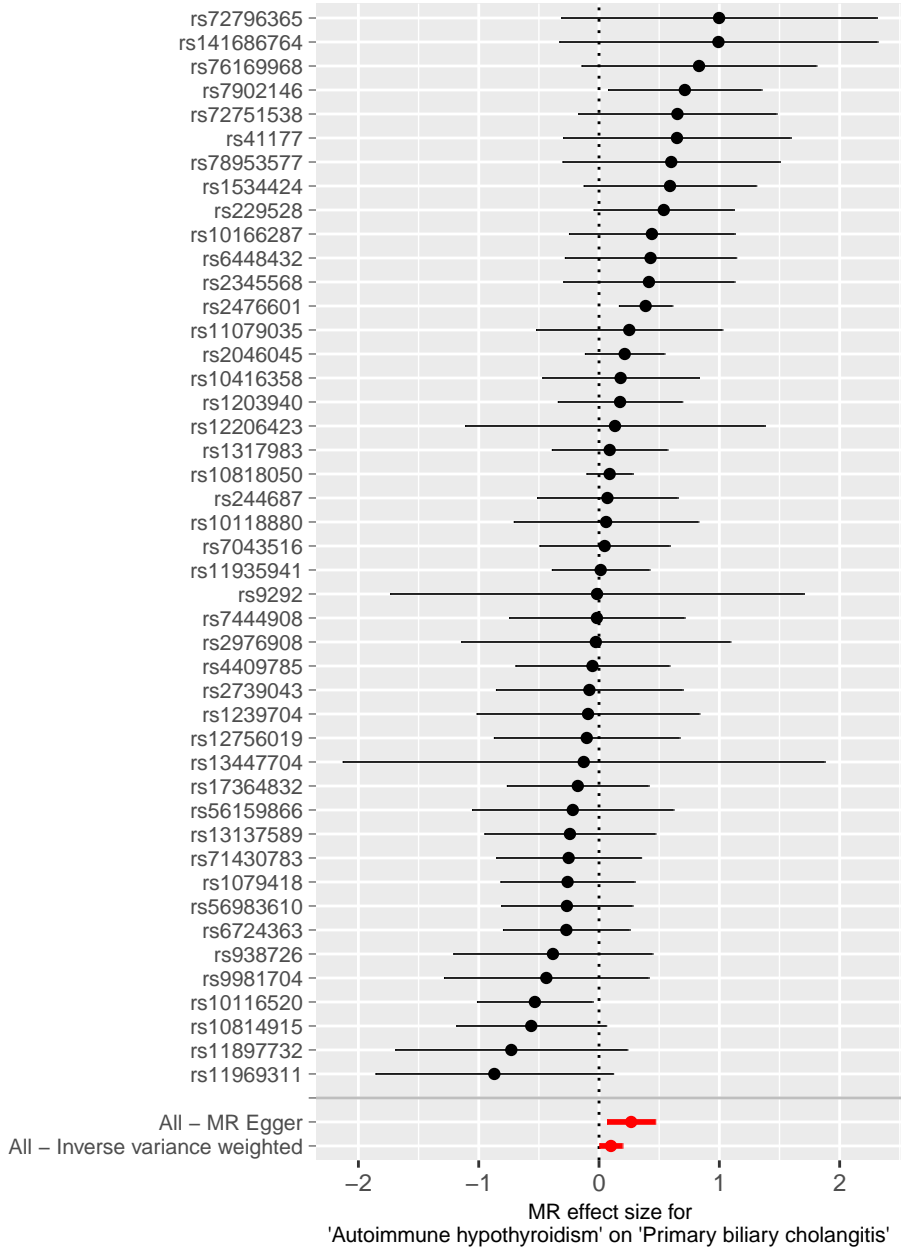

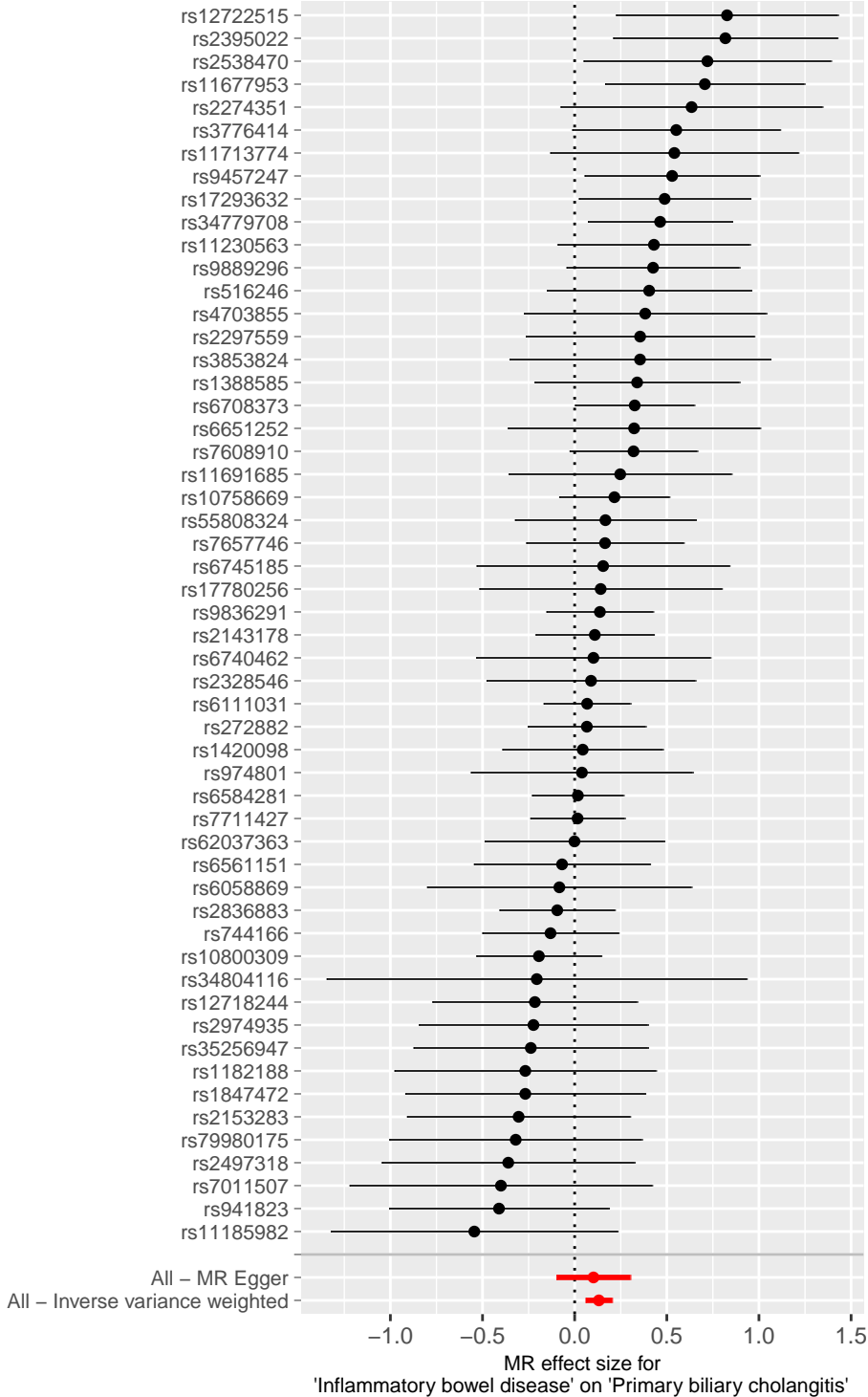

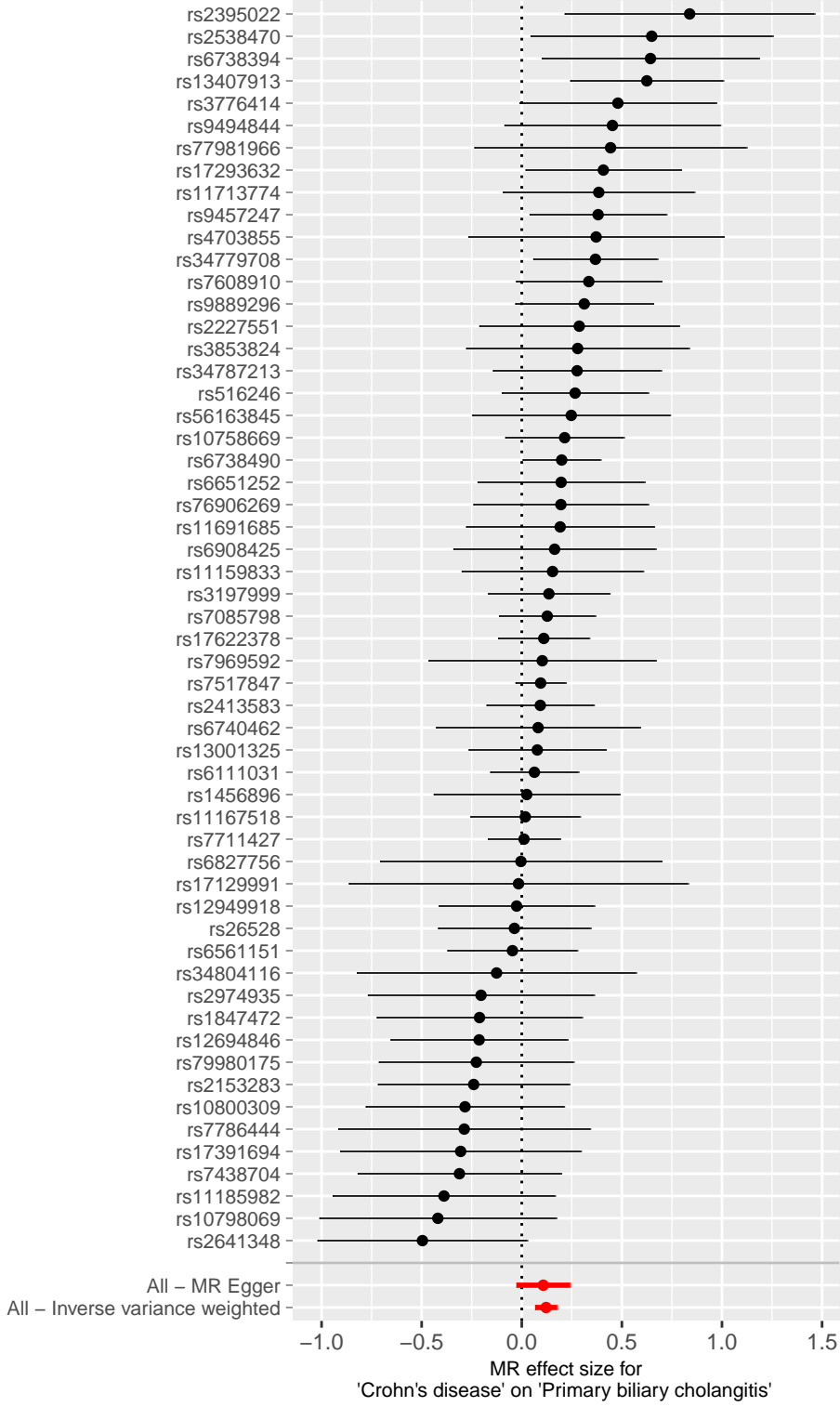

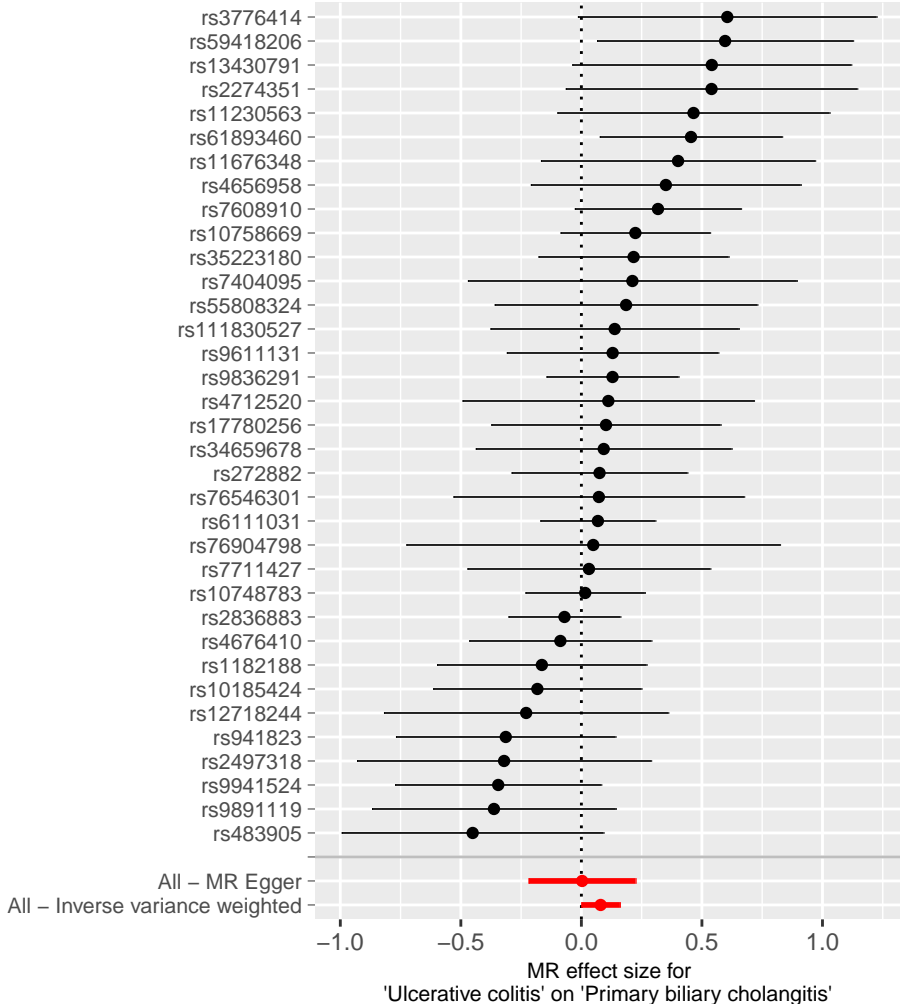

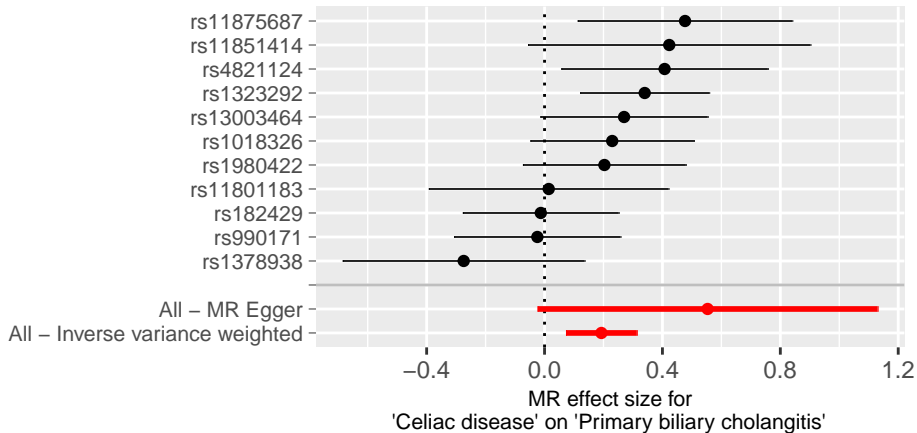

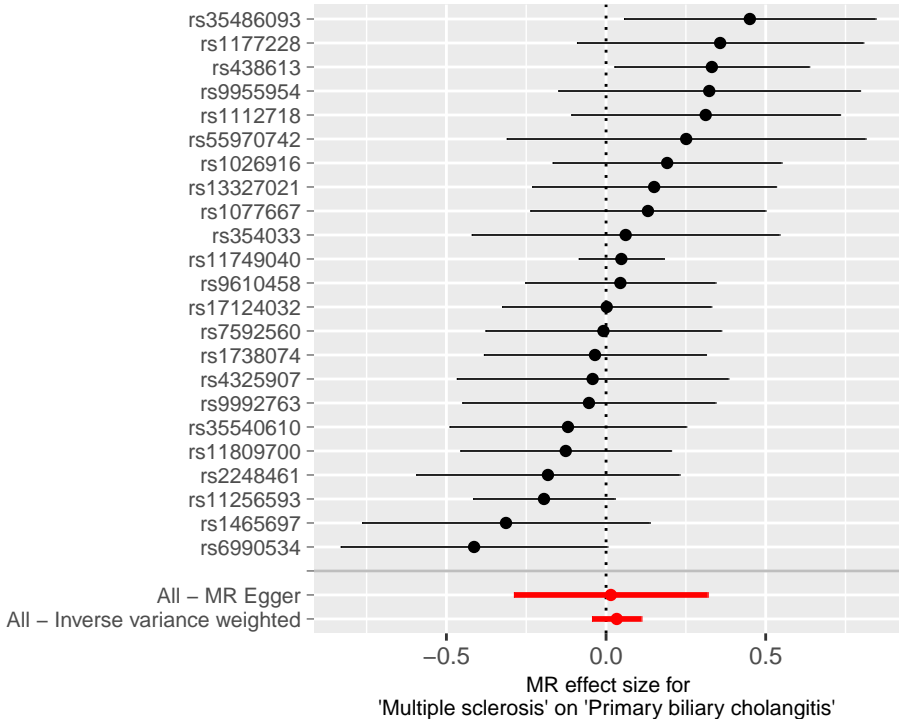

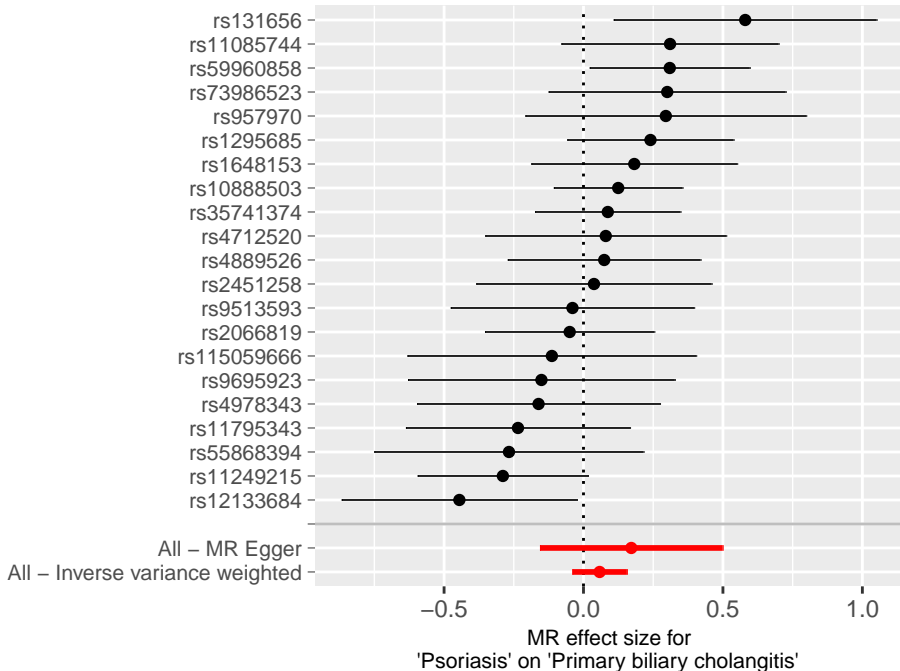

Supplement: Supplementary file 5 — Supplementary Information 4. [file 41598_2024_62509_MOESM5_ESM.pdf]
