# Supplementary material for: Dissecting causal relationships between primary biliary cholangitis and extrahepatic autoimmune diseases based on Mendelian randomization
Source: Sci Rep. 2024 May 21;14:11528. doi: 10.1038/s41598-024-62509-x (PMC11109240; doi:10.1038/s41598-024-62509-x)
Supplement: Supplementary file 6 — Supplementary Information 5. [file 41598_2024_62509_MOESM6_ESM.pdf]

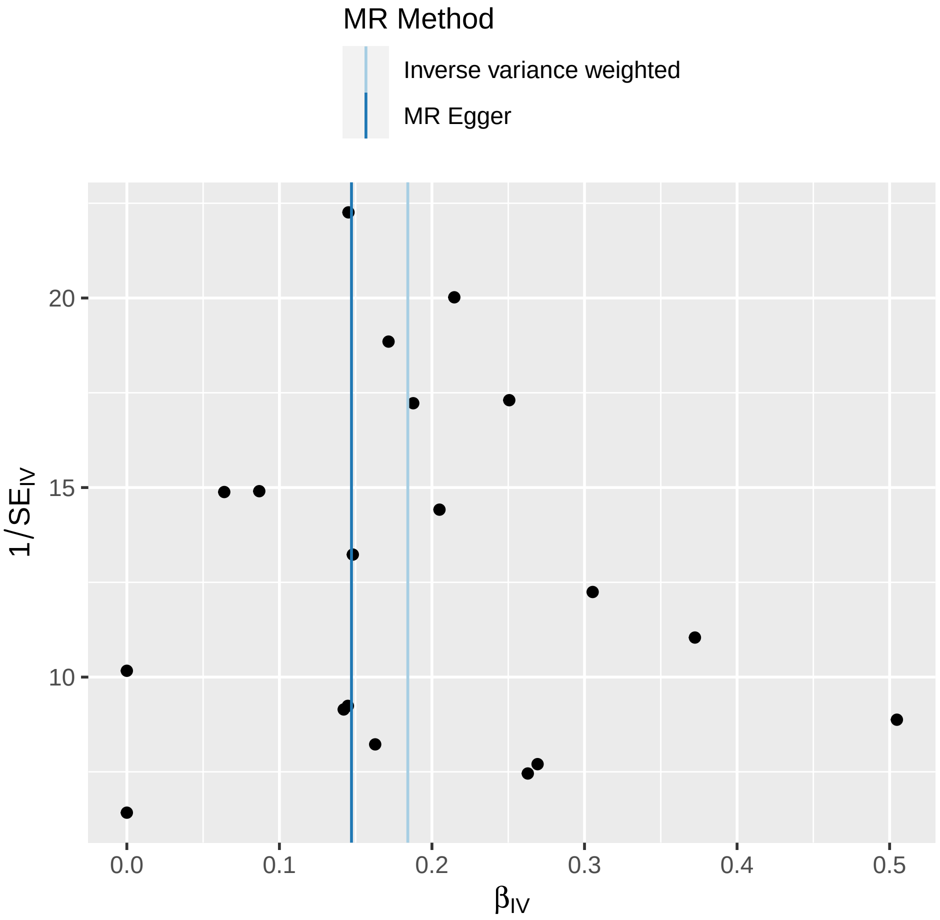

MR Method
Inverse variance weighted
MR Egger
1/SE_IV
20
15
10
0.0
0.1
0.2
0.3
0.4
0.5
β_IV

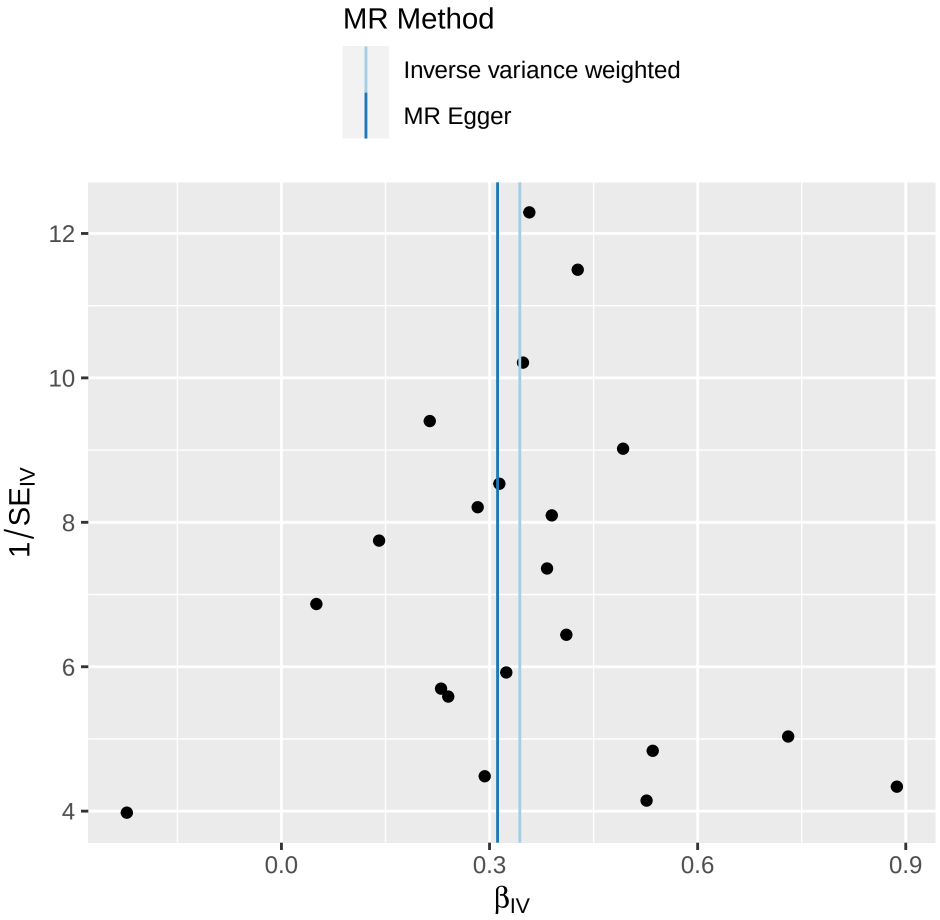

MR Method
Inverse variance weighted
MR Egger
12
10
8
6
4
0.0
0.3
0.6
0.9
$1/SE_{IV}$
$\beta_{IV}$

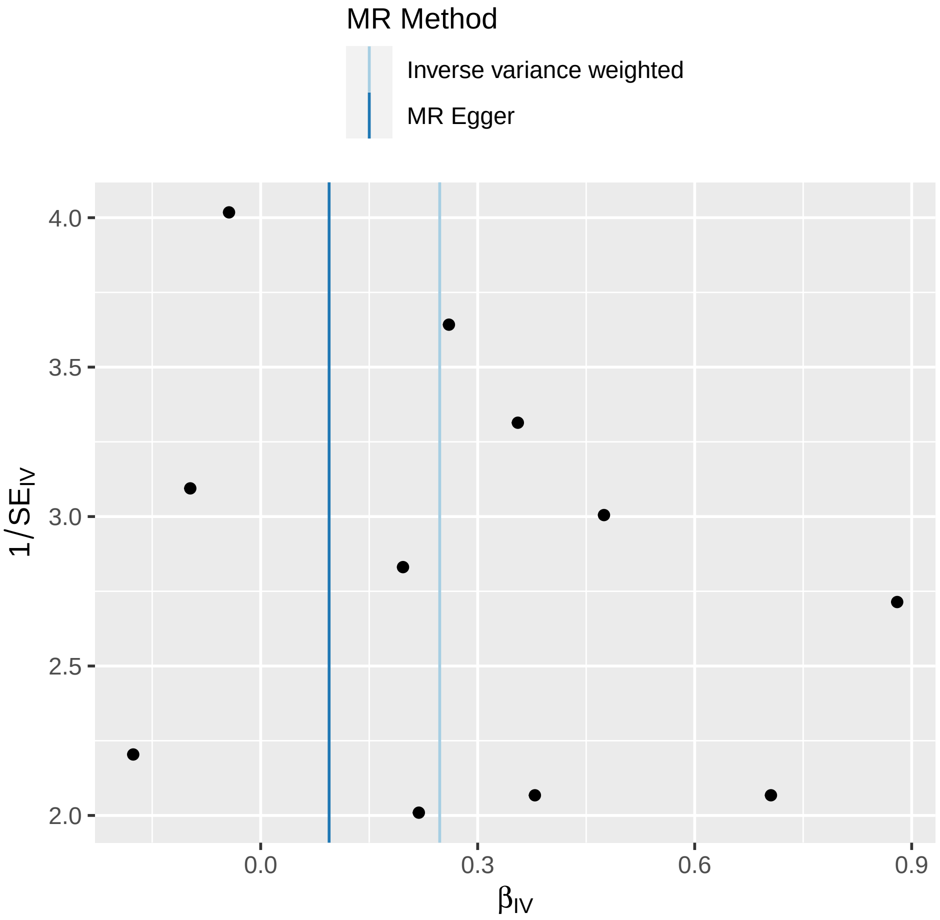
MR Method
Inverse variance weighted
MR Egger
4.0
3.5
3.0
2.5
2.0
$1/SE_{IV}$
0.0
0.3
0.6
0.9
$\beta_{IV}$

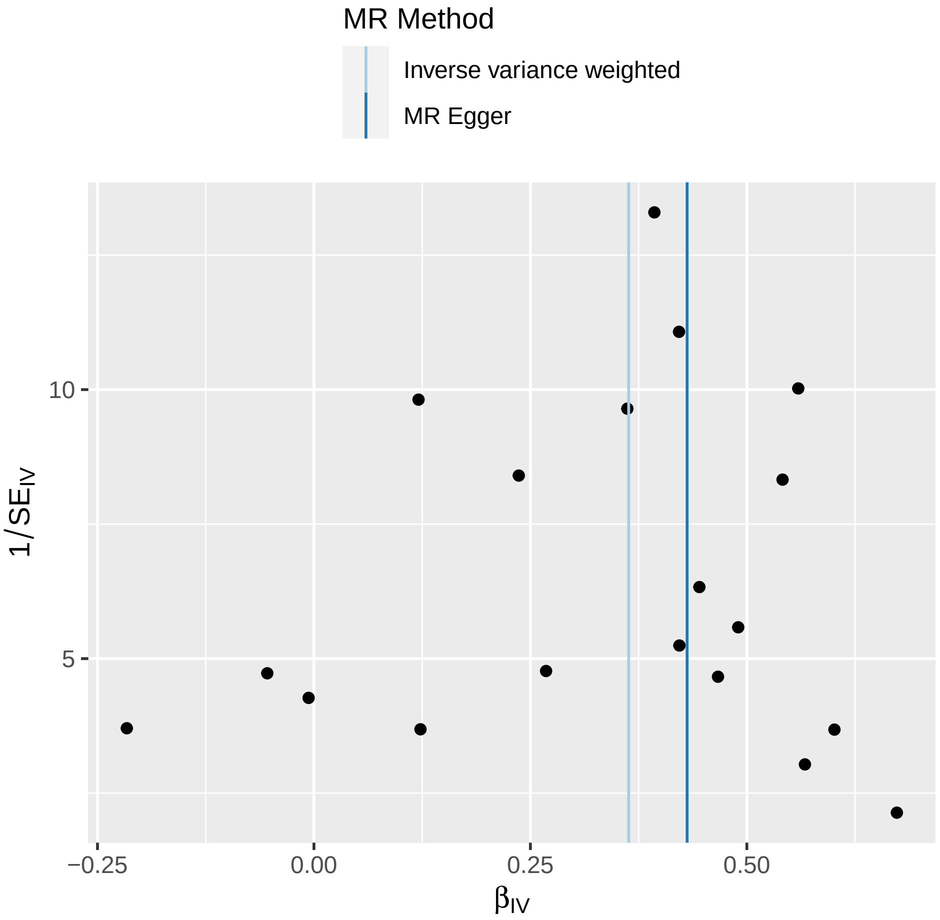
MR Method
Inverse variance weighted
MR Egger
10
5
1/$SE_{IV}$
−0.25
0.00
0.25
0.50
$\beta_{IV}$

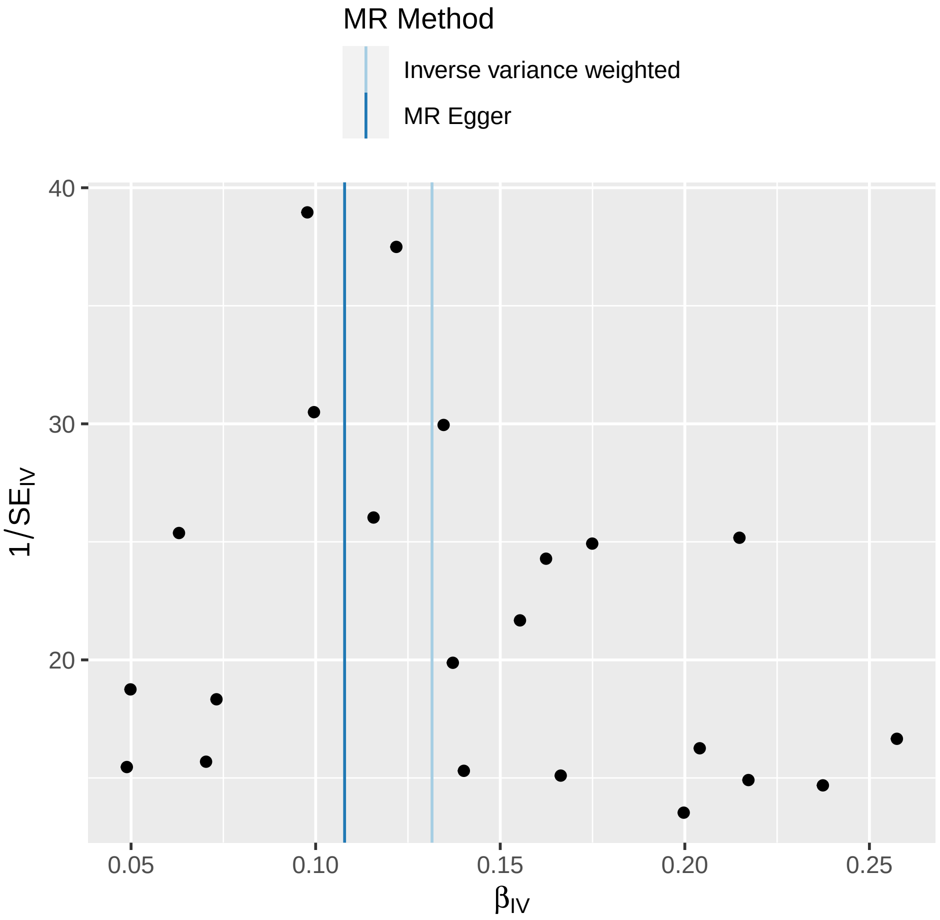
MR Method
Inverse variance weighted
MR Egger
40
30
20
1/SE_IV
0.05
0.10
0.15
0.20
0.25
β_IV

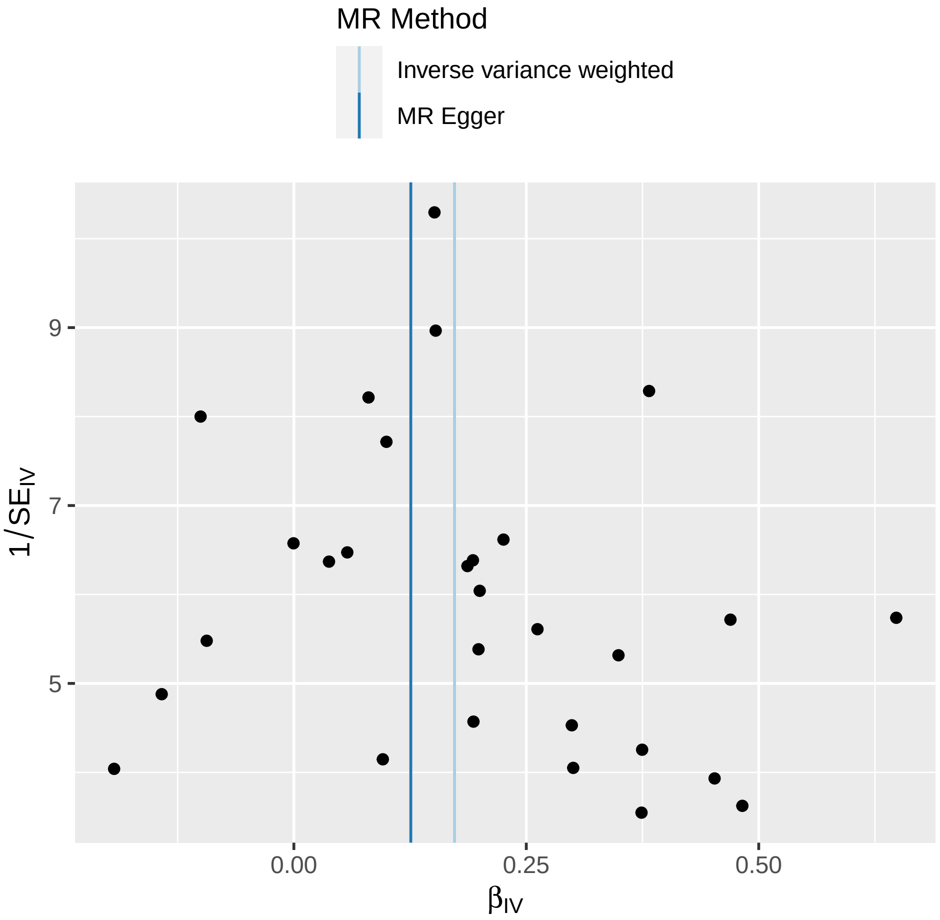

MR Method
Inverse variance weighted
MR Egger
9
7
5
1/SE_IV
0.00
0.25
0.50
β_IV

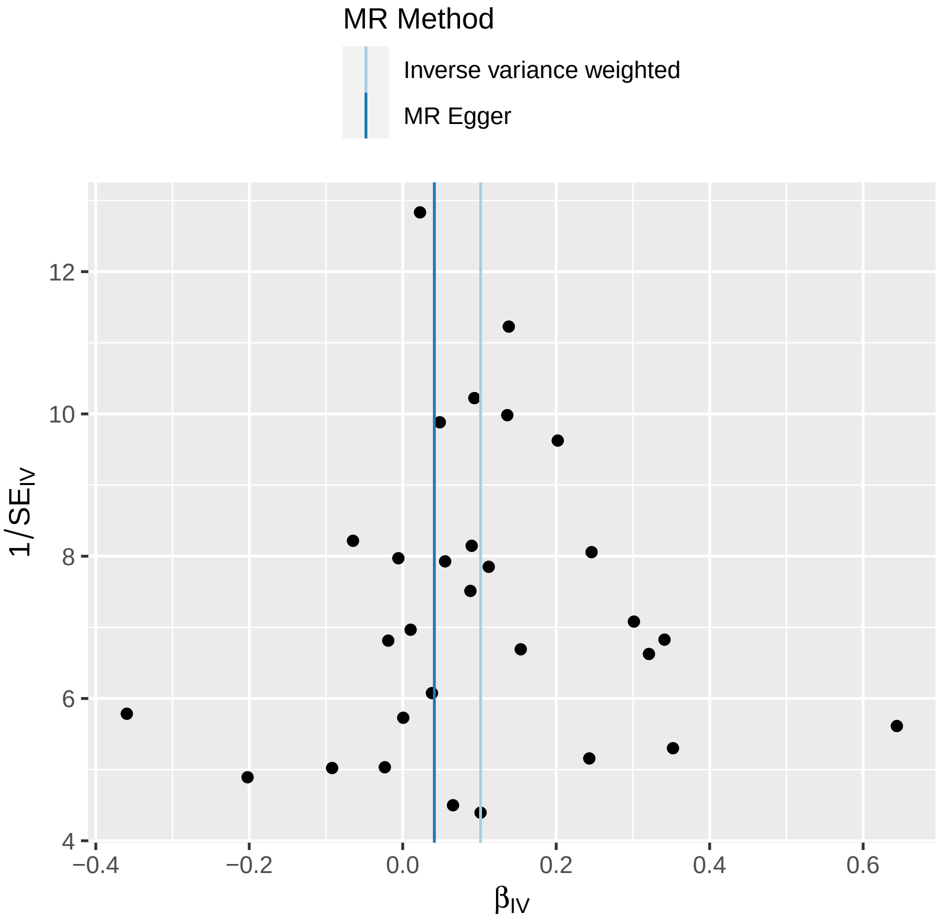

MR Method
Inverse variance weighted
MR Egger
12
10
8
6
4
−0.4
−0.2
0.0
0.2
0.4
0.6
1/SE_IV
β_IV

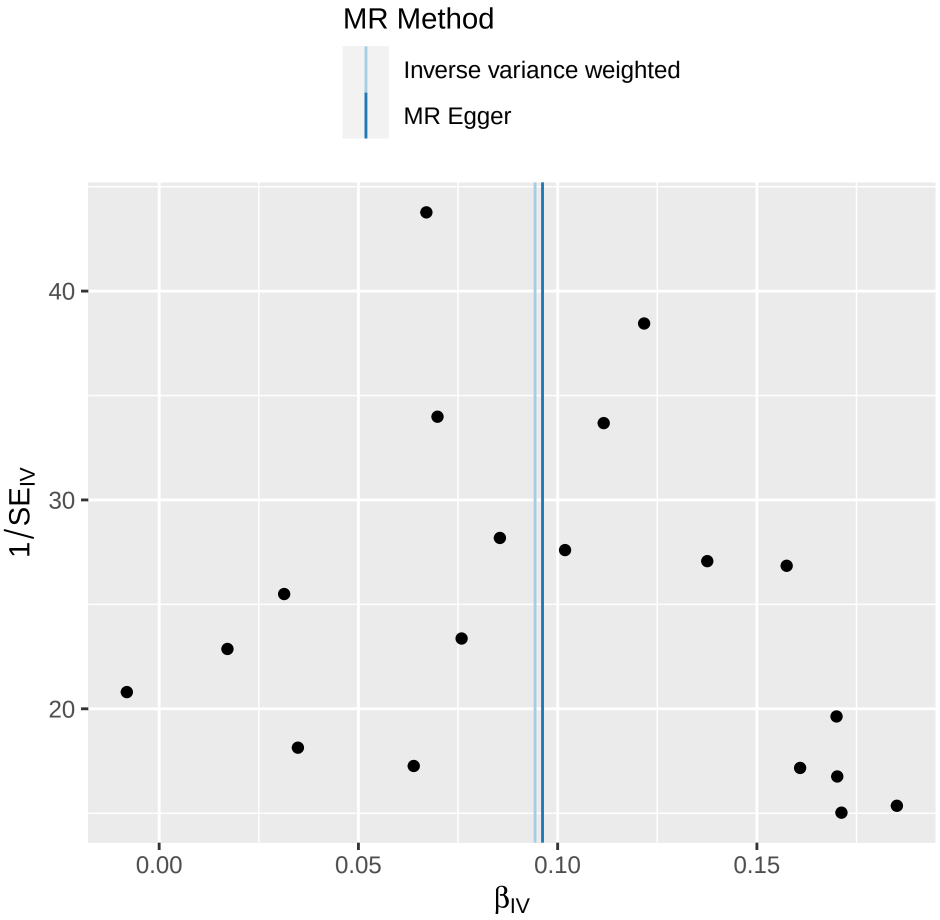
MR Method
Inverse variance weighted
MR Egger
40
30
20
1/SE$_{IV}$
0.00
0.05
0.10
0.15
β$_{IV}$

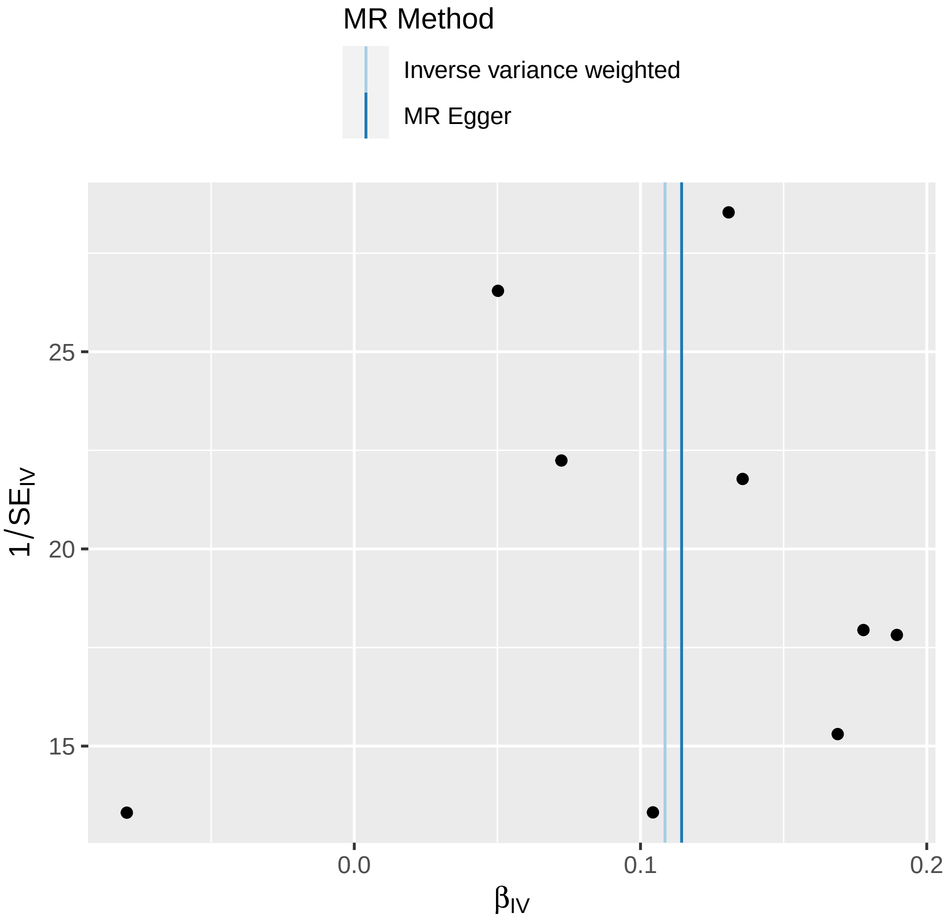
MR Method
Inverse variance weighted
MR Egger
25
20
15
1/SE_IV
0.0
0.1
0.2
β_IV

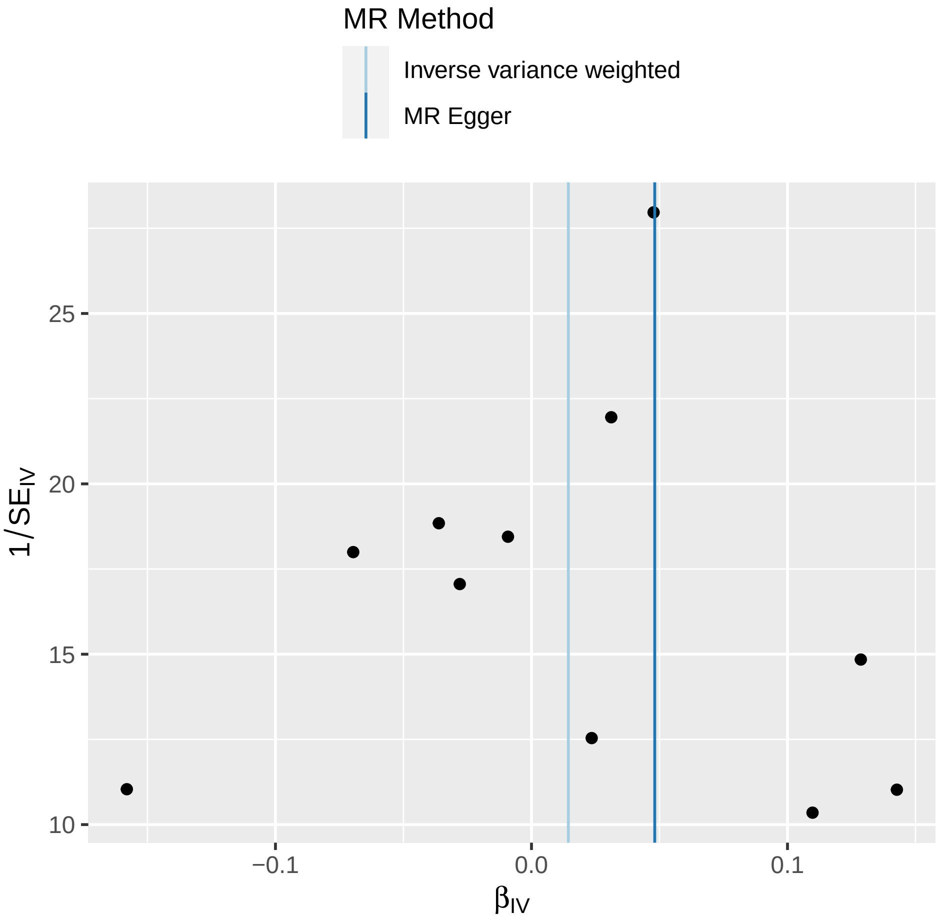
MR Method
Inverse variance weighted
MR Egger
25
20
15
10
1/SE_IV
−0.1
0.0
0.1
β_IV

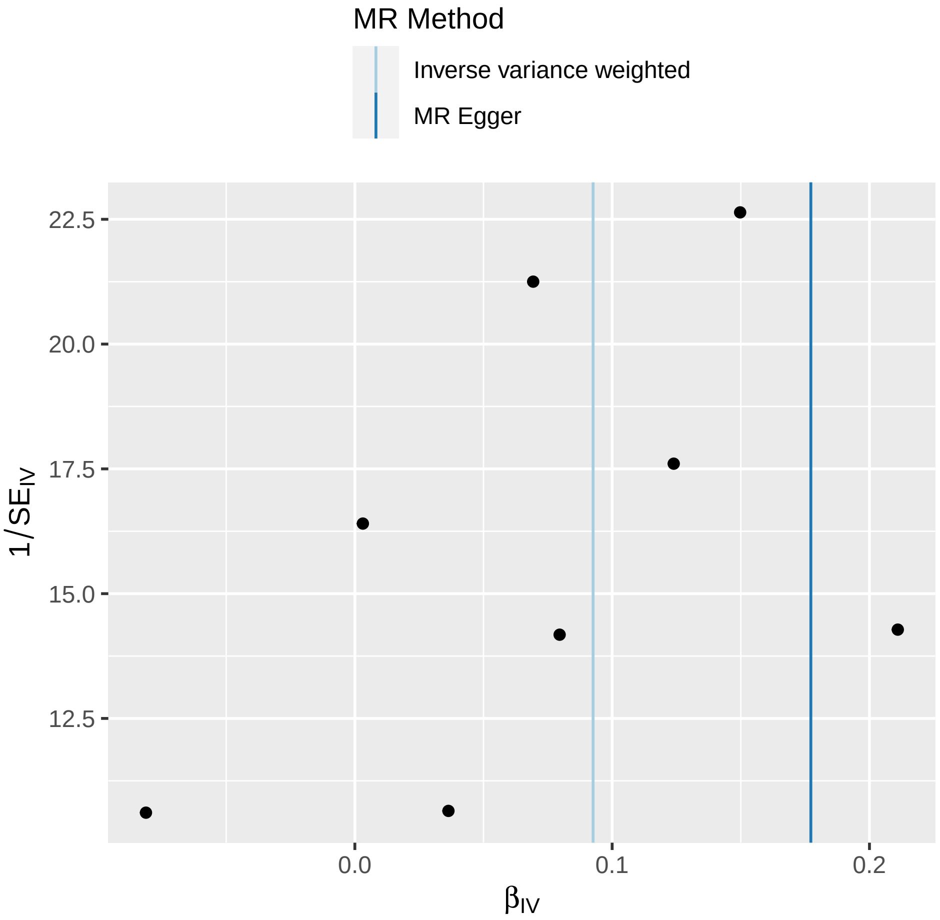
MR Method
Inverse variance weighted
MR Egger
22.5
20.0
17.5
15.0
12.5
1/SE_IV
0.0
0.1
0.2
β_IV

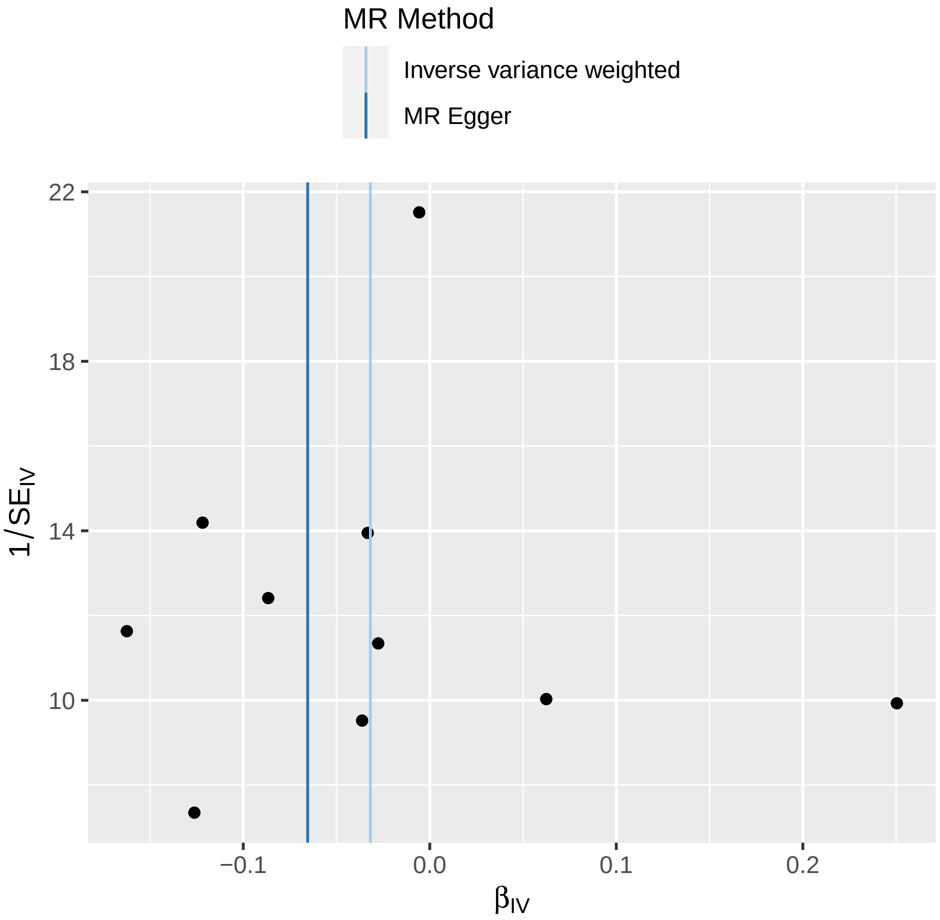
MR Method
Inverse variance weighted
MR Egger
22
18
14
10
1/SE_IV
−0.1
0.0
0.1
0.2
β_IV

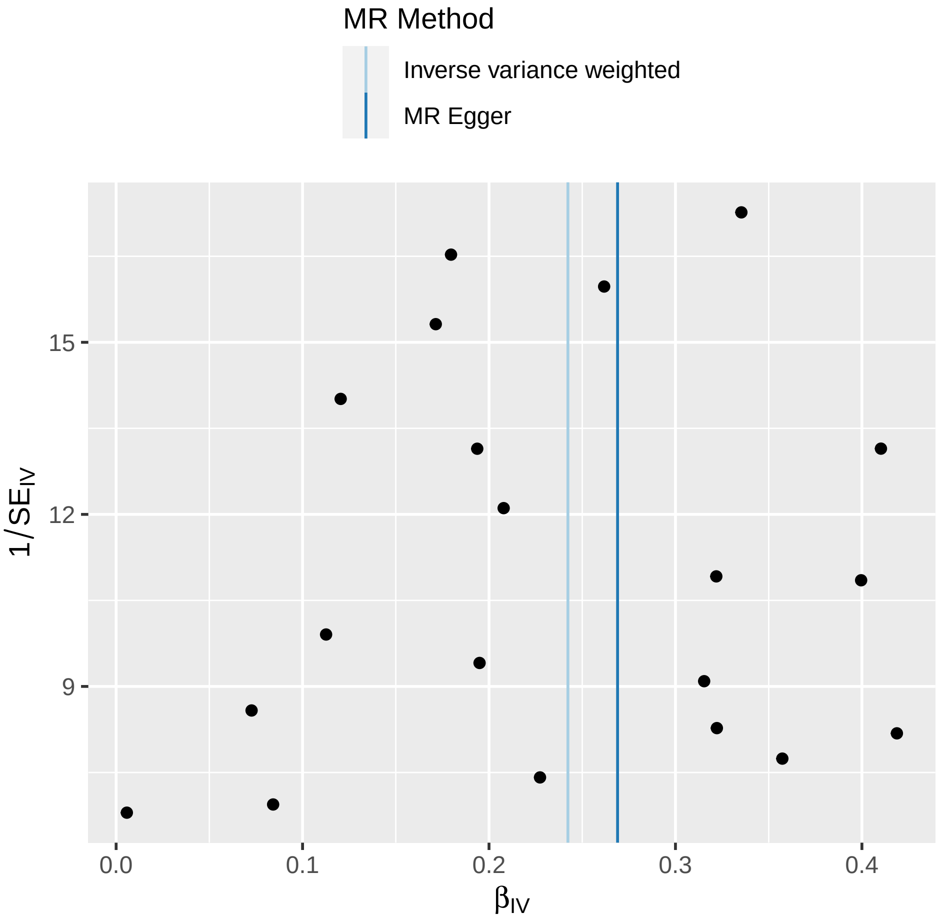

MR Method
Inverse variance weighted
MR Egger
15
12
9
1/SE_IV
0.0
0.1
0.2
0.3
0.4
β_IV

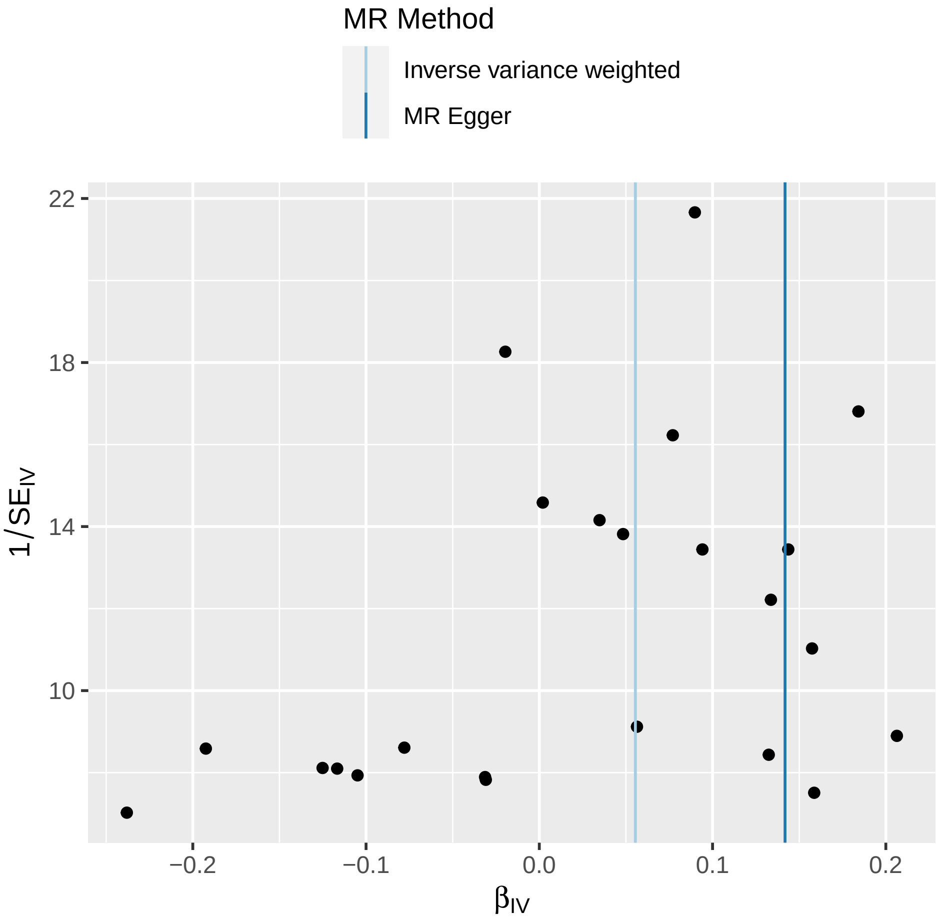
MR Method
Inverse variance weighted
MR Egger
22
18
14
10
−0.2
−0.1
0.0
0.1
0.2
1/SE_IV
β_IV

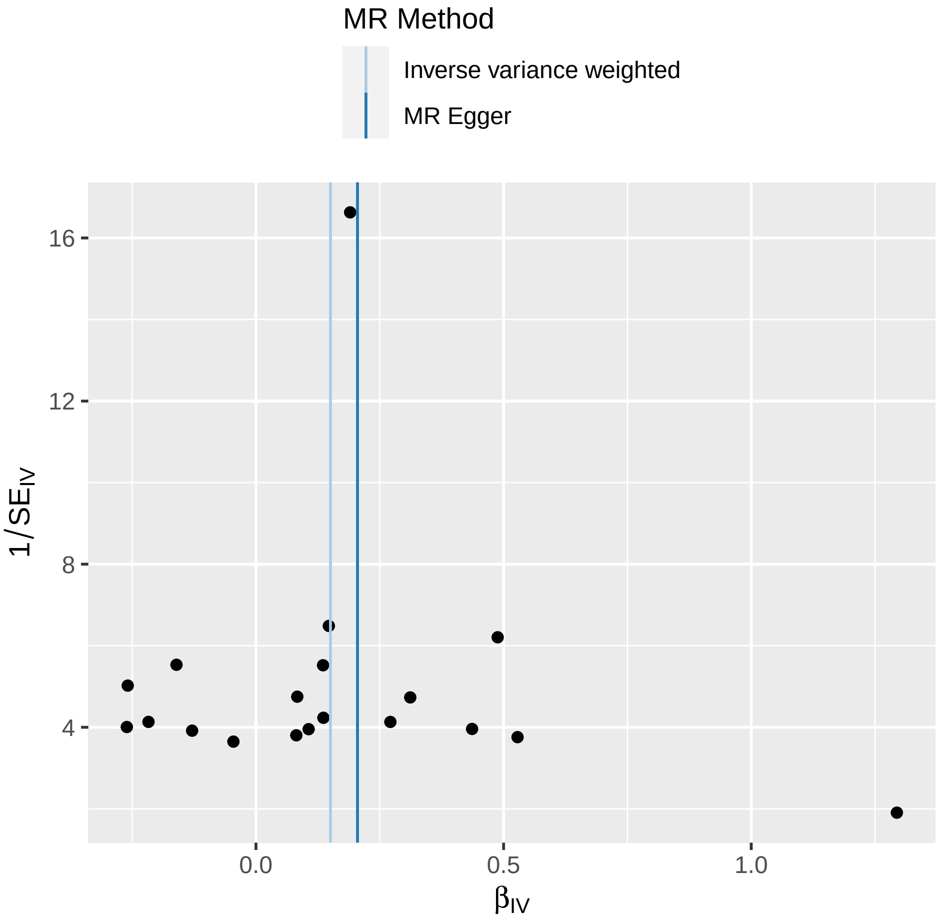
MR Method
Inverse variance weighted
MR Egger
16
12
8
4
0.0
0.5
1.0
$\beta_{IV}$
$1/SE_{IV}$

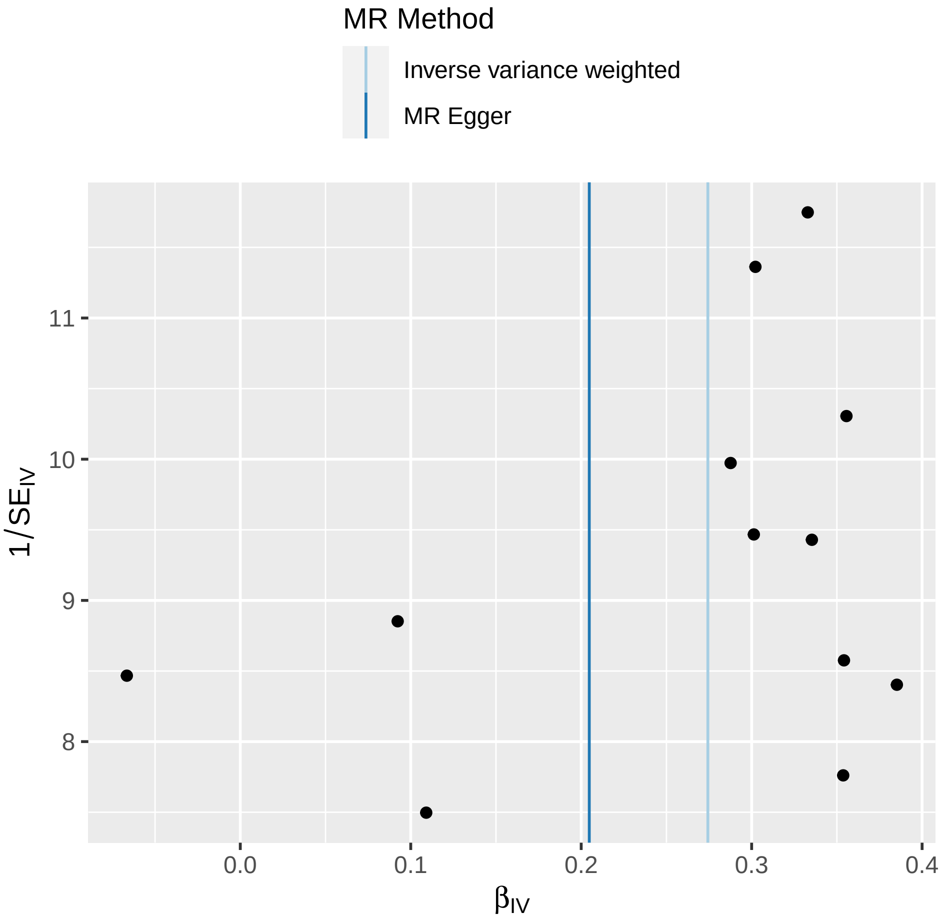
MR Method
Inverse variance weighted
MR Egger
11
10
9
8
0.0
0.1
0.2
0.3
0.4
$1/SE_{IV}$
$\beta_{IV}$

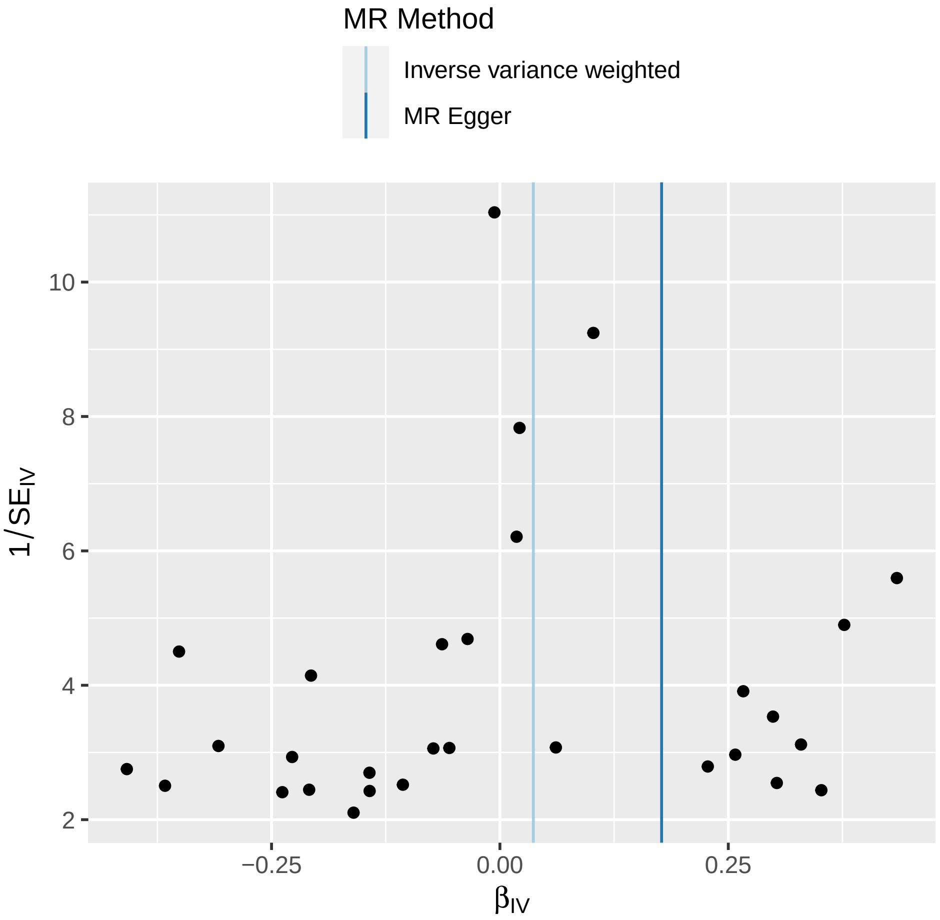
MR Method
Inverse variance weighted
MR Egger
10
8
6
4
2
1/SE_IV
−0.25
0.00
0.25
β_IV

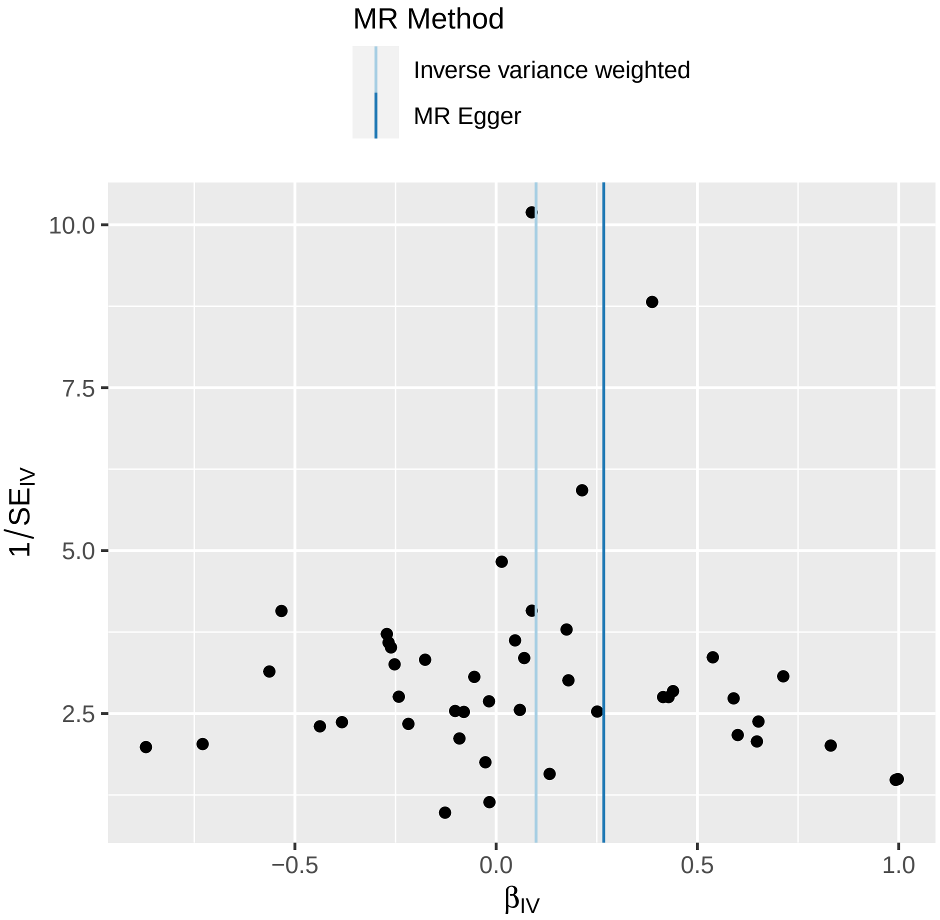

MR Method
Inverse variance weighted
MR Egger
10.0
7.5
5.0
2.5
1/SE_IV
−0.5
0.0
0.5
1.0
β_IV

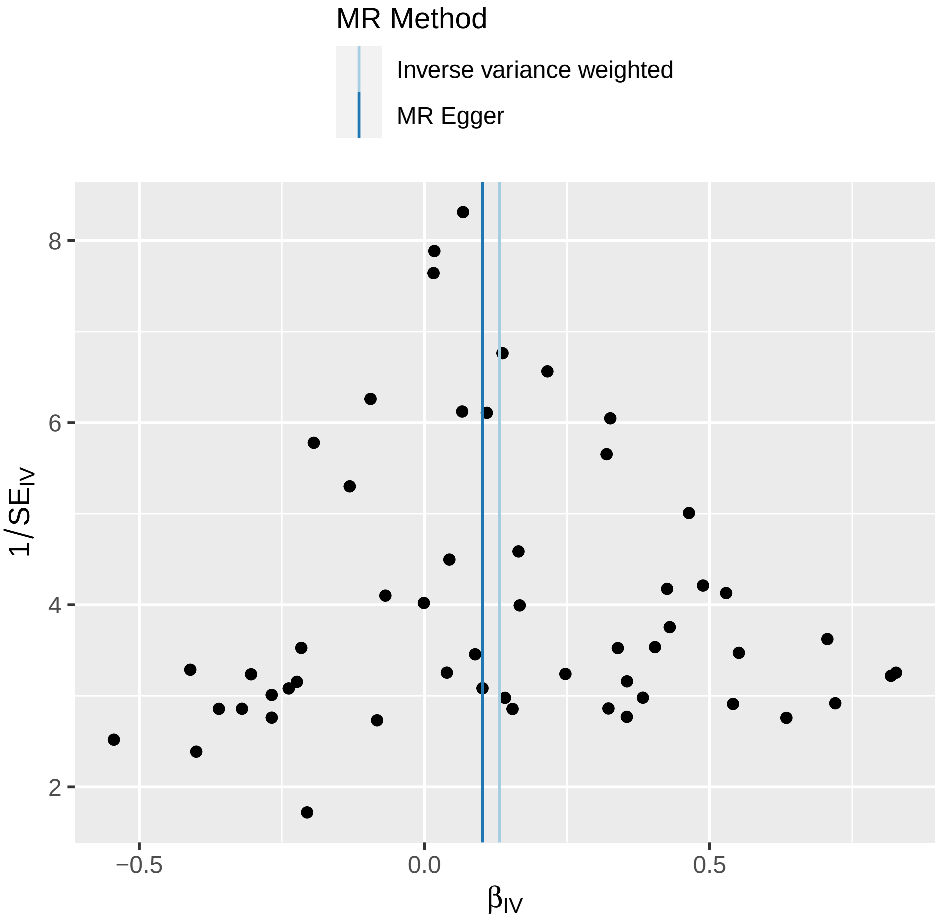
MR Method
Inverse variance weighted
MR Egger
8
6
4
2
1/SE_IV
−0.5
0.0
0.5
β_IV

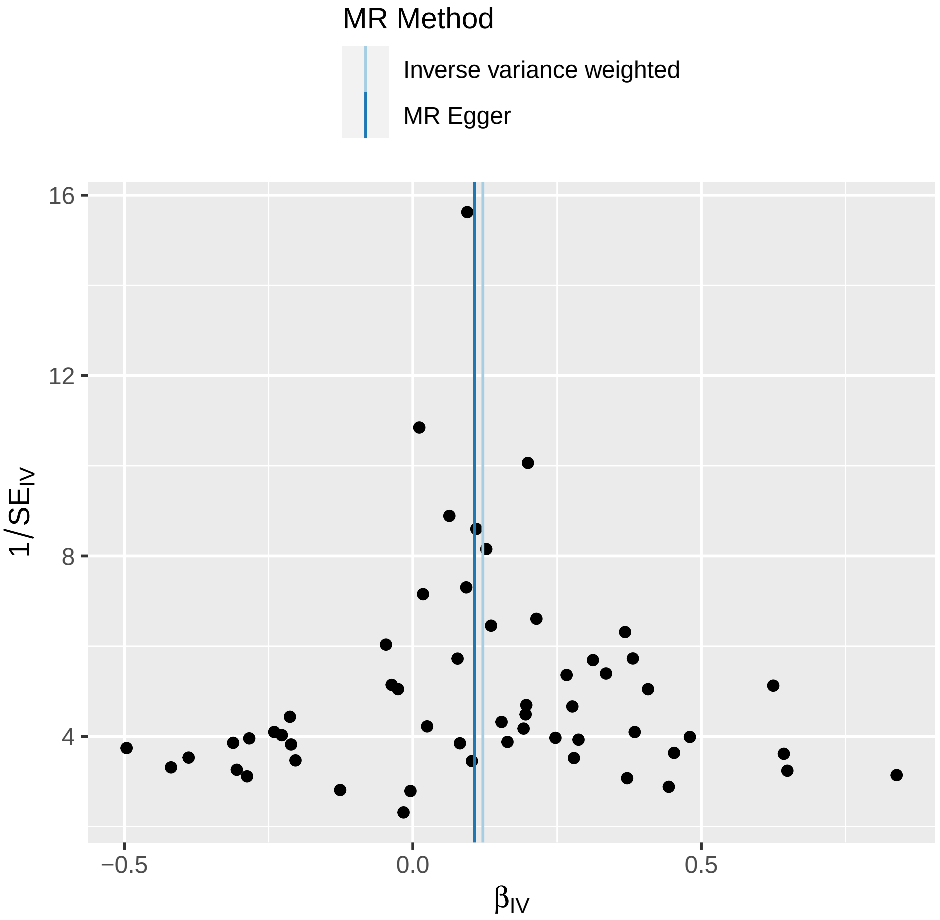

MR Method
Inverse variance weighted
MR Egger
16
12
8
4
1/SE$_{IV}$
−0.5
0.0
0.5
β$_{IV}$

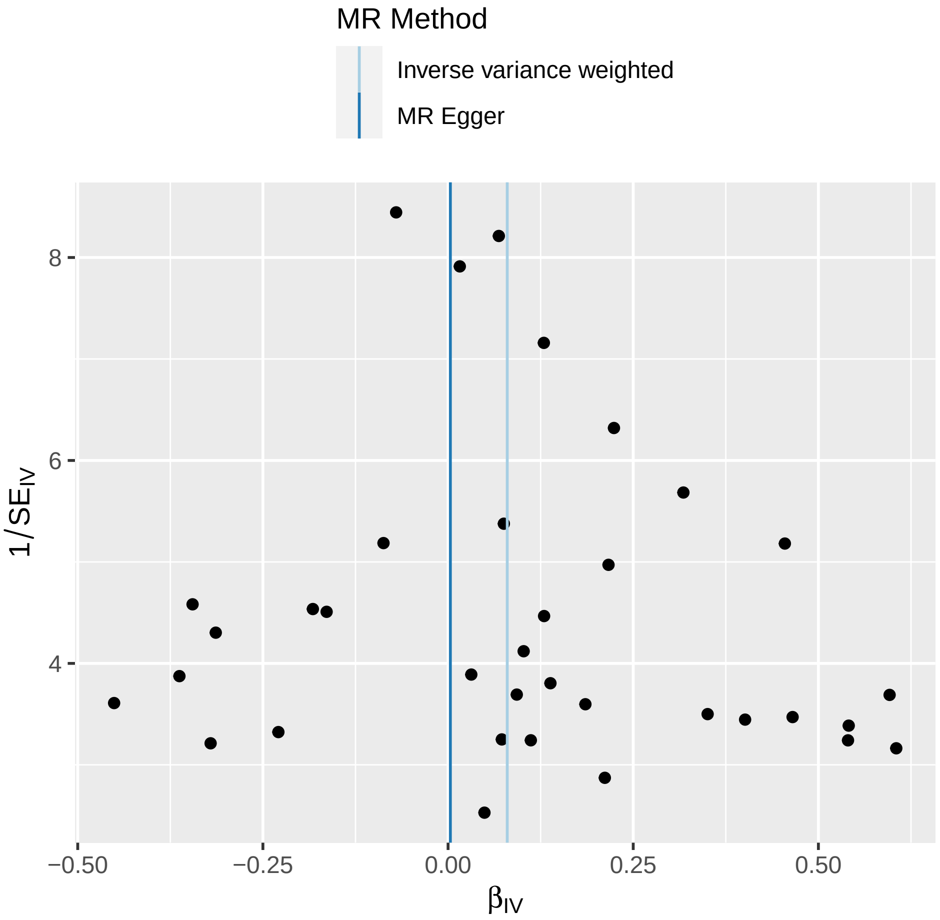
MR Method
Inverse variance weighted
MR Egger
8
6
4
1/SE_IV
−0.50
−0.25
0.00
0.25
0.50
β_IV

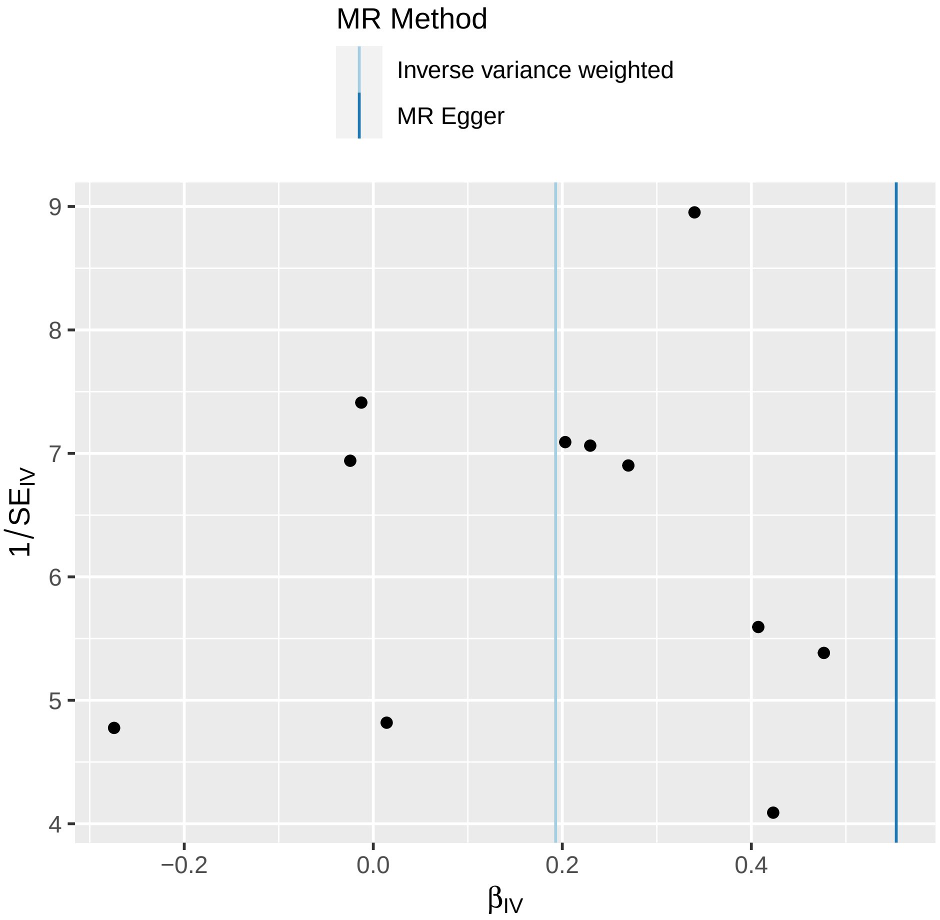
MR Method
Inverse variance weighted
MR Egger
9
8
7
6
5
4
−0.2
0.0
0.2
0.4
1/SE_IV
β_IV

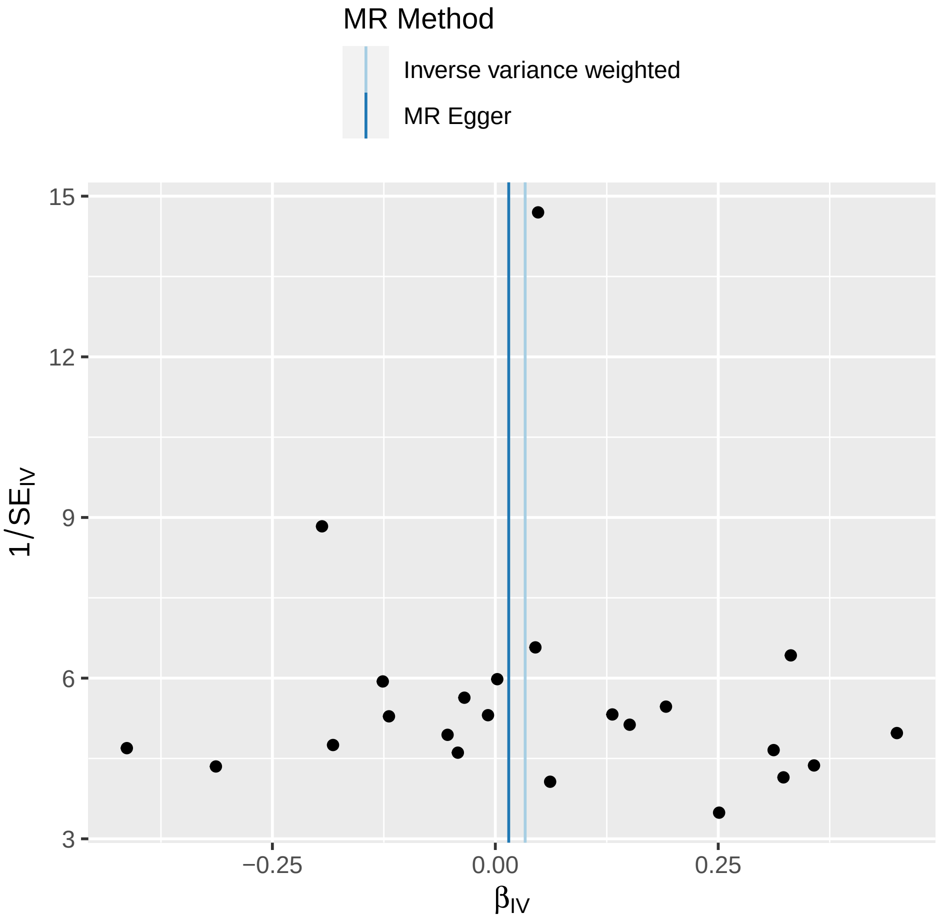

MR Method
Inverse variance weighted
MR Egger
15
12
9
6
3
1/SE_IV
−0.25
0.00
0.25
β_IV

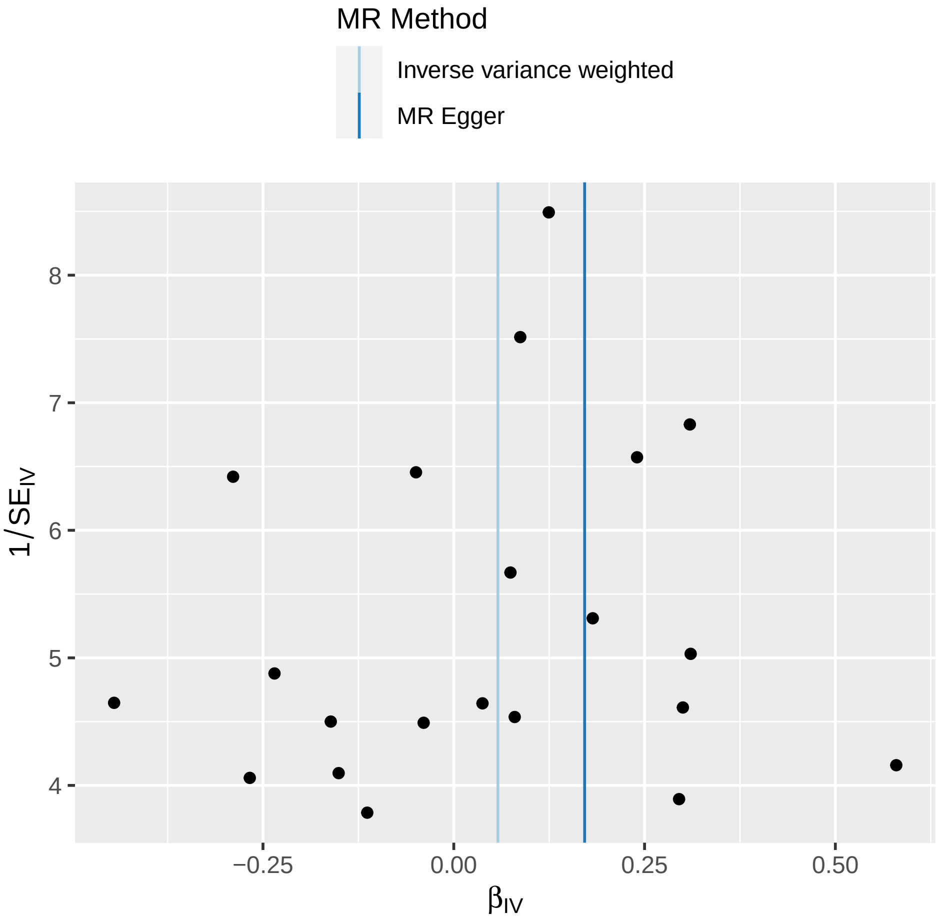

MR Method
Inverse variance weighted
MR Egger
8
7
6
5
4
1/SE_IV
−0.25
0.00
0.25
0.50
β_IV
