# Supplementary material for: Dissecting causal relationships between primary biliary cholangitis and extrahepatic autoimmune diseases based on Mendelian randomization
Source: Sci Rep. 2024 May 21;14:11528. doi: 10.1038/s41598-024-62509-x (PMC11109240; doi:10.1038/s41598-024-62509-x)
Supplement: Supplementary file 7 — Supplementary Information 6. [file 41598_2024_62509_MOESM7_ESM.pdf]

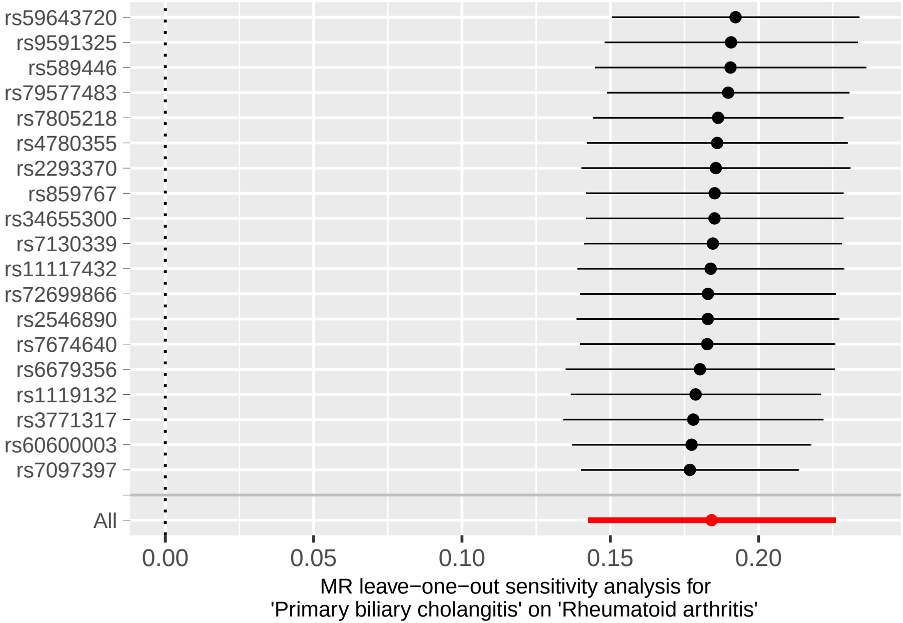
rs59643720
rs9591325
rs589446
rs79577483
rs7805218
rs4780355
rs2293370
rs859767
rs34655300
rs7130339
rs11117432
rs72699866
rs2546890
rs7674640
rs6679356
rs1119132
rs3771317
rs60600003
rs7097397
All
0.00
0.05
0.10
0.15
0.20
MR leave−one−out sensitivity analysis for
'Primary biliary cholangitis' on 'Rheumatoid arthritis'

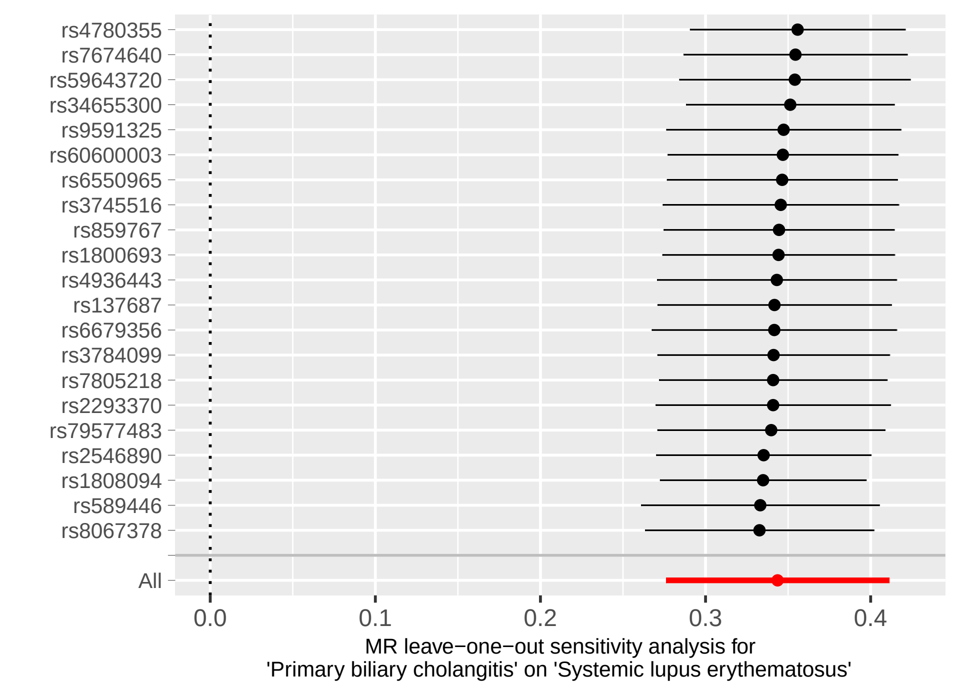
rs4780355
rs7674640
rs59643720
rs34655300
rs9591325
rs60600003
rs6550965
rs3745516
rs859767
rs1800693
rs4936443
rs137687
rs6679356
rs3784099
rs7805218
rs2293370
rs79577483
rs2546890
rs1808094
rs589446
rs8067378
All
0.0
0.1
0.2
0.3
0.4
MR leave−one−out sensitivity analysis for
'Primary biliary cholangitis' on 'Systemic lupus erythematosus'

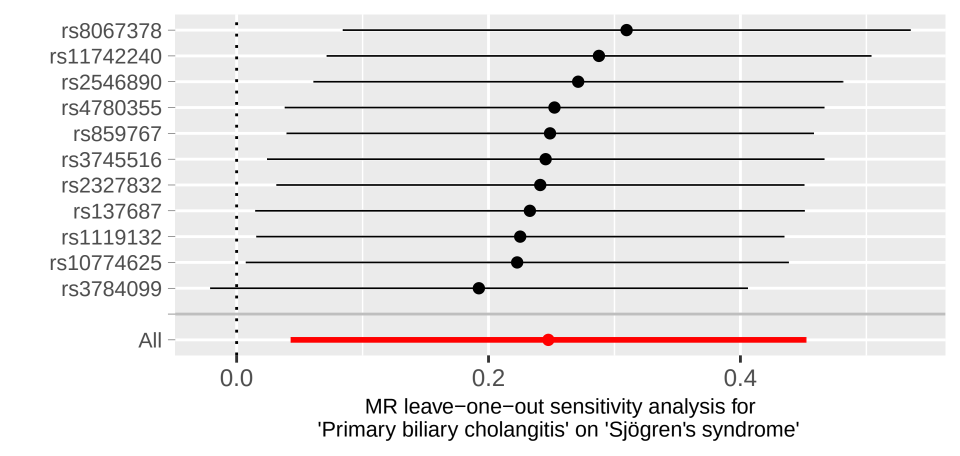
rs8067378
rs11742240
rs2546890
rs4780355
rs859767
rs3745516
rs2327832
rs137687
rs1119132
rs10774625
rs3784099
All
0.0
0.2
0.4
MR leave−one−out sensitivity analysis for
'Primary biliary cholangitis' on 'Sjögren's syndrome'

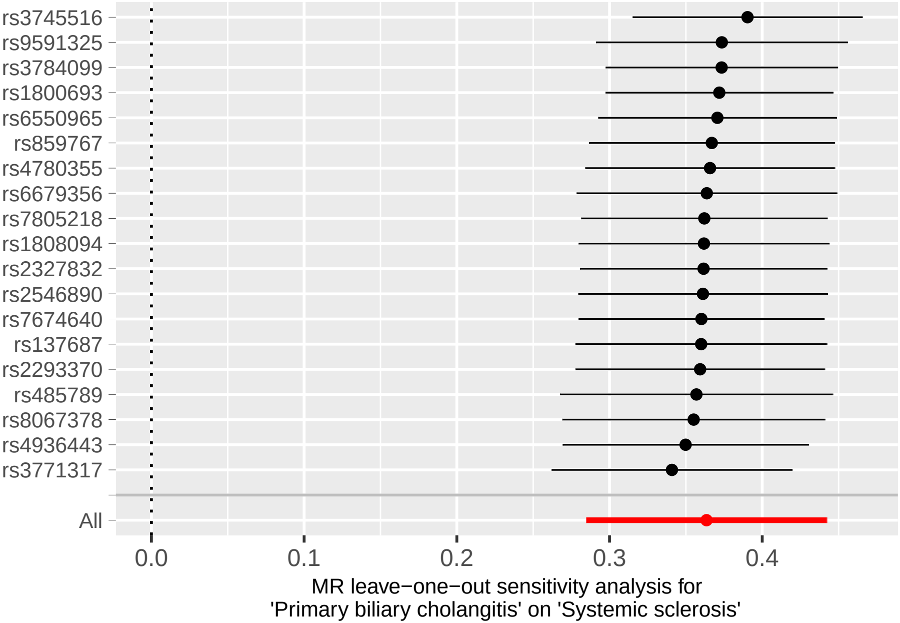

rs3745516
rs9591325
rs3784099
rs1800693
rs6550965
rs859767
rs4780355
rs6679356
rs7805218
rs1808094
rs2327832
rs2546890
rs7674640
rs137687
rs2293370
rs485789
rs8067378
rs4936443
rs3771317
All
0.0
0.1
0.2
0.3
0.4
MR leave−one−out sensitivity analysis for
'Primary biliary cholangitis' on 'Systemic sclerosis'

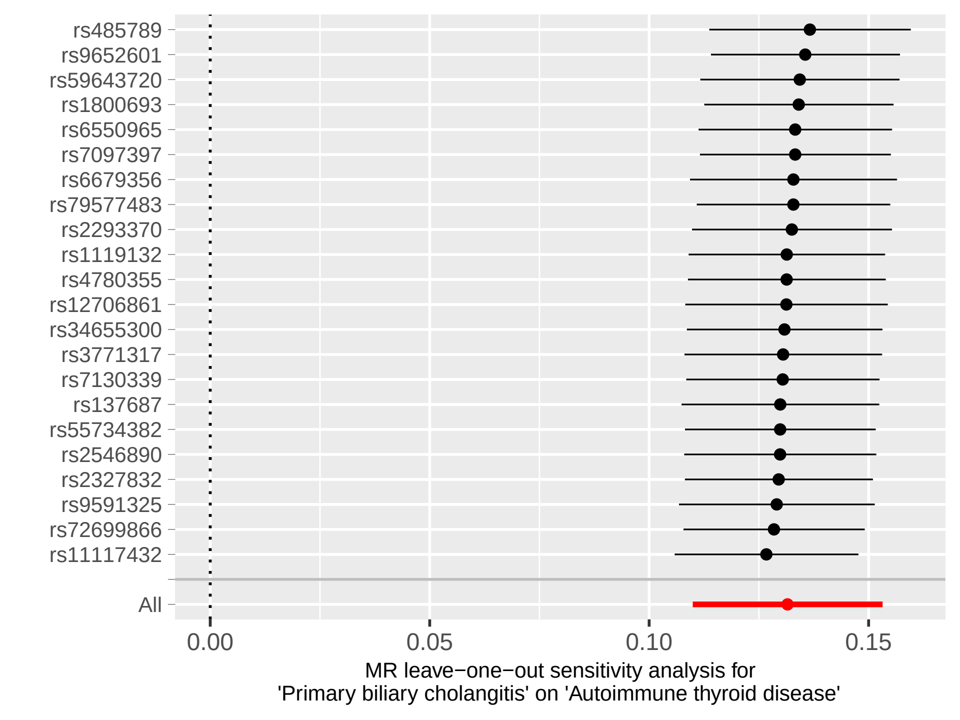
rs485789
rs9652601
rs59643720
rs1800693
rs6550965
rs7097397
rs6679356
rs79577483
rs2293370
rs1119132
rs4780355
rs12706861
rs34655300
rs3771317
rs7130339
rs137687
rs55734382
rs2546890
rs2327832
rs9591325
rs72699866
rs11117432
All
0.00
0.05
0.10
0.15
MR leave−one−out sensitivity analysis for
'Primary biliary cholangitis' on 'Autoimmune thyroid disease'

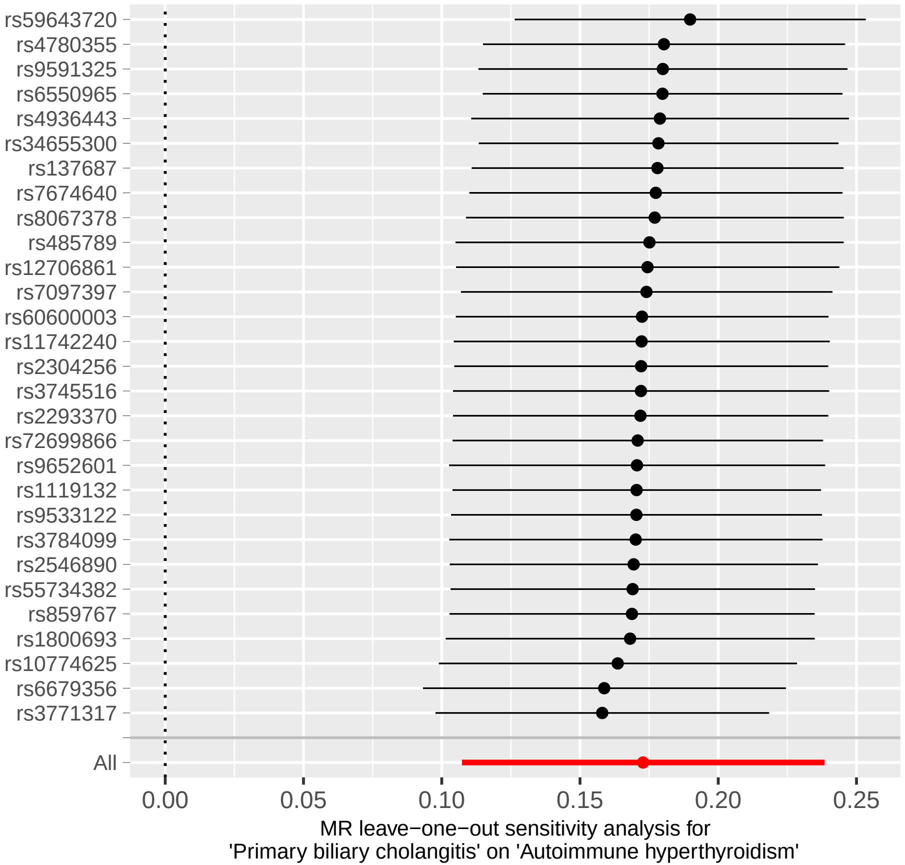
rs59643720
rs4780355
rs9591325
rs6550965
rs4936443
rs34655300
rs137687
rs7674640
rs8067378
rs485789
rs12706861
rs7097397
rs60600003
rs11742240
rs2304256
rs3745516
rs2293370
rs72699866
rs9652601
rs1119132
rs9533122
rs3784099
rs2546890
rs55734382
rs859767
rs1800693
rs10774625
rs6679356
rs3771317
All
0.00
0.05
0.10
0.15
0.20
0.25
MR leave−one−out sensitivity analysis for
'Primary biliary cholangitis' on 'Autoimmune hyperthyroidism'

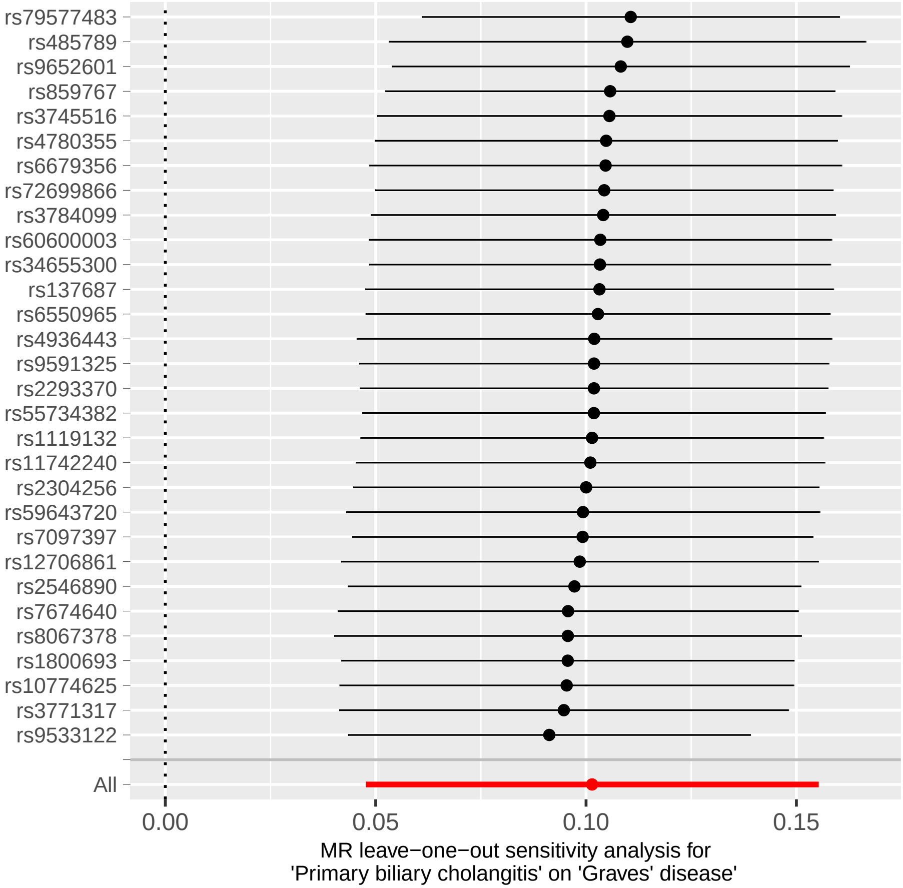
rs79577483
rs485789
rs9652601
rs859767
rs3745516
rs4780355
rs6679356
rs72699866
rs3784099
rs60600003
rs34655300
rs137687
rs6550965
rs4936443
rs9591325
rs2293370
rs55734382
rs1119132
rs11742240
rs2304256
rs59643720
rs7097397
rs12706861
rs2546890
rs7674640
rs8067378
rs1800693
rs10774625
rs3771317
rs9533122
All
0.00
0.05
0.10
0.15
MR leave−one−out sensitivity analysis for
'Primary biliary cholangitis' on 'Graves' disease'

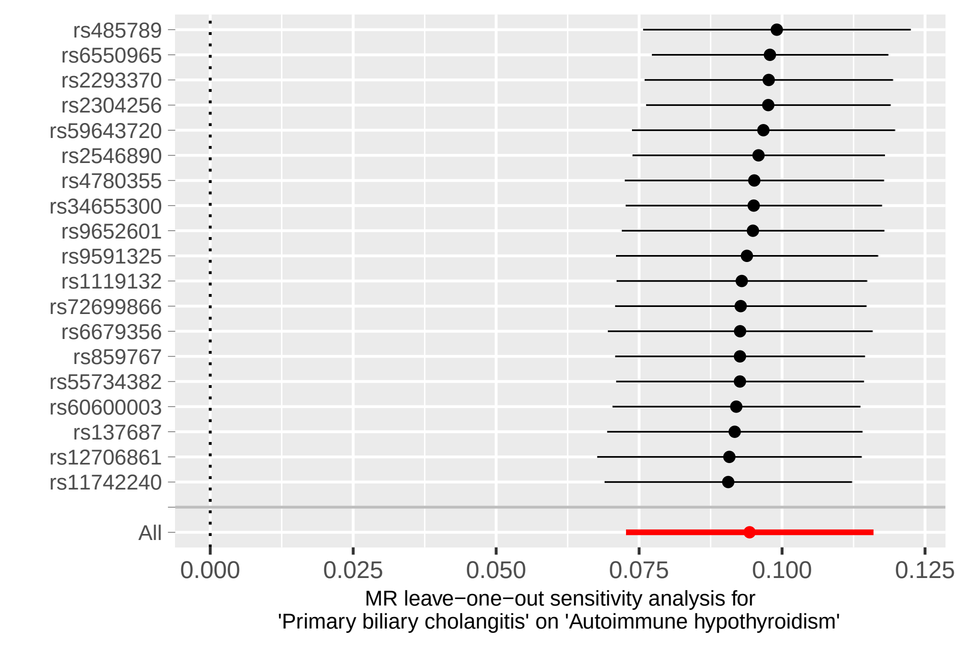

rs485789
rs6550965
rs2293370
rs2304256
rs59643720
rs2546890
rs4780355
rs34655300
rs9652601
rs9591325
rs1119132
rs72699866
rs6679356
rs859767
rs55734382
rs60600003
rs137687
rs12706861
rs11742240
All
0.000
0.025
0.050
0.075
0.100
0.125
MR leave−one−out sensitivity analysis for
'Primary biliary cholangitis' on 'Autoimmune hypothyroidism'

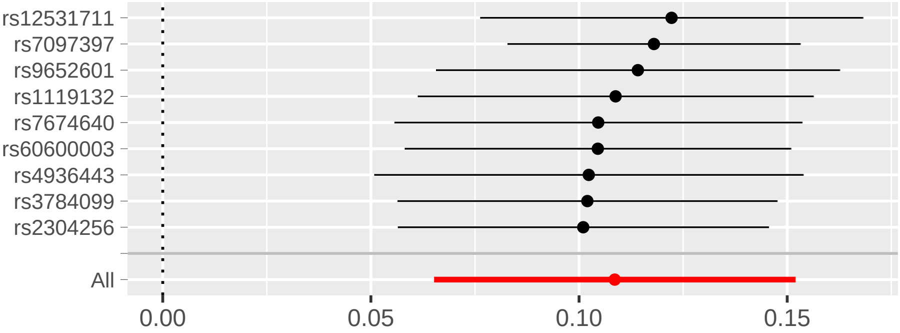

MR leave−one−out sensitivity analysis for
'Primary biliary cholangitis' on 'Inflammatory bowel disease'

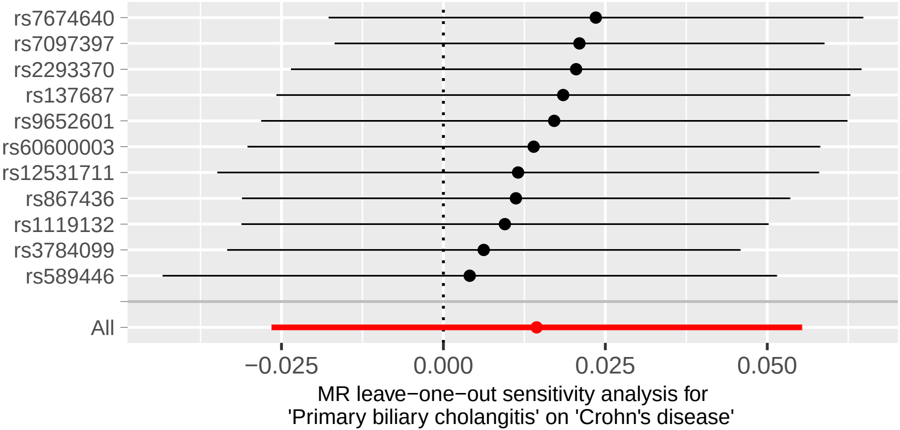
rs7674640
rs7097397
rs2293370
rs137687
rs9652601
rs60600003
rs12531711
rs867436
rs1119132
rs3784099
rs589446
All
−0.025
0.000
0.025
0.050
MR leave−one−out sensitivity analysis for
'Primary biliary cholangitis' on 'Crohn's disease'

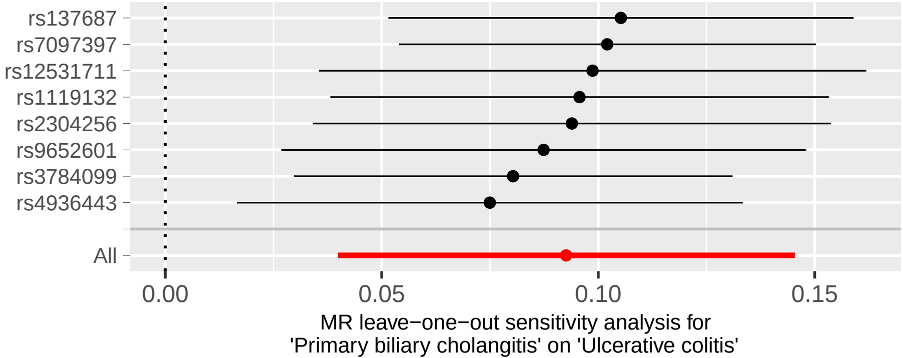
rs137687
rs7097397
rs12531711
rs1119132
rs2304256
rs9652601
rs3784099
rs4936443
All
0.00
0.05
0.10
0.15
MR leave−one−out sensitivity analysis for
'Primary biliary cholangitis' on 'Ulcerative colitis'

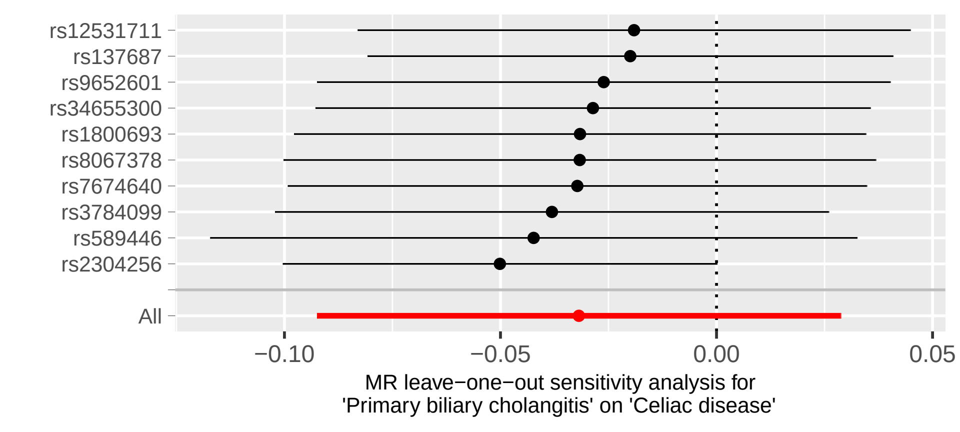
rs12531711
rs137687
rs9652601
rs34655300
rs1800693
rs8067378
rs7674640
rs3784099
rs589446
rs2304256
All
−0.10
−0.05
0.00
0.05
MR leave−one−out sensitivity analysis for
'Primary biliary cholangitis' on 'Celiac disease'

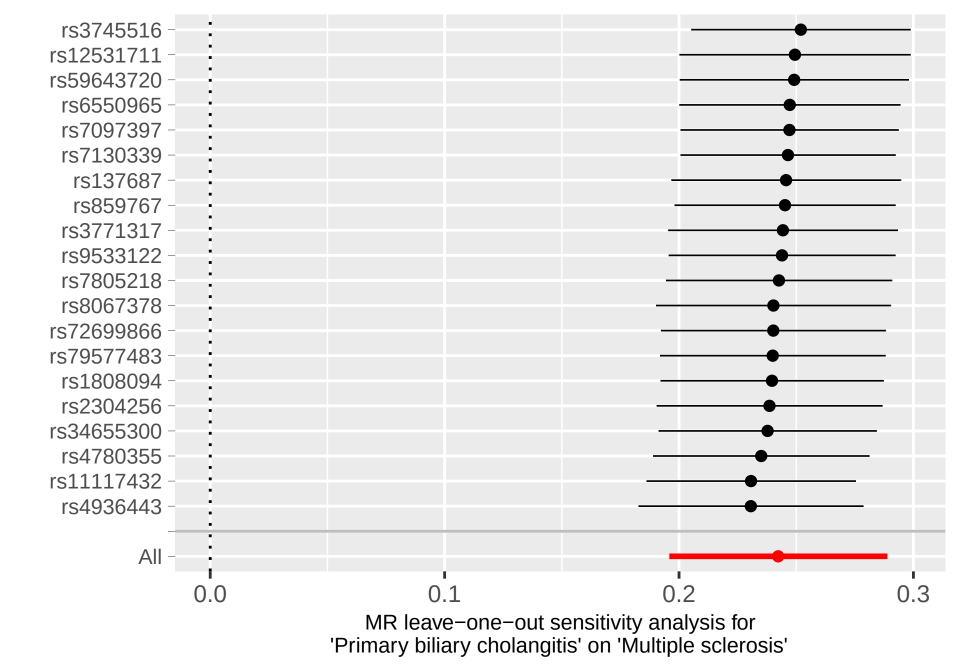
rs3745516
rs12531711
rs59643720
rs6550965
rs7097397
rs7130339
rs137687
rs859767
rs3771317
rs9533122
rs7805218
rs8067378
rs72699866
rs79577483
rs1808094
rs2304256
rs34655300
rs4780355
rs11117432
rs4936443
All
0.0
0.1
0.2
0.3
MR leave−one−out sensitivity analysis for
'Primary biliary cholangitis' on 'Multiple sclerosis'

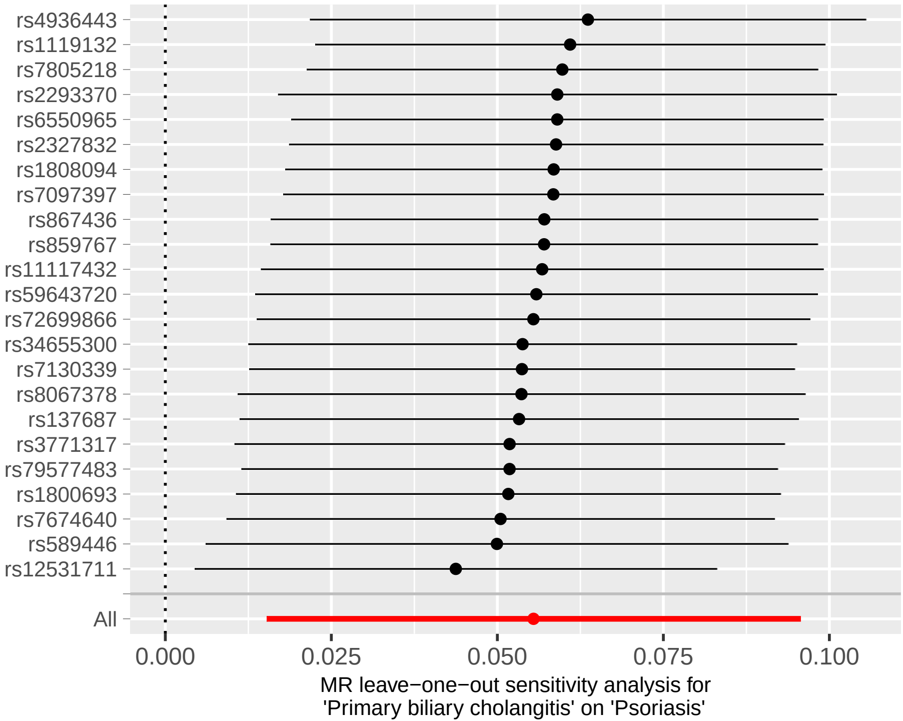
rs4936443
rs1119132
rs7805218
rs2293370
rs6550965
rs2327832
rs1808094
rs7097397
rs867436
rs859767
rs11117432
rs59643720
rs72699866
rs34655300
rs7130339
rs8067378
rs137687
rs3771317
rs79577483
rs1800693
rs7674640
rs589446
rs12531711
All
0.000
0.025
0.050
0.075
0.100
MR leave−one−out sensitivity analysis for
'Primary biliary cholangitis' on 'Psoriasis'

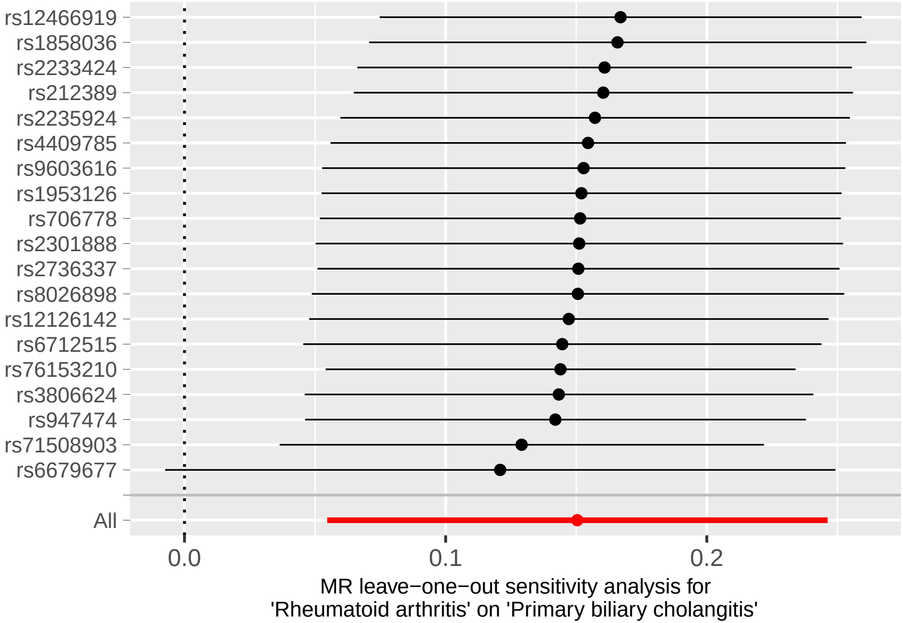
rs12466919
rs1858036
rs2233424
rs212389
rs2235924
rs4409785
rs9603616
rs1953126
rs706778
rs2301888
rs2736337
rs8026898
rs12126142
rs6712515
rs76153210
rs3806624
rs947474
rs71508903
rs6679677
All
0.0
0.1
0.2
MR leave−one−out sensitivity analysis for
'Rheumatoid arthritis' on 'Primary biliary cholangitis'

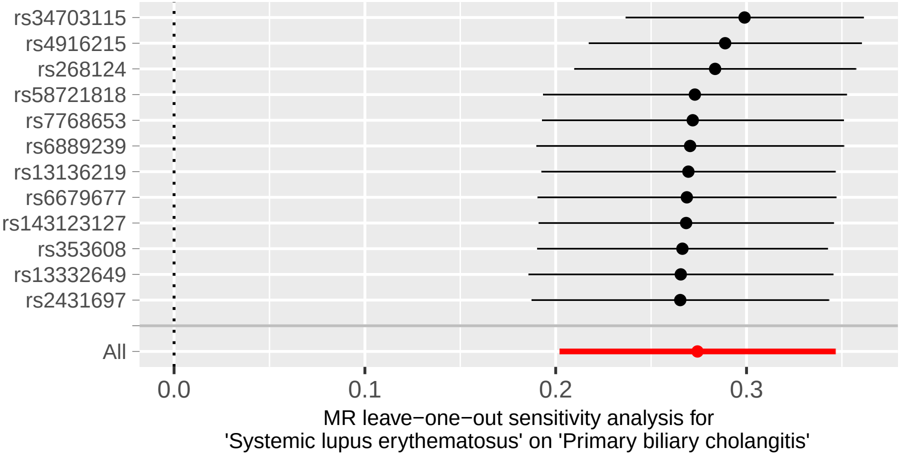

rs34703115
rs4916215
rs268124
rs58721818
rs7768653
rs6889239
rs13136219
rs6679677
rs143123127
rs353608
rs13332649
rs2431697
All
0.0
0.1
0.2
0.3
MR leave−one−out sensitivity analysis for
'Systemic lupus erythematosus' on 'Primary biliary cholangitis'

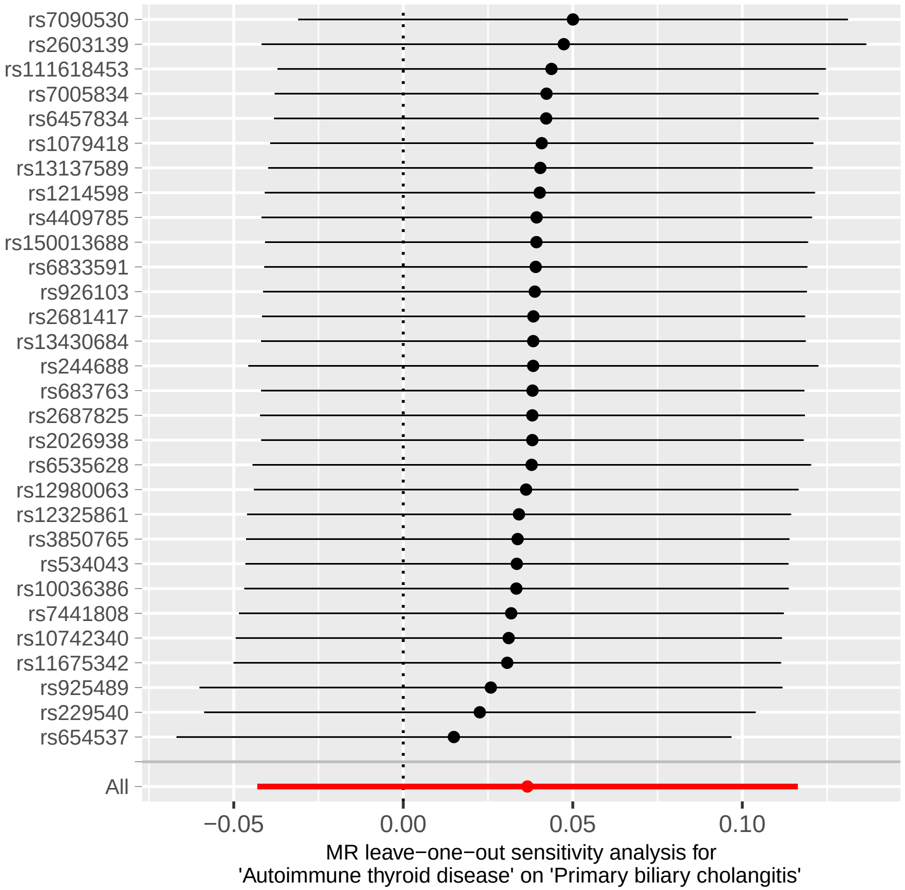
rs7090530
rs2603139
rs111618453
rs7005834
rs6457834
rs1079418
rs13137589
rs1214598
rs4409785
rs150013688
rs6833591
rs926103
rs2681417
rs13430684
rs244688
rs683763
rs2687825
rs2026938
rs6535628
rs12980063
rs12325861
rs3850765
rs534043
rs10036386
rs7441808
rs10742340
rs11675342
rs925489
rs229540
rs654537
All
−0.05
0.00
0.05
0.10
MR leave−one−out sensitivity analysis for
'Autoimmune thyroid disease' on 'Primary biliary cholangitis'

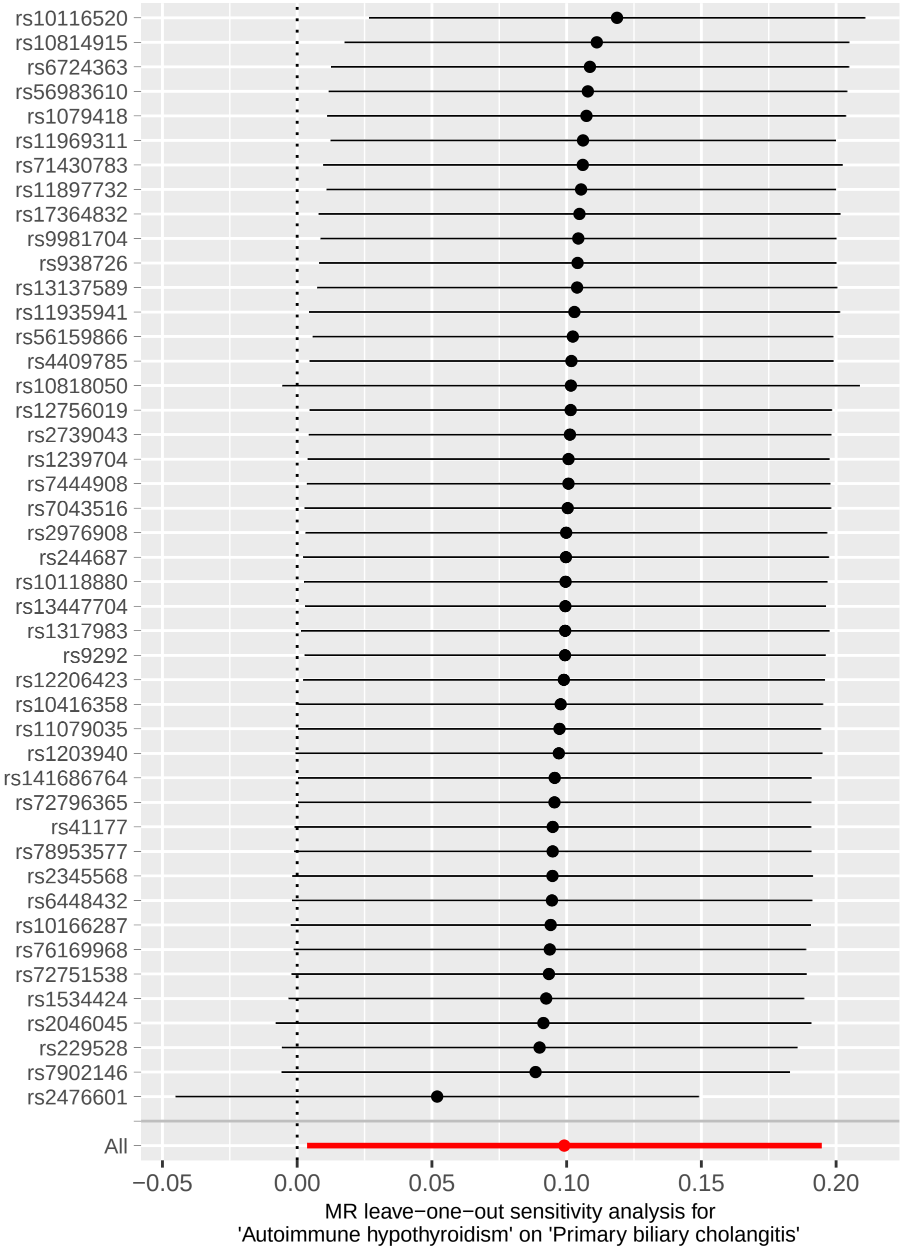
rs10116520
rs10814915
rs6724363
rs56983610
rs1079418
rs11969311
rs71430783
rs11897732
rs17364832
rs9981704
rs938726
rs13137589
rs11935941
rs56159866
rs4409785
rs10818050
rs12756019
rs2739043
rs1239704
rs7444908
rs7043516
rs2976908
rs244687
rs10118880
rs13447704
rs1317983
rs9292
rs12206423
rs10416358
rs11079035
rs1203940
rs141686764
rs72796365
rs41177
rs78953577
rs2345568
rs6448432
rs10166287
rs76169968
rs72751538
rs1534424
rs2046045
rs229528
rs7902146
rs2476601
All
−0.05
0.00
0.05
0.10
0.15
0.20
MR leave−one−out sensitivity analysis for
'Autoimmune hypothyroidism' on 'Primary biliary cholangitis'

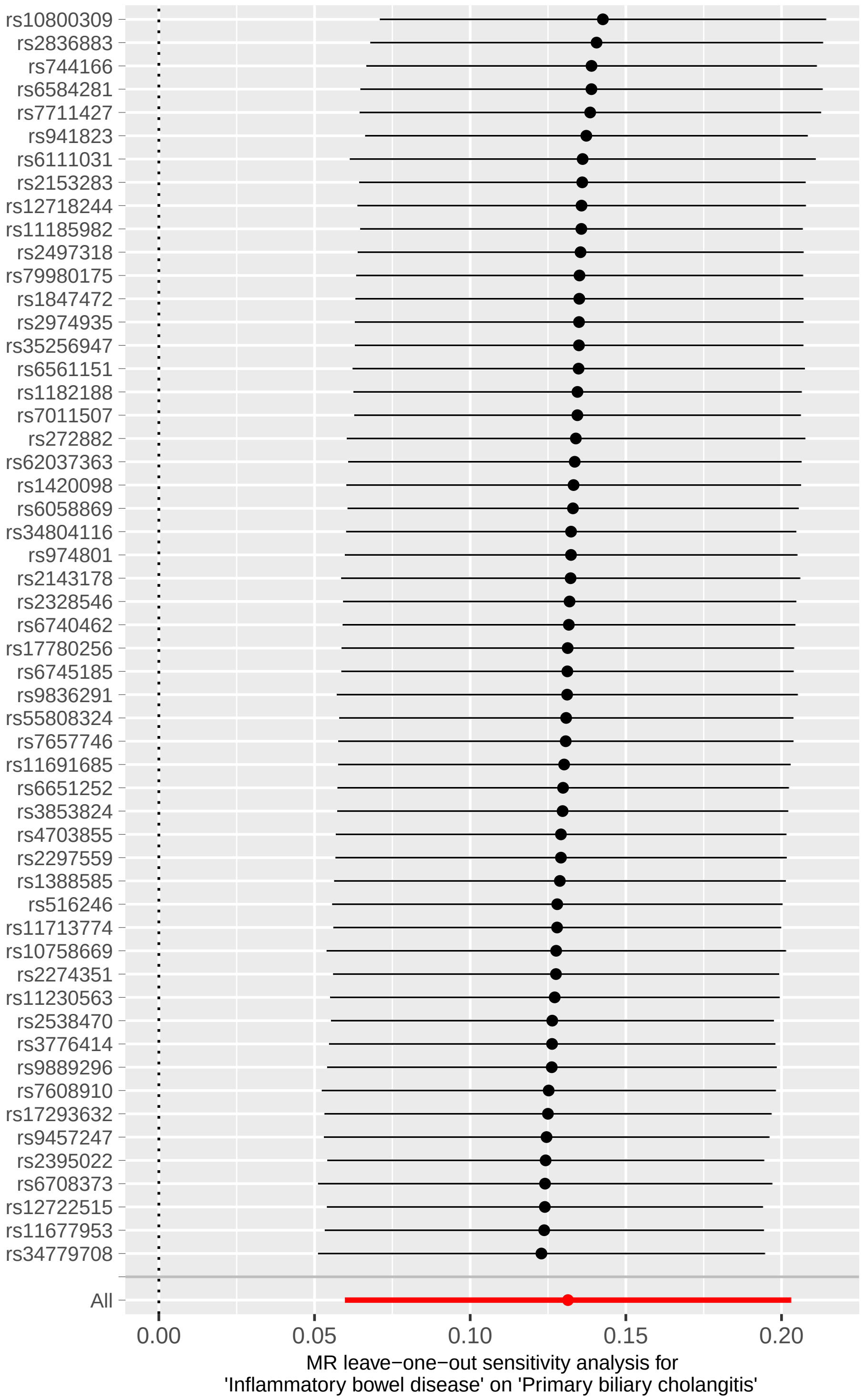
rs10800309
rs2836883
rs744166
rs6584281
rs7711427
rs941823
rs6111031
rs2153283
rs12718244
rs11185982
rs2497318
rs79980175
rs1847472
rs2974935
rs35256947
rs6561151
rs1182188
rs7011507
rs272882
rs62037363
rs1420098
rs6058869
rs34804116
rs974801
rs2143178
rs2328546
rs6740462
rs17780256
rs6745185
rs9836291
rs55808324
rs7657746
rs11691685
rs6651252
rs3853824
rs4703855
rs2297559
rs1388585
rs516246
rs11713774
rs10758669
rs2274351
rs11230563
rs2538470
rs3776414
rs9889296
rs7608910
rs17293632
rs9457247
rs2395022
rs6708373
rs12722515
rs11677953
rs34779708
All
0.00
0.05
0.10
0.15
0.20
MR leave−one−out sensitivity analysis for
'Inflammatory bowel disease' on 'Primary biliary cholangitis'

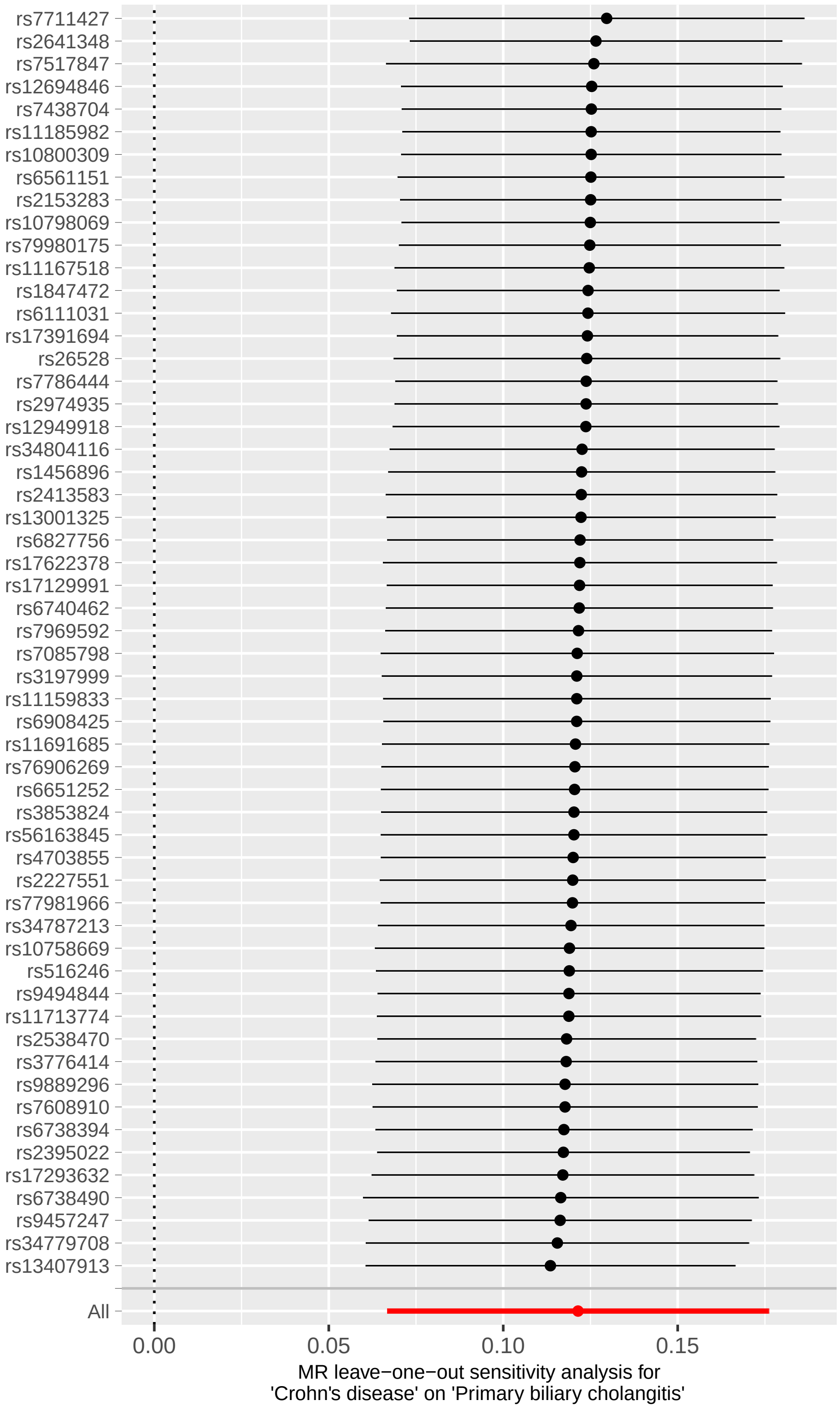
rs7711427
rs2641348
rs7517847
rs12694846
rs7438704
rs11185982
rs10800309
rs6561151
rs2153283
rs10798069
rs79980175
rs11167518
rs1847472
rs6111031
rs17391694
rs26528
rs7786444
rs2974935
rs12949918
rs34804116
rs1456896
rs2413583
rs13001325
rs6827756
rs17622378
rs17129991
rs6740462
rs7969592
rs7085798
rs3197999
rs11159833
rs6908425
rs11691685
rs76906269
rs6651252
rs3853824
rs56163845
rs4703855
rs2227551
rs77981966
rs34787213
rs10758669
rs516246
rs9494844
rs11713774
rs2538470
rs3776414
rs9889296
rs7608910
rs6738394
rs2395022
rs17293632
rs6738490
rs9457247
rs34779708
rs13407913
All
0.00
0.05
0.10
0.15
MR leave−one−out sensitivity analysis for
'Crohn's disease' on 'Primary biliary cholangitis'

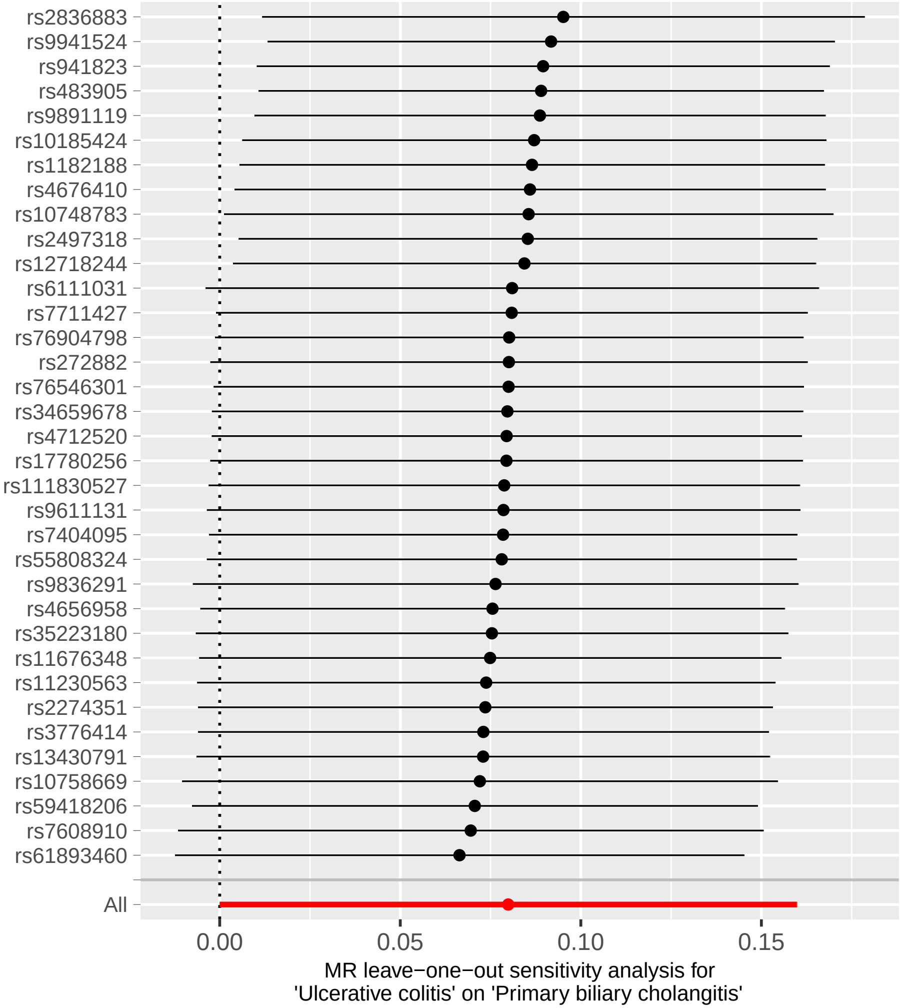
rs2836883
rs9941524
rs941823
rs483905
rs9891119
rs10185424
rs1182188
rs4676410
rs10748783
rs2497318
rs12718244
rs6111031
rs7711427
rs76904798
rs272882
rs76546301
rs34659678
rs4712520
rs17780256
rs111830527
rs9611131
rs7404095
rs55808324
rs9836291
rs4656958
rs35223180
rs11676348
rs11230563
rs2274351
rs3776414
rs13430791
rs10758669
rs59418206
rs7608910
rs61893460
All
0.00
0.05
0.10
0.15
MR leave−one−out sensitivity analysis for
'Ulcerative colitis' on 'Primary biliary cholangitis'

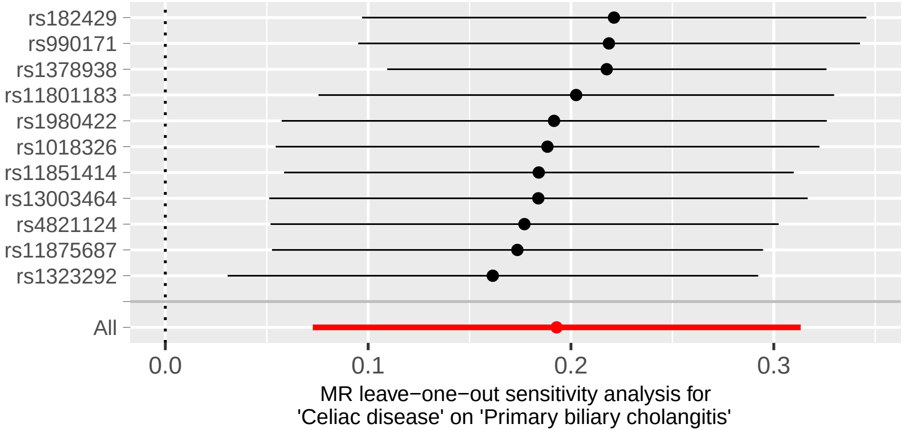
rs182429
rs990171
rs1378938
rs11801183
rs1980422
rs1018326
rs11851414
rs13003464
rs4821124
rs11875687
rs1323292
All
0.0
0.1
0.2
0.3
MR leave−one−out sensitivity analysis for
'Celiac disease' on 'Primary biliary cholangitis'

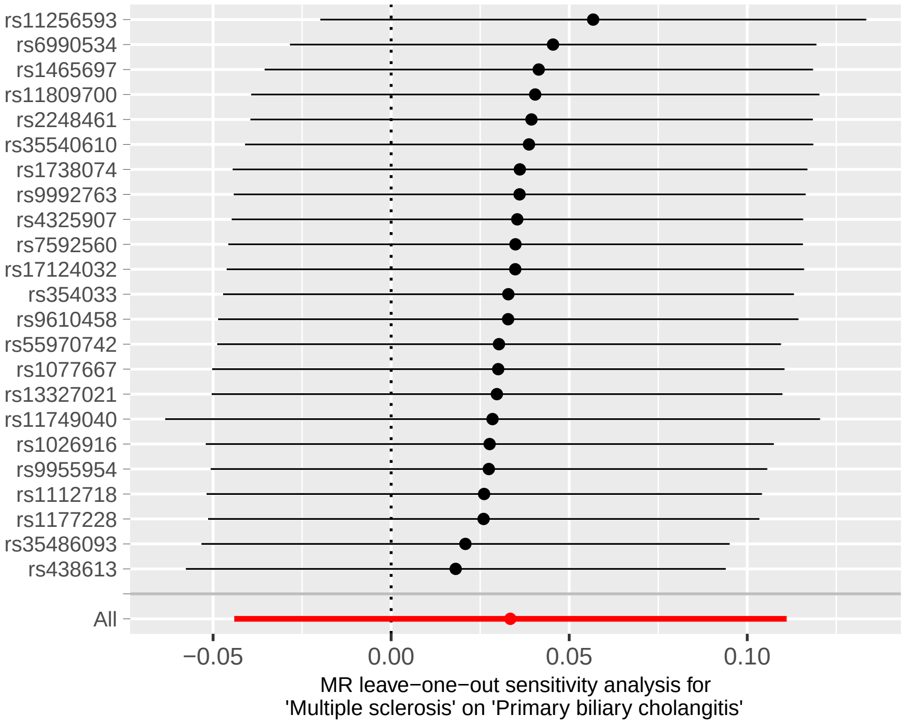
rs11256593
rs6990534
rs1465697
rs11809700
rs2248461
rs35540610
rs1738074
rs9992763
rs4325907
rs7592560
rs17124032
rs354033
rs9610458
rs55970742
rs1077667
rs13327021
rs11749040
rs1026916
rs9955954
rs1112718
rs1177228
rs35486093
rs438613
All
−0.05
0.00
0.05
0.10
MR leave−one−out sensitivity analysis for
'Multiple sclerosis' on 'Primary biliary cholangitis'

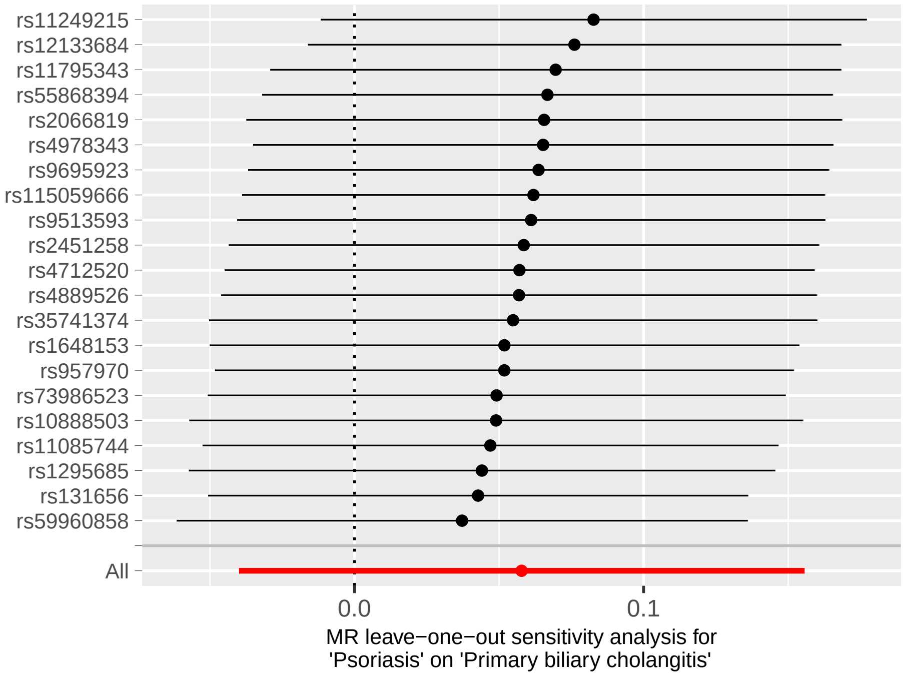
rs11249215
rs12133684
rs11795343
rs55868394
rs2066819
rs4978343
rs9695923
rs115059666
rs9513593
rs2451258
rs4712520
rs4889526
rs35741374
rs1648153
rs957970
rs73986523
rs10888503
rs11085744
rs1295685
rs131656
rs59960858
All
0.0
0.1
MR leave−one−out sensitivity analysis for
'Psoriasis' on 'Primary biliary cholangitis'
